# Supplementary material for: Selective persistence of HIV-1-infected T cell clones can occur through immune reprogramming driven by defective, transcriptionally active proviruses
Source: PLoS Pathog. 2026 Oct 20;22(10):e1014618. doi: 10.1371/journal.ppat.1014618 (PMC13682663; doi:10.1371/journal.ppat.1014618)
Supplement: Supplementary file 1 [file ppat.1014618.s008.pdf]

LV_BACH2_vs_GFP_d4

baseMean	log2FoldChange	lfcSE	stat	pvalue	padj	ENSEMBL	SYMBOL
132,3226787	-1,34533462	0,332479653	-4,046366766	5,20187E-05	0,034398565	ENSG00000087842	PIR
31,97700974	3,924563643	0,970519567	4,043775907	5,25972E-05	0,034398565	ENSG00000284294	LOC122455341
83,31786793	-1,638653025	0,398985997	-4,107043948	4,00755E-05	0,030577607	ENSG00000100292	HMOX1
64,14997557	-2,034695066	0,488884041	-4,161917541	3,15586E-05	0,025495662	ENSG00000185304	RGPD2
198,5645871	-1,184873724	0,276210478	-4,28974937	1,78875E-05	0,015354171	ENSG00000126822	PLEKHG3
169,4540464	1,605085215	0,371454882	4,32107718	1,55269E-05	0,014216461	ENSG00000015568	RGPD5
115,9304322	1,510924286	0,342193726	4,415406163	1,00821E-05	0,010651299	ENSG00000131401	ENSG00000131401
330,8513753	-1,114017698	0,238575398	-4,669457572	3,01996E-06	0,00368999	ENSG00000205336	ADGRG1
65,16185864	2,244297214	0,479129466	4,684114367	2,81173E-06	0,00368999	ENSG00000075391	RASAL2
325,519042	1,060407617	0,223306617	4,748661862	2,04767E-06	0,003515337	ENSG00000134852	CLOCK
322,7546624	1,224972866	0,247659867	4,946190433	7,56799E-07	0,001732314	ENSG00000170100	ZNF778
232,5551839	1,483782351	0,2913395	5,092966634	3,52504E-07	0,000968257	ENSG00000162614	NEXN
174,2403185	-1,521411869	0,283946313	-5,358096941	8,41031E-08	0,000552605	ENSG00000138678	GPAT3
11238,08548	6,645958138	0,149218337	44,53848148	0	0	ENSG00000112182	BACH2

baseMean	log2FoldChange	lfcSE	stat	pvalue	padj	ENSEMBL	SYMBOL
44,13857273	18,71703136	3,456370371	5,41522735	6,12109E-08	1,63157E-05	ENSG00000244398	RPL36AP37
5815,33448	4,297471781	0,862358084	4,983395947	6,24779E-07	0,000111023	ENSG00000112182	BACH2
8,791258653	3,551002791	0,915024507	3,880773424	0,000104125	0,007751201	ENSG00000148143	ZNF462
15,10702187	3,327938892	0,791330237	4,205499468	2,60506E-05	0,002547412	ENSG00000227165	WDR11-DT
16,30520908	3,306090298	0,862339436	3,833861889	0,000126147	0,008871086	ENSG00000070182	SPTB
16,17256435	3,251547738	0,634923315	5,121166072	3,03652E-07	6,1197E-05	ENSG00000104722	NEFM
12,90066212	3,22376499	0,839724455	3,839074795	0,000123499	0,008787942	ENSG00000156011	PSD3
16,38587118	2,834031525	0,853681028	3,319778034	0,00090089	0,038670431	ENSG00000269896	ENSG00000269896
8,668090011	2,759932412	0,80488447	3,42897958	0,000605855	0,029798695	ENSG00000240591	ENSG00000240591
18,1024471	2,352688763	0,597199971	3,939532617	8,16405E-05	0,006404942	ENSG00000181577	LINC03040
33,13946661	2,245308759	0,521326831	4,306911953	1,65549E-05	0,001776539	ENSG00000198626	RYR2
23,88802206	2,073128382	0,492577026	4,208739488	2,56799E-05	0,00252611	ENSG00000175841	ENSG00000175841
20,41718282	2,060189809	0,583447629	3,531062097	0,000413895	0,021714354	ENSG00000159871	LYPD5
18,19544252	1,942145831	0,602906114	3,221307243	0,001276073	0,04985432	ENSG00000136630	HLX
34,60957483	1,798777441	0,543149157	3,311755929	0,000927124	0,039590827	ENSG00000248503	ENSG00000248503
37,89304733	1,765992435	0,44365189	3,98058134	6,87469E-05	0,005624316	ENSG00000126878	AIF1L
185,1634291	1,661663464	0,334117493	4,973290833	6,58258E-07	0,000115727	ENSG00000162614	NEXN
19,59493704	1,649494032	0,509602171	3,236826932	0,001208667	0,048481624	ENSG00000161921	CXCL16
32,86940289	1,644952333	0,435074026	3,780856214	0,00015629	0,010500654	ENSG00000141622	ARK2C
86,59759961	1,598312145	0,345880817	4,620991004	3,81911E-06	0,000553637	ENSG00000099958	DERL3
41,29847897	1,575973298	0,377831163	4,171104593	3,03127E-05	0,00283021	ENSG00000008441	NFIX
102,9529174	1,560996567	0,276031517	5,655138894	1,5572E-08	5,04595E-06	ENSG00000237989	LINC01679
25,78725365	1,384336913	0,421809588	3,281900065	0,001031101	0,043139195	ENSG00000167034	NKX3-1
199,6800934	1,370195433	0,26348416	5,200295287	1,98972E-07	4,3266E-05	ENSG00000066923	STAG3
140,1555988	1,367868334	0,214432104	6,379027716	1,78216E-10	9,50063E-08	ENSG00000160190	SLC37A1
105,1001655	1,364962265	0,313011631	4,360739761	1,29623E-05	0,001509023	ENSG00000204219	TCEA3
33,5045346	1,357650996	0,401114738	3,384694867	0,000712574	0,03326554	ENSG00000102996	MMP15
50,34883678	1,322789648	0,352624366	3,751271251	0,00017594	0,011677061	ENSG00000214595	EML6
516,9257723	1,287937889	0,333012944	3,867531019	0,000109943	0,008004033	ENSG00000137959	IFI44L
40,81415639	1,272952238	0,346927962	3,669211994	0,000243299	0,01505904	ENSG00000119922	IFIT2
518,0824024	1,227500625	0,22779226	5,388684513	7,09753E-08	1,8474E-05	ENSG00000145287	PLAC8
169,6710014	1,214229265	0,339546271	3,576034754	0,000348845	0,019485718	ENSG00000164187	LMBRD2
80,15230359	1,168863564	0,246057468	4,75036817	2,03047E-06	0,000313603	ENSG00000139832	RAB20
47,75580475	1,148755849	0,320289374	3,586618665	0,000334994	0,019090018	ENSG00000140479	PCSK6
240,015253	1,128804411	0,237296666	4,756933285	1,96556E-06	0,000309361	ENSG00000270055	ENSG00000270055
129,9173224	1,127898513	0,332861095	3,388496076	0,00070277	0,032994269	ENSG00000157483	MYO1E
4981,928425	1,068926972	0,203640473	5,24908903	1,52853E-07	3,66094E-05	ENSG00000128342	LIF
92,6102906	1,061415169	0,246606286	4,30408805	1,67675E-05	0,00178774	ENSG00000081181	ARG2
103,0732471	1,040274777	0,253685265	4,100651167	4,11989E-05	0,003602399	ENSG00000159128	IFNGR2
693,9494153	1,039479754	0,299288112	3,473174212	0,000514341	0,026153863	ENSG00000171316	CHD7
1125,226742	1,032024575	0,26372559	3,913251556	9,10616E-05	0,006967054	ENSG00000112972	HMGCS1
59,05535073	1,01201789	0,30345095	3,335029564	0,000852903	0,037092316	ENSG00000158457	TSPAN33
84,37139726	-1,001779757	0,264963201	-3,780825988	0,000156309	0,010500654	ENSG00000196387	ZNF140
112,8151397	-1,006685789	0,300330566	-3,35192585	0,000802515	0,035797137	ENSG00000198964	SGMS1
211,9669699	-1,023498174	0,265272815	-3,858285193	0,000114185	0,008240294	ENSG00000138185	ENTPD1
1118,040745	-1,029588059	0,145184912	-7,091563771	1,32605E-12	9,96104E-10	ENSG00000132965	ALOX5AP
325,2048504	-1,029820941	0,191834005	-5,368291936	7,94858E-08	2,0209E-05	ENSG00000105374	NKG7
4940,392438	-1,038485534	0,163208267	-6,362946893	1,97919E-10	1,02213E-07	ENSG00000178209	PLEC
1785,320807	-1,04786729	0,186354844	-5,622967809	1,87704E-08	5,96539E-06	ENSG00000178573	MAF
71,56146681	-1,049134797	0,276898957	-3,788872334	0,000151333	0,010334394	ENSG00000168672	LRATD2
121,4243083	-1,052132353	0,303276589	-3,469217179	0,000521977	0,026460727	ENSG00000238121	LINC00426
1352,242598	-1,055738707	0,181928478	-5,803042607	6,51222E-09	2,33959E-06	ENSG00000185989	RASA3
467,1279788	-1,075555706	0,284059992	-3,786368148	0,000152865	0,010396088	ENSG00000111145	ELK3
407,8068259	-1,086876307	0,190773231	-5,697216025	1,2178E-08	4,02506E-06	ENSG00000092871	RFFL
146,3092451	-1,107554542	0,340598241	-3,251791722	0,0011468	0,046679854	ENSG00000178163	ZNF518B
8463,248238	-1,110340101	0,146429545	-7,58276003	3,3828E-14	4,30032E-11	ENSG00000087086	FTL
252,9308555	-1,128598518	0,217941702	-5,17844226	2,23746E-07	4,71559E-05	ENSG00000121807	CCR2
167,3973015	-1,131237047	0,27325176	-4,139907628	3,47446E-05	0,003141398	ENSG00000178996	SNX18
681,5577511	-1,1346807	0,245955678	-4,613354354	3,96222E-06	0,00056448	ENSG00000133816	MICAL2
128,6043548	-1,136139761	0,266124636	-4,269201756	1,96174E-05	0,002051878	ENSG00000121039	RDH10
1071,526465	-1,138258147	0,223562575	-5,091452131	3,55332E-07	6,90848E-05	ENSG00000091592	NLRP1
89,61667066	-1,14450837	0,274192793	-4,174100848	2,99165E-05	0,002809093	ENSG00000059377	TBXAS1
506,599375	-1,147474878	0,165774443	-6,921904601	4,45611E-12	3,06841E-09	ENSG00000120885	CLU
149,5084438	-1,154191222	0,316679053	-3,644671826	0,000267733	0,016148026	ENSG00000163867	ZMYM6
178,3257652	-1,158963044	0,318607127	-3,637592968	0,000275198	0,016477971	ENSG00000132359	RAP1GAP2
312,8498236	-1,162211067	0,314895843	-3,690779319	0,000223568	0,014210328	ENSG00000139278	GLIPR1
96,42440607	-1,176695223	0,312247433	-3,768470447	0,000164251	0,010945205	ENSG00000101842	VSIG1
166,851967	-1,179606879	0,214655278	-5,495354651	3,89926E-08	1,1507E-05	ENSG00000016391	CHDH

392,6864253	-1,197249221	0,278741133	-4,295201105	1,74535E-05	0,001848953	ENSG00000136111	TBC1D4
92,81231688	-1,199885757	0,286609751	-4,186479182	2,83315E-05	0,002722129	ENSG00000012779	ALOX5
354,3565056	-1,218846146	0,243680109	-5,001828625	5,67891E-07	0,000104277	ENSG00000157978	LDLRAP1
831,2996755	-1,225370632	0,178294232	-6,872744125	6,29785E-12	4,00301E-09	ENSG00000064012	CASP8
40,72242247	-1,226681319	0,344907701	-3,556549523	0,000375758	0,020359906	ENSG00000179104	TMTC2
93,83917832	-1,22933486	0,334852148	-3,671276613	0,000241342	0,015050631	ENSG00000183044	ABAT
1877,812044	-1,250768804	0,213056902	-5,870585705	4,34258E-09	1,7087E-06	ENSG00000110324	IL10RA
321,8905651	-1,256011299	0,258915529	-4,851046612	1,22812E-06	0,000200949	ENSG00000114554	PLXNA1
254,6136419	-1,258245449	0,202819825	-6,20375967	5,513E-10	2,60308E-07	ENSG00000166963	MAP1A
36,34619421	-1,262076481	0,368844907	-3,421699628	0,00062231	0,030426919	ENSG00000284634	ENSG00000284634
75,53847343	-1,264611463	0,32947382	-3,838276026	0,000123901	0,008787942	ENSG00000267364	ENSG00000267364
72,32461973	-1,268619222	0,391192284	-3,242955636	0,001182966	0,047682198	ENSG00000104447	TRPS1
50,77130273	-1,272205827	0,351701468	-3,617288926	0,000297705	0,017446353	ENSG00000161551	ZNF577
62,39979072	-1,272438576	0,316819576	-4,016287728	5,91221E-05	0,004934602	ENSG00000164849	GPR146
53,21830826	-1,275658907	0,322626832	-3,953976487	7,6863E-05	0,006166201	ENSG00000259436	POU2F2-AS2
111,8692555	-1,27780781	0,387210544	-3,300033615	0,000966732	0,0408599	ENSG00000282508	ENSG00000282508
473,1150843	-1,278408772	0,18555011	-6,889830324	5,5859E-12	3,6925E-09	ENSG00000169194	IL13
101,860909	-1,279761468	0,375032532	-3,412401218	0,000643933	0,031003711	ENSG00000262580	ENSG00000262580
67,64158294	-1,283674469	0,359142984	-3,574271327	0,000351205	0,019542112	ENSG00000267041	ZNF850
1210,062938	-1,287990926	0,243043007	-5,29943627	1,16161E-07	2,84946E-05	ENSG00000165272	AQP3
154,3280967	-1,295840213	0,248072614	-5,223632684	1,75447E-07	4,12125E-05	ENSG00000177034	MTX3
135,1042019	-1,296550952	0,257205464	-5,040915273	4,63311E-07	8,66863E-05	ENSG00000164120	HPGD
35,6019444	-1,297211078	0,388369337	-3,34014804	0,000837337	0,036900902	ENSG00000284879	ENSG00000284879
68,54190975	-1,297576762	0,28476613	-4,556640086	5,19784E-06	0,000704094	ENSG00000102349	KLF8
221,3079071	-1,306943155	0,251023119	-5,206465284	1,92472E-07	4,29836E-05	ENSG00000110665	C11orf21
47,33535919	-1,314527673	0,38475247	-3,416554218	0,00063419	0,030825381	ENSG00000167094	TTC16
100,2121555	-1,314757226	0,338301592	-3,886346549	0,000101764	0,007609752	ENSG00000118515	SGK1
142,9536394	-1,319912276	0,292627508	-4,510554338	6,46584E-06	0,000825262	ENSG00000111537	IFNG
526,8199341	-1,320719872	0,20353556	-6,488890052	8,6471E-11	4,92766E-08	ENSG00000160791	CCR5
236,8407704	-1,336547515	0,265216364	-5,039460975	4,66845E-07	8,66863E-05	ENSG00000113369	ARRDC3
71,19542894	-1,363842614	0,396559931	-3,439184112	0,00058347	0,028828342	ENSG00000103253	HAGHL
13155,0133	-1,385929536	0,207693817	-6,672945575	2,50719E-11	1,53459E-08	ENSG00000173821	RNF213
227,2513863	-1,386654409	0,2405833	-5,763718471	8,22806E-09	2,89313E-06	ENSG00000245164	LINC00861
78,4298876	-1,395511793	0,302375531	-4,615161115	3,9279E-06	0,000564457	ENSG00000132694	ARHGEF11
227,9545778	-1,400230731	0,26845389	-5,215907778	1,82919E-07	4,19449E-05	ENSG00000181523	SGSH
807,9090356	-1,403932137	0,238375443	-5,88958376	3,8717E-09	1,56058E-06	ENSG00000169756	LIMS1
102,1601069	-1,414575506	0,29668625	-4,767917302	1,8614E-06	0,000298656	ENSG00000124212	PTGIS
109,1188809	-1,42457599	0,234946562	-6,063404269	1,3327E-09	5,95249E-07	ENSG00000275302	CCL4
64,61917636	-1,439038302	0,396914153	-3,625565611	0,00028833	0,016957068	ENSG00000177337	ENSG00000177337
164,8496964	-1,440826019	0,22513791	-6,399748576	1,55633E-10	8,5733E-08	ENSG00000178860	MSC
169,9425458	-1,454418399	0,28535541	-5,096866394	3,45322E-07	6,87565E-05	ENSG00000141540	TTYH2
97,03919296	-1,474995717	0,296892726	-4,968109982	6,76086E-07	0,000117611	ENSG00000171621	SPSB1
82,40956536	-1,478380094	0,269834935	-5,478831329	4,28144E-08	1,24132E-05	ENSG00000164400	CSF2
478,6034415	-1,481350639	0,199192781	-7,436768697	1,03178E-13	1,0657E-10	ENSG00000077984	CST7
82,74910346	-1,48391576	0,275449601	-5,387249617	7,1544E-08	1,8474E-05	ENSG00000107968	MAP3K8
26,65965449	-1,515778215	0,414907563	-3,653291359	0,0002589	0,015788134	ENSG00000178038	ALS2CL
108,5400923	-1,523401166	0,421821011	-3,611487159	0,000304446	0,017778365	ENSG00000064201	TSPAN32
263,5330376	-1,530155885	0,229829654	-6,657782651	2,77989E-11	1,64073E-08	ENSG00000164483	SAMD3
156,4450422	-1,531990196	0,350513604	-4,370701098	1,23848E-05	0,001472457	ENSG00000196218	RYR1
22,1719237	-1,538378621	0,461197693	-3,335616467	0,000851105	0,037092316	ENSG00000154027	AK5
418,7222962	-1,538691556	0,205875104	-7,473907831	7,78477E-14	8,57674E-11	ENSG00000048471	SNX29
90,42639405	-1,547513094	0,325503139	-4,754218654	1,99215E-06	0,000310588	ENSG00000073910	FRY
39,29625821	-1,563842509	0,470152826	-3,32624292	0,000880252	0,037882919	ENSG00000104497	SNX16
47,25841295	-1,565513751	0,32083972	-4,879426247	1,06395E-06	0,000175828	ENSG00000230438	ENSG00000230438
98,85276317	-1,576419925	0,309453639	-5,094203868	3,5021E-07	6,88996E-05	ENSG00000180549	FUT7
282,8700113	-1,582298196	0,215922857	-7,328071771	2,33488E-13	2,26977E-10	ENSG00000171476	HOPX
50,86261052	-1,589379251	0,383591939	-4,143411495	3,42177E-05	0,003141398	ENSG00000248323	LUCAT1
129,3910187	-1,600866349	0,363772636	-4,400733285	1,07886E-05	0,0013014	ENSG00000171246	NPTX1
556,6763638	-1,607735808	0,204241178	-7,871751563	3,4971E-15	4,81609E-12	ENSG00000172965	MIR4435-2HG
39,75714613	-1,630169152	0,456775603	-3,568862129	0,000358535	0,019816555	ENSG00000006704	GTF2IRD1
541,3178522	-1,638199602	0,170592146	-9,60301892	7,76365E-22	3,20755E-18	ENSG00000057657	PRDM1
19,67134573	-1,641847326	0,507928947	-3,232435042	0,0012274	0,04875966	ENSG00000271361	ENSG00000271361
30,73537779	-1,675834996	0,397413391	-4,216855886	2,47732E-05	0,002466278	ENSG00000172348	RCAN2
151,9297589	-1,684769588	0,310642769	-5,423495276	5,84448E-08	1,58337E-05	ENSG00000059728	MXD1
66,26644782	-1,727295674	0,332500507	-5,194866286	2,04867E-07	4,39693E-05	ENSG00000013619	MAMLD1
22,28621106	-1,732563622	0,501202826	-3,456811358	0,000546607	0,027624566	ENSG00000204577	LILRB3
126,8362688	-1,733556905	0,240173012	-7,217950475	5,27769E-13	4,59048E-10	ENSG00000277632	CCL3
131,2402435	-1,738793355	0,335865664	-5,177050054	2,25422E-07	4,71559E-05	ENSG00000181019	NQO1
33,19544873	-1,740256252	0,496064217	-3,508126958	0,000451274	0,023305463	ENSG00000163106	HPGDS

345,504096	-1,781666403	0,187118022	-9,521618403	1,70503E-21	5,63546E-18	ENSG00000222041	CYTOR
23,29182627	-1,851208283	0,477569482	-3,876311934	0,000106052	0,007824154	ENSG00000196422	PPP1R26
54,47457758	-1,890260479	0,374932822	-5,041597772	4,61661E-07	8,66863E-05	ENSG00000205336	ADGRG1
470,5437404	-1,907597377	0,231777376	-8,230300182	1,86745E-16	3,08615E-13	ENSG00000139679	LPAR6
524,1211841	-1,908840774	0,185181986	-10,30791824	6,48917E-25	3,57467E-21	ENSG00000033327	GAB2
18,84003797	-1,91100536	0,568279383	-3,362791994	0,000771585	0,034935358	ENSG00000184731	FAM110C
194,4843739	-1,911949746	0,265777329	-7,193803006	6,30111E-13	4,95867E-10	ENSG00000136161	RCBTB2
56,52260023	-1,913056493	0,39125807	-4,889500407	1,01092E-06	0,000168753	ENSG00000115594	IL1R1
56,82871009	-1,918833604	0,323420251	-5,932942036	2,97554E-09	1,22934E-06	ENSG00000164530	PI16
95,31302809	-1,926554125	0,330505191	-5,829119108	5,57207E-09	2,09243E-06	ENSG00000186594	MIR22HG
30,90899801	-1,932081442	0,426072828	-4,534627221	5,77053E-06	0,00076291	ENSG00000152217	SETBP1
366,4839823	-1,937341739	0,258155931	-7,504540889	6,16443E-14	7,27667E-11	ENSG00000139289	PHLDA1
136,4824054	-1,996704546	0,247287257	-8,074433631	6,77906E-16	1,01846E-12	ENSG00000111536	IL26
39,56762463	-2,003474518	0,426910318	-4,692963448	2,69276E-06	0,000408261	ENSG00000269001	ENSG00000269001
133,9789227	-2,005904873	0,278554625	-7,201118521	5,97206E-13	4,93471E-10	ENSG00000188322	SBK1
28,85750427	-2,033423202	0,630018212	-3,227562576	0,001248497	0,049125396	ENSG00000229212	ENSG00000229212
18,91028224	-2,083111146	0,581037507	-3,585157792	0,000336874	0,019131228	ENSG00000112115	IL17A
34,01744177	-2,086234669	0,623986439	-3,343397451	0,000827593	0,036667018	ENSG00000106070	GRB10
150,1263163	-2,092494046	0,382907898	-5,46474506	4,63573E-08	1,32086E-05	ENSG00000181045	SLC26A11
109,344027	-2,122209469	0,338859104	-6,262807894	3,78106E-10	1,83782E-07	ENSG00000100600	LGMN
23,44646447	-2,131814257	0,500196528	-4,261953324	2,02648E-05	0,002080098	ENSG00000224383	PRR29
84,63912429	-2,156434126	0,297386346	-7,251288284	4,12826E-13	3,7902E-10	ENSG00000134531	EMP1
12,06436884	-2,187492833	0,651473708	-3,357760727	0,000785766	0,035383021	ENSG00000163464	CXCR1
24,95542206	-2,199806206	0,502522048	-4,37753172	1,20031E-05	0,001437413	ENSG00000008226	DLEC1
241,9148783	-2,209864439	0,317403195	-6,962325753	3,347E-12	2,4049E-09	ENSG00000126822	PLEKHG3
21,2150224	-2,215674295	0,509592995	-4,347929263	1,37429E-05	0,001545	ENSG00000198848	CES1
55,62621736	-2,245634228	0,430732174	-5,213527948	1,85283E-07	4,19449E-05	ENSG00000143776	CDC42BPA
25,78131236	-2,292088795	0,52628716	-4,355205612	1,32942E-05	0,001515173	ENSG00000279765	ENSG00000279765
27,41919022	-2,324347304	0,588873931	-3,947105115	7,91018E-05	0,006254722	ENSG00000062524	LTK
51,17980613	-2,32857413	0,576277537	-4,040716459	5,32882E-05	0,004518853	ENSG00000123836	PFKFB2
93,34250186	-2,366804338	0,287254769	-8,239390931	1,7309E-16	3,08615E-13	ENSG00000127318	IL22
22,96744126	-2,47297472	0,547597435	-4,51604511	6,30052E-06	0,000819862	ENSG00000168811	IL12A
18,80214147	-2,49963631	0,576187504	-4,338234152	1,43632E-05	0,001603827	ENSG00000243836	WDR86-AS1
209,1673577	-2,524715787	0,302485241	-8,346575133	7,02709E-17	1,45162E-13	ENSG00000157570	TSPAN18
14,37087873	-2,598633142	0,643374213	-4,03906947	5,36637E-05	0,004518853	ENSG00000109956	B3GAT1
13,09590847	-2,720175126	0,749651298	-3,628587229	0,000284976	0,016819718	ENSG00000073756	PTGS2
9,307887484	-2,863450413	0,885387324	-3,234121763	0,001220174	0,04875966	ENSG00000251301	ENSG00000251301
40,21721324	-2,874339281	0,479936901	-5,988994126	2,11143E-09	8,94704E-07	ENSG00000138678	GPAT3
236,8783193	-2,880267508	0,253519137	-11,36114434	6,52838E-30	1,07888E-25	ENSG00000056736	IL17RB
16,31175126	-2,896375742	0,640777633	-4,520094951	6,18119E-06	0,000810717	ENSG00000066735	KIF26A
2632,364376	-2,923473778	0,267388434	-10,93343393	7,97714E-28	6,59151E-24	ENSG00000225783	MIAT
12,02255615	-2,926526242	0,761875965	-3,841210874	0,000122429	0,0087587	ENSG00000100292	HMOX1
199,6756305	-3,067641399	0,356220195	-8,611643705	7,20204E-18	1,7003E-14	ENSG00000169896	ITGAM
9,903145885	-3,203935385	0,944650665	-3,391661598	0,000694702	0,032801836	ENSG00000277693	ENSG00000277693
14,24182581	-3,508183428	0,739587192	-4,743434532	2,10125E-06	0,00032153	ENSG00000169429	CXCL8
60,71657136	-4,208421562	0,466267605	-9,025764424	1,78445E-19	4,91497E-16	ENSG00000156510	HKDC1

baseMean	log2FoldChange	lfcSE	stat	pvalue	padj	ENSEMBL	SYMBOL
42,10690893	1,766621692	0,558178465	3,164976442	0,001550955	0,049289469	ENSG00000119514	GALNT12
56,31125049	-1,537697839	0,485334562	-3,168325441	0,001533198	0,048909013	ENSG00000156510	HKDC1
85,19183722	-1,322279564	0,416944436	-3,171356778	0,001517287	0,048676975	ENSG00000273338	ENSG00000273338
49,01901219	-1,72559844	0,543536454	-3,174761194	0,001499599	0,048292788	ENSG00000153814	JAZF1
135,9908463	-1,385774497	0,435871625	-3,179317985	0,00147622	0,047904913	ENSG00000198756	COLGALT2
105,1859207	-1,199274457	0,37658464	-3,184608	0,001449502	0,04752033	ENSG00000274591	ENSG00000274591
48,15837758	1,961979942	0,615724335	3,18645834	0,001440262	0,047374542	ENSG00000112812	PRSS16
153,5863128	1,2514568	0,392417196	3,189097754	0,001427176	0,047035606	ENSG00000236552	RPL13AP5
18,96884816	2,650480305	0,830457472	3,191590653	0,001414917	0,04672266	ENSG00000164283	ESM1
44,51445629	1,88025097	0,583641761	3,22158402	0,001274841	0,043021423	ENSG00000164056	SPRY1
106,6350924	-1,303632926	0,404215166	-3,225096519	0,001259302	0,042703183	ENSG00000155629	PIK3AP1
28,2338041	-2,276561567	0,705430143	-3,227196328	0,001250097	0,04252592	ENSG00000163421	PROK2
148,3843419	1,12700835	0,34907949	3,228514942	0,001244348	0,042501383	ENSG00000114107	CEP70
233,0969496	1,044099172	0,323175378	3,230750993	0,001234654	0,042341387	ENSG00000107438	PDLIM1
99,36782456	1,536978177	0,475683687	3,231092886	0,001233179	0,042341387	ENSG00000138449	SLC40A1
135,0584419	-1,685246711	0,519014704	-3,247011493	0,001166237	0,04032222	ENSG00000184357	H1-5
138,13001	-1,269850121	0,390338438	-3,253202853	0,00114112	0,039651572	ENSG00000180611	MB21D2
253,6138419	-1,160807027	0,356847846	-3,252946711	0,001142149	0,039651572	ENSG00000121039	RDH10
24,20670755	2,526398225	0,775936087	3,255935981	0,001130192	0,039398271	ENSG00000166689	PLEKHA7
187,2436526	1,285785378	0,392304518	3,277518662	0,001047238	0,037118772	ENSG00000211785	TRAV12-1
120,7774068	-1,316016531	0,398560947	-3,301920426	0,000960253	0,034764451	ENSG00000177468	OLIG3
278,513808	1,099061212	0,332597787	3,304475417	0,000951543	0,034597292	ENSG00000186399	GOLGA8R
40,82223165	-2,920814642	0,883007481	-3,307802826	0,00094031	0,034310929	ENSG00000205809	KLRC2
46,98360716	1,833277842	0,554294507	3,30740756	0,000941638	0,034310929	ENSG00000178115	GOLGA8Q
228,8652534	1,032585392	0,311872125	3,310925569	0,000929879	0,03427446	ENSG00000147124	ZNF41
36,71482003	2,264531082	0,683133454	3,314917559	0,000916702	0,033988319	ENSG00000255036	SUGT1P4-STRA6LP-CCDC180
27,21820414	3,3288017	1,002996628	3,318856323	0,000903869	0,033809108	ENSG00000173947	CIMAP3
102,6458007	1,542067574	0,463768704	3,32507899	0,000883935	0,033210416	ENSG00000018408	WWTR1
280,370548	-1,271975662	0,380027571	-3,347061526	0,000816731	0,031194121	ENSG00000182240	BACE2
18,69514074	-2,957911504	0,880903692	-3,357814858	0,000785612	0,03036683	ENSG00000189143	CLDN4
25,72299389	2,523934215	0,751722887	3,357532752	0,000786414	0,03036683	ENSG00000155966	AFF2
127,0609481	-1,756823734	0,520701375	-3,373956395	0,000740961	0,028931705	ENSG00000000938	FGR
105,5938684	1,353477788	0,398710381	3,394638945	0,000687192	0,027401765	ENSG00000113319	RASGRF2
47,26882122	-2,452128128	0,720526656	-3,40324415	0,000665907	0,026678904	ENSG00000222328	ENSG00000222328
210,1358943	-1,375642732	0,404055188	-3,404591186	0,000662632	0,026610724	ENSG00000111537	IFNG
722,5443779	-2,688381509	0,78392711	-3,429376882	0,000604969	0,025169124	ENSG00000277632	CCL3
176,5574694	1,114798935	0,323735705	3,443546444	0,000574138	0,024328197	ENSG00000221926	TRIM16
250,9625581	-1,210857671	0,351478648	-3,445039058	0,000570977	0,02425504	ENSG00000206652	RNU1-1
359,9081929	1,082423597	0,313750732	3,449947639	0,000560695	0,023878275	ENSG00000133985	TTC9
283,7207669	-1,118724309	0,323474023	-3,458467233	0,000543258	0,02331185	ENSG00000124212	PTGIS
229,5934686	1,073516058	0,310209089	3,46062091	0,000538931	0,023209757	ENSG00000008300	CELSR3
32,73318603	2,904953127	0,839483095	3,46040694	0,00053936	0,023209757	ENSG00000187416	LHFPL3
762,9261156	1,148320185	0,331197298	3,467178603	0,000525952	0,02285932	ENSG00000117650	NEK2
188,4909356	1,095686032	0,315939236	3,468027734	0,000524293	0,02284594	ENSG00000166780	BMERB1
196,5575037	1,110675301	0,320009196	3,470760579	0,000518987	0,022731878	ENSG00000256988	ENSG00000256988
277,5449294	1,07449833	0,308817992	3,479390311	0,000502556	0,022285565	ENSG00000224429	LINC00539
153,0538037	1,46348974	0,419701775	3,486975344	0,000488516	0,021850123	ENSG00000053918	KCNQ1
200,4639095	-1,070013506	0,306637218	-3,489509573	0,000483908	0,0217014	ENSG00000224259	LINC01133
55,9706593	-1,714482892	0,490638637	-3,494390298	0,000475146	0,021664815	ENSG00000267452	LINC02072
180,2508516	1,378351989	0,394433942	3,494506539	0,000474939	0,021664815	ENSG00000119408	NEK6
38,59375677	2,311463656	0,661095104	3,496416236	0,000471553	0,021664815	ENSG00000182871	COL18A1
52,02167432	1,968907988	0,561460537	3,506761133	0,000453596	0,021068543	ENSG00000188385	JAKMIP3
102,7019853	1,402091294	0,399291014	3,511452161	0,000445666	0,020757216	ENSG00000174500	GCSAM
268,6009611	-1,030101651	0,293229614	-3,512952314	0,000443157	0,020697391	ENSG00000269028	ENSG00000269028
104,0079938	-2,134783197	0,606944434	-3,517262994	0,000436022	0,020446801	ENSG00000114554	PLXNA1
214,7391681	-1,556835174	0,441999056	-3,52225905	0,000427886	0,020207445	ENSG00000228049	POLR2J2
18,06724646	4,604615129	1,306410217	3,524631903	0,000424072	0,020089204	ENSG00000143341	HMCN1
20,52376033	3,205435313	0,90777361	3,531095505	0,000413842	0,019709385	ENSG00000137462	TLR2
65,04318631	-1,807515376	0,511196065	-3,535855418	0,000406457	0,019437461	ENSG00000152217	SETBP1
207,5047055	-1,113019989	0,31329893	-3,552581526	0,000381471	0,018533123	ENSG00000177337	ENSG00000177337
130,5451973	1,280520828	0,360122407	3,555793263	0,00037684	0,018360921	ENSG00000172828	CES3
61,25320681	1,700419095	0,47795289	3,55771276	0,000374098	0,018279988	ENSG00000038382	TRIO
207,3151306	1,392468502	0,390391896	3,566847865	0,000361301	0,017809089	ENSG00000135709	KIAA0513
28,84608058	-2,470123146	0,691152052	-3,573921451	0,000351674	0,017436247	ENSG00000169583	CLIC3
69,82018374	-1,701529876	0,475153807	-3,581008615	0,00034227	0,017070104	ENSG00000187240	DYNC2H1
55,48495921	1,900306898	0,530114932	3,584707357	0,000337456	0,016929896	ENSG00000196205	ENSG00000196205
286,6495202	1,10049702	0,306896241	3,585892796	0,000335927	0,016903324	ENSG00000240350	ENSG00000240350
203,6826983	-1,181295266	0,329295767	-3,587338147	0,000334071	0,016889929	ENSG00000049759	NEDD4L
64,3464902	1,812186538	0,505226804	3,586877266	0,000334662	0,016889929	ENSG00000109265	CRACD
99,65156127	-1,536670715	0,425945382	-3,607670798	0,000308958	0,015781134	ENSG00000115607	IL18RAP
117,7600727	-1,335176923	0,369193962	-3,616464681	0,000298654	0,015455419	ENSG00000284634	ENSG00000284634
52,00955656	2,078113912	0,574126512	3,619609738	0,000295048	0,015396206	ENSG00000186265	BTLA
40,44094042	-2,057091689	0,567944159	-3,62199638	0,000292338	0,01530205	ENSG00000116711	PLA2G4A
283,6113674	1,21207605	0,33461982	3,622248228	0,000292054	0,01530205	ENSG00000064886	CHI3L2

390,6153107	1,179529902	0,325316843	3,625787988	0,000288082	0,015173197	ENSG00000118513	MYB
22,65911769	2,831499725	0,778637737	3,63647893	0,00027639	0,014694748	ENSG00000103888	CEMIP
333,4196998	1,1557746	0,31468936	3,672747625	0,000239956	0,012961481	ENSG00000285565	ENSG00000285565
133,2077034	-1,439197608	0,391185006	-3,679071499	0,000234085	0,012802607	ENSG00000196083	IL1RAP
282,0353553	1,054890185	0,286894104	3,676932254	0,000236056	0,012802607	ENSG00000106991	ENG
273,7443378	1,13334615	0,308226975	3,6769856	0,000236006	0,012802607	ENSG00000115155	OTOF
35,62475084	2,403025903	0,653579829	3,676713688	0,000236258	0,012802607	ENSG00000273540	AGBL1
79,97504573	-1,645588322	0,447011781	-3,681308622	0,00023204	0,012778824	ENSG00000217825	ENSG00000217825
27,88889648	-3,355519451	0,911097046	-3,682944059	0,000230556	0,012760497	ENSG00000100427	MLC1
256,5805358	-1,132764207	0,307011691	-3,689645183	0,000224567	0,012530544	ENSG00000143842	SOX13
51,68650294	-1,919521611	0,519925777	-3,691914686	0,000222572	0,012460354	ENSG00000232022	ENSG00000232022
55,01157277	-2,099217914	0,568319073	-3,693731237	0,000220987	0,012412736	ENSG00000283554	LINC02341
30,47395085	-2,883025412	0,778212712	-3,704675299	0,000211662	0,011928547	ENSG00000127318	IL22
116,0985172	-1,448396603	0,389220092	-3,721279127	0,000198216	0,011245776	ENSG00000238741	SCARNA7
33,11165484	3,021040983	0,808979011	3,734387345	0,000188173	0,010784528	ENSG00000260784	ENSG00000260784
20,26197923	3,609531598	0,966242504	3,73563736	0,00018724	0,010767593	ENSG00000171914	TLN2
4658,301778	1,002994148	0,267749985	3,746010104	0,000179669	0,010510968	ENSG00000117724	CENPF
78,05079125	-1,62401871	0,432475625	-3,755168188	0,000173225	0,010312384	ENSG00000266964	FXYD1
44,80861812	-2,222013627	0,590747428	-3,7613598	0,000168992	0,010131738	ENSG00000196139	AKR1C3
988,039341	1,030968001	0,273409804	3,770779204	0,000162739	0,009859663	ENSG00000156970	BUB1B
1214,894517	1,095464458	0,290578862	3,769938567	0,000163288	0,009859663	ENSG00000131242	RAB11FIP4
203,8928337	-1,538270542	0,407396072	-3,77586002	0,000159457	0,009732605	ENSG00000230562	ENSG00000230562
532,3020725	1,208546906	0,319529511	3,782270073	0,000155405	0,009554276	ENSG00000183963	SMTN
25,82751505	-3,047233377	0,800002768	-3,80902854	0,000139514	0,008700563	ENSG00000196422	PPP1R26
508,7419695	1,002434066	0,262880087	3,813275012	0,000137137	0,008619262	ENSG00000112679	DUSP22
44,67969643	-2,897065388	0,757653173	-3,823735569	0,000131445	0,008395757	ENSG00000004399	PLXND1
626,6851966	1,192229923	0,311895827	3,822526041	0,000132092	0,008395757	ENSG00000126353	CCR7
74,08113889	1,903923558	0,497091724	3,830125238	0,000128078	0,008264945	ENSG00000280135	ENSG00000280135
115,4073723	-1,490207184	0,388731808	-3,833509772	0,000126328	0,008183233	ENSG00000081181	ARG2
230,5577132	1,239994563	0,323024233	3,838704459	0,000123685	0,008042868	ENSG00000136295	TTYH3
64,67593711	-1,859420022	0,484178651	-3,840359375	0,000122854	0,008019684	ENSG00000180263	FGD6
2770,96015	1,018260567	0,263320542	3,86700011	0,000110182	0,007334069	ENSG00000271383	NBPF19
339,9661139	1,023421898	0,264545806	3,868599971	0,000109462	0,007314921	ENSG00000280893	ENSG00000280893
131,0915536	1,364896983	0,352737473	3,86944141	0,000109085	0,007314921	ENSG00000083454	P2RX5
3035,192427	-1,13078044	0,290741286	-3,889301223	0,000100533	0,006826169	ENSG00000090104	RGS1
657,7207801	1,051221653	0,270173266	3,890916633	9,98662E-05	0,00680822	ENSG00000120896	SORBS3
272,0220799	-1,467912826	0,376626825	-3,897525951	9,71804E-05	0,00665194	ENSG00000049249	TNFRSF9
988,7516523	1,144382744	0,292988705	3,905893729	9,38778E-05	0,006451997	ENSG00000171316	CHD7
257,3041018	-1,231891548	0,313523066	-3,929189524	8,52326E-05	0,005930158	ENSG00000115604	IL18R1
420,0617848	1,029093878	0,261603673	3,93378986	8,36169E-05	0,00586602	ENSG00000130766	SESN2
565,640631	1,047346337	0,264983295	3,95249949	7,73391E-05	0,005517182	ENSG00000234664	ENSG00000234664
60,72703079	1,925832985	0,486563799	3,958027684	7,55712E-05	0,005413908	ENSG00000280987	MATR3
185,4244407	1,308587022	0,33043886	3,960148698	7,49031E-05	0,005400945	ENSG00000121742	GJB6
124,737879	1,4562855	0,366441245	3,974130968	7,06367E-05	0,005147649	ENSG00000274422	ENSG00000274422
1521,387559	-1,265031705	0,317362095	-3,986083172	6,7173E-05	0,00495936	ENSG00000184557	SOCS3
325,792391	-1,43573014	0,359911618	-3,989118633	6,63192E-05	0,004939469	ENSG00000211829	ENSG00000211829
195,590455	1,550041226	0,388293753	3,991929341	6,55379E-05	0,004902873	ENSG00000269897	COMMD3-BMI1
83,78910456	2,756980405	0,682656033	4,038608421	5,37692E-05	0,004113468	ENSG00000101134	DOK5
120,7717549	-1,614754296	0,398260318	-4,054519675	5,02374E-05	0,003878375	ENSG00000176485	PLAAT3
82,76658048	-1,793518479	0,440909253	-4,067772376	4,74647E-05	0,003681128	ENSG00000115657	ABCB6
363,9963122	1,232739528	0,302983354	4,068670806	4,72821E-05	0,003681128	ENSG00000066923	STAG3
98,45348849	2,388966908	0,586928618	4,07028527	4,69556E-05	0,003675363	ENSG00000184602	SNN
491,2312525	1,087783498	0,266656629	4,079341668	4,51634E-05	0,003551525	ENSG00000072110	ACTN1
602,6446005	1,120995143	0,274675051	4,081168414	4,48099E-05	0,003551525	ENSG00000127947	PTPN12
319,2915606	1,262798929	0,309469	4,080534489	4,49323E-05	0,003551525	ENSG00000163814	CDCP1
132,5049956	2,071979312	0,50787326	4,079717278	4,50905E-05	0,003551525	ENSG00000158457	TSPAN33
481,1687755	1,01523449	0,248344884	4,088002436	4,35104E-05	0,003486396	ENSG00000137824	RMDN3
21,27702026	-3,703933638	0,904814518	-4,093583339	4,24757E-05	0,003426117	ENSG00000226928	ENSG00000226928
291,2609398	1,165830908	0,284824945	4,093148888	4,25554E-05	0,003426117	ENSG00000198520	ARMH1
637,0336167	1,089159529	0,265494565	4,102379758	4,08922E-05	0,003339928	ENSG00000189060	H1-0
403,0072737	-1,056318547	0,257219264	-4,106685219	4,01378E-05	0,003310289	ENSG00000124588	NQO2
44,39179784	3,05166663	0,742862739	4,107981825	3,99132E-05	0,0033079	ENSG00000258830	ENSG00000258830
669,7475554	1,057169598	0,255537486	4,137043121	3,5181E-05	0,002974026	ENSG00000152223	EPG5
229,9044687	1,630287638	0,393443909	4,143634204	3,41845E-05	0,002918975	ENSG00000113749	HRH2
51,3068531	-2,350551249	0,566419112	-4,149844522	3,32701E-05	0,002869887	ENSG00000287061	ENSG00000287061
746,0597344	1,128462043	0,271885115	4,150510564	3,31734E-05	0,002869887	ENSG00000103381	CPPED1
69,94041494	2,479945234	0,597394842	4,151266568	3,3064E-05	0,002869887	ENSG00000144785	ENSG00000144785
117,4201206	1,826699956	0,439708502	4,154343044	3,26223E-05	0,002857751	ENSG00000150054	MPP7
1854,652946	1,015444523	0,243659291	4,167477122	3,07989E-05	0,002712071	ENSG00000172794	RAB37
5317,401888	1,016742341	0,243394678	4,177340073	2,94938E-05	0,002610741	ENSG00000143401	ANP32E
245,9883686	-1,468285738	0,351320091	-4,179338939	2,92358E-05	0,002601522	ENSG00000164120	HPGD
358,454178	1,205368874	0,28794945	4,186043324	2,83859E-05	0,00255277	ENSG00000104081	BMF
37,85729728	-3,380638241	0,80571775	-4,195809561	2,71899E-05	0,002458284	ENSG00000197057	DTHD1
480,523254	-1,150323976	0,272892525	-4,215300419	2,49446E-05	0,002292055	ENSG00000178996	SNX18
33,09334825	3,792528182	0,898393928	4,22145349	2,42732E-05	0,002254874	ENSG00000164932	CTHRC1

177,8637539	2,093518247	0,494569173	4,233014029	2,3058E-05	0,002177887	ENSG00000197408	CYP2B6
76,40471647	2,237826447	0,527898644	4,239121417	2,24396E-05	0,002143428	ENSG00000089199	CHGB
698,0190864	1,065227803	0,249497779	4,269488117	1,95922E-05	0,001882077	ENSG00000167220	HDHD2
183,9172734	-1,377463526	0,322491857	-4,271312588	1,94326E-05	0,001877409	ENSG00000171621	SPSB1
1087,179319	1,06049583	0,248260278	4,271709678	1,9398E-05	0,001877409	ENSG00000006715	VPS41
351,9129926	-1,19944211	0,279784104	-4,287027372	1,8108E-05	0,001790362	ENSG00000174792	ODAPH
1107,752808	1,245862751	0,290529056	4,288255259	1,80082E-05	0,001790362	ENSG00000166326	TRIM44
302,3195984	1,597676679	0,372641501	4,287436245	1,80747E-05	0,001790362	ENSG00000134256	CD101
115,5938352	-1,840162727	0,428260288	-4,29683251	1,73256E-05	0,001746516	ENSG00000198848	CES1
1719,026666	-1,060357611	0,246414687	-4,3031429	1,68392E-05	0,001715064	ENSG00000010404	IDS
238,5440168	1,643457228	0,38179512	4,304552731	1,67323E-05	0,001714507	ENSG00000137331	IER3
732,511088	-1,21669874	0,28198203	-4,314809486	1,59741E-05	0,001646789	ENSG00000069702	TGFBR3
568,7216454	1,144573287	0,264718588	4,323735989	1,53409E-05	0,001591217	ENSG00000159496	RGL4
1892,609947	-1,400146142	0,323562394	-4,327283297	1,5096E-05	0,001579095	ENSG00000172543	CTSW
253,4706154	1,381197641	0,319220814	4,326778146	1,51306E-05	0,001579095	ENSG00000204104	TRAF3IP1
812,6800109	1,103909796	0,25447581	4,337975363	1,43801E-05	0,00151953	ENSG00000130787	HIP1R
305,9262257	1,195540237	0,275462219	4,340124178	1,42402E-05	0,00151421	ENSG00000148384	INPP5E
22,69743377	4,698898338	1,078533798	4,35674649	1,3201E-05	0,001412591	ENSG00000263647	BPTFP1
247,1029679	-1,450844483	0,330491204	-4,389963985	1,13369E-05	0,001220852	ENSG00000205002	AARD
54,8866102	-2,415492414	0,54989514	-4,392641868	1,11982E-05	0,001213636	ENSG00000100292	HMOX1
115,8943692	1,768361179	0,402180669	4,396932307	1,09792E-05	0,001197578	ENSG00000124191	TOX2
1869,173821	-1,002701962	0,227597013	-4,405602462	1,0549E-05	0,001158132	ENSG00000143013	LMO4
1425,866408	1,119548852	0,254020788	4,407311934	1,04661E-05	0,001156543	ENSG00000103365	GGA2
5181,431396	1,124721033	0,255161974	4,407870873	1,04392E-05	0,001156543	ENSG00000150593	PDCD4
67,52978299	-2,046909119	0,462522547	-4,425533705	9,62041E-06	0,001077168	ENSG00000079156	OSBPL6
852,9524927	1,01548811	0,228616188	4,441890656	8,91719E-06	0,001010444	ENSG00000181827	RFX7
242,0384771	1,32944248	0,299373637	4,440746672	8,96473E-06	0,001010444	ENSG00000205476	CCDC85C
882,3472869	1,015134055	0,226167534	4,488416344	7,17546E-06	0,000825275	ENSG00000164402	SEPTIN8
25,14360921	-4,78401979	1,064126702	-4,495723845	6,93337E-06	0,000802893	ENSG00000253230	MIR124-1HG
1909,421588	-1,324951884	0,293667371	-4,511743606	6,42969E-06	0,000749702	ENSG00000136810	TXN
266,9585911	1,333072209	0,295457585	4,511890284	6,42524E-06	0,000749702	ENSG00000141753	IGFBP4
93,18962711	2,112045485	0,467681929	4,515986942	6,30225E-06	0,00074512	ENSG00000164512	ANKRD55
246,3870503	1,631425517	0,361093439	4,518014846	6,24221E-06	0,000743219	ENSG00000154358	OBSCN
47,06021121	-2,448548843	0,53887412	-4,54382341	5,52429E-06	0,000662406	ENSG00000168994	PXDC1
6891,684546	1,118660564	0,245851955	4,550138974	5,36105E-06	0,000647423	ENSG00000167106	EEIG1
367,3744686	1,265674101	0,277951874	4,553572825	5,27424E-06	0,000641522	ENSG00000136040	PLXNC1
718,1288156	-1,156571933	0,253029465	-4,570898236	4,85638E-06	0,000596849	ENSG00000160791	CCR5
804,0805829	1,055132592	0,230870274	4,57024014	4,87166E-06	0,000596849	ENSG00000119139	TJP2
150,7201942	1,879968697	0,40964427	4,589271318	4,44796E-06	0,000552953	ENSG00000171777	RASGRP4
1352,459974	1,169387156	0,253815322	4,607236256	4,08056E-06	0,000511038	ENSG00000136108	CKAP2
177,4230202	-1,578467753	0,342013889	-4,615215355	3,92688E-06	0,000496411	ENSG00000188322	SBK1
202,7576103	-1,571512505	0,340536236	-4,61481727	3,93441E-06	0,000496411	ENSG00000111186	WNT5B
770,5292558	-1,629760299	0,352794011	-4,619580405	3,84517E-06	0,000492502	ENSG00000134539	KLRD1
668,7616886	1,104143496	0,238572695	4,628121826	3,68997E-06	0,000477551	ENSG00000118308	IRAG2
367,5298545	1,199759158	0,259264526	4,627548458	3,7002E-06	0,000477551	ENSG00000173208	ABCD2
235,9903897	1,641205188	0,354424571	4,630619098	3,64574E-06	0,000477551	ENSG00000128271	ADORA2A
95,15307419	2,523546971	0,545177733	4,628851877	3,67699E-06	0,000477551	ENSG00000134909	ARHGAP32
516,9563524	1,361553964	0,291081651	4,677567133	2,90299E-06	0,000386463	ENSG00000153317	ASAP1
506,8741008	1,277657838	0,272762783	4,68413551	2,81144E-06	0,000377246	ENSG00000137265	IRF4
1588,588816	1,279445632	0,272408435	4,696791542	2,6428E-06	0,000357454	ENSG00000184730	APOBR
235,2011055	-1,411257926	0,299686203	-4,709118775	2,4879E-06	0,000339217	ENSG00000139112	GABARAPL1
416,7403988	-1,561508312	0,331443118	-4,711240709	2,46213E-06	0,000338433	ENSG00000200312	ENSG00000200312
115,5339318	-1,901020263	0,40184309	-4,730752648	2,23689E-06	0,000309993	ENSG00000180549	FUT7
225,2753306	-1,627773148	0,343452106	-4,739447281	2,14302E-06	0,000299438	ENSG00000013619	MAMLD1
469,3979426	-1,568953233	0,330743427	-4,743717046	2,09832E-06	0,000295636	ENSG00000113088	GZMK
1474,830549	1,030290384	0,217085911	4,746002997	2,07476E-06	0,000294772	ENSG00000144354	CDCA7
357,5739952	1,672679985	0,348894214	4,794232519	1,63299E-06	0,000233974	ENSG00000157483	MYO1E
2769,841565	-1,428623033	0,29763089	-4,799982404	1,5868E-06	0,000229299	ENSG00000202198	RN7SK
43,61147676	-3,244867136	0,674960081	-4,807494885	1,52833E-06	0,000222755	ENSG00000108924	HLF
817,2625188	-1,25918159	0,261817566	-4,809385447	1,51395E-06	0,000222577	ENSG00000133816	MICAL2
254,4986254	1,955620965	0,406341079	4,812757231	1,48862E-06	0,000220773	ENSG00000141469	SLC14A1
1438,356876	1,290777942	0,267770983	4,820454876	1,43231E-06	0,000214302	ENSG00000142102	PGGHG
1353,62226	-1,043877661	0,216464698	-4,822392154	1,41847E-06	0,000214125	ENSG00000172005	MAL
689,8028282	1,314698351	0,272467008	4,825165291	1,39887E-06	0,00021307	ENSG00000134352	IL6ST
207,3580632	1,838548883	0,379747874	4,841498813	1,28863E-06	0,000198063	ENSG00000138119	MYOF
53,30768581	2,816338411	0,580870564	4,848478453	1,24412E-06	0,000192976	ENSG00000150681	RGS18
722,5969984	1,220013201	0,249788784	4,884179278	1,03861E-06	0,00016259	ENSG00000106415	GLCCI1
221,9205588	-1,545815371	0,316317951	-4,886903721	1,02434E-06	0,000161856	ENSG00000155657	TTN
784,0605026	1,119891097	0,228927852	4,891895367	9,98695E-07	0,000159292	ENSG00000109171	SLAIN2
35,12422779	-3,218339849	0,649679697	-4,953733147	7,2803E-07	0,000117227	ENSG00000087842	PIR
644,1893265	1,165749938	0,235039524	4,95980386	7,05644E-07	0,000114715	ENSG00000085719	CPNE3
1564,279246	1,147920408	0,230791322	4,973845632	6,56376E-07	0,000107741	ENSG00000198826	ARHGAP11A
5780,518718	1,083036235	0,217068538	4,989374522	6,05751E-07	0,000100406	ENSG00000115306	SPTBN1
12028,44123	1,00553774	0,199092487	5,050606163	4,4041E-07	7,37229E-05	ENSG00000125354	SEPTIN6
143,9093559	-2,663921549	0,526121387	-5,063321151	4,12015E-07	6,96594E-05	ENSG00000164530	PI16

229,2100569	-1,730540172	0,341032718	-5,074410987	3,88698E-07	6,6381E-05	ENSG00000189430	NCR1
1661,868819	1,261217866	0,24829354	5,07954361	3,78343E-07	6,52718E-05	ENSG00000129250	KIF1C
138,854804	1,945493006	0,382047399	5,092281778	3,5378E-07	6,16635E-05	ENSG00000196814	MVB12B
69,72693333	-2,36741053	0,464482141	-5,096881707	3,45294E-07	6,08113E-05	ENSG00000177301	KCNA2
119,2671693	-2,712264287	0,526911164	-5,147479254	2,6401E-07	4,69855E-05	ENSG00000203747	FCGR3A
935,9483004	1,256198169	0,243670995	5,155304475	2,53219E-07	4,55444E-05	ENSG00000149212	SESN3
1088,257131	-1,410529857	0,270230838	-5,219722017	1,79192E-07	3,25763E-05	ENSG00000033327	GAB2
565,8095462	1,618885031	0,309779485	5,225927187	1,73285E-07	3,19765E-05	ENSG00000004468	CD38
26,36437896	5,734113436	1,09740365	5,225163445	1,74001E-07	3,19765E-05	ENSG00000250548	LINC01303
253,5448879	-1,808562458	0,345384158	-5,236379312	1,63757E-07	3,07627E-05	ENSG00000169896	ITGAM
41,80069812	-3,39509784	0,6469919	-5,247512129	1,54167E-07	2,92865E-05	ENSG00000081189	MEF2C
121,5180149	2,025504412	0,384178437	5,272301135	1,34724E-07	2,58838E-05	ENSG00000266208	ENSG00000266208
660,8223629	-1,734134232	0,326817589	-5,306122713	1,11982E-07	2,17618E-05	ENSG00000168298	H1-4
1400,294975	1,179731072	0,218013876	5,411265978	6,25807E-08	1,23029E-05	ENSG00000100628	ASB2
401,8347249	1,424215363	0,262541885	5,42471675	5,80465E-08	1,15458E-05	ENSG00000106723	SPIN1
172,3487418	-1,779731933	0,327729053	-5,430497891	5,6197E-08	1,1311E-05	ENSG00000114757	PEX5L
1101,702951	1,720476851	0,316558321	5,434944332	5,48135E-08	1,11655E-05	ENSG00000138795	LEF1
335,7377749	1,714219281	0,312059844	5,493238913	3,94628E-08	8,13656E-06	ENSG00000179934	CCR8
417,1584389	2,007472019	0,36519052	5,497054023	3,86188E-08	8,06085E-06	ENSG00000234857	HNRNPUL2-BSCL2
150,177025	-1,945932181	0,353408366	-5,506185959	3,66691E-08	7,74956E-06	ENSG00000248323	LUCAT1
170,6537015	-1,998214128	0,359452433	-5,559050221	2,71247E-08	5,83413E-06	ENSG00000259001	ENSG00000259001
4708,31755	-1,141780696	0,20542359	-5,558177102	2,72607E-08	5,83413E-06	ENSG00000059804	SLC2A3
32,77527808	5,511457546	0,986709545	5,585693959	2,32769E-08	5,11094E-06	ENSG00000131142	CCL25
71,9458765	2,724432265	0,485392182	5,612847433	1,99024E-08	4,4275E-06	ENSG00000152670	DDX4
21,78509216	7,862438423	1,399519617	5,617955139	1,93231E-08	4,35593E-06	ENSG00000284829	ENSG00000284829
201,1486363	-1,782620576	0,316823931	-5,626533856	1,83867E-08	4,20086E-06	ENSG00000157570	TSPAN18
494,7273231	-1,43075316	0,253176528	-5,651207755	1,59324E-08	3,69E-06	ENSG00000146859	TMEM140
94,54945859	-2,731816224	0,482347723	-5,663582711	1,48245E-08	3,48107E-06	ENSG00000137441	FGFBP2
152,0177237	2,211852026	0,390211431	5,668342469	1,44186E-08	3,43344E-06	ENSG00000173801	JUP
98,66745283	-2,782503544	0,490271558	-5,675433341	1,38338E-08	3,34126E-06	ENSG00000120659	TNFSF11
301,5761769	1,608831601	0,280564829	5,734259728	9,79392E-09	2,39979E-06	ENSG00000151136	ABTB3
85,26074223	-2,880315027	0,501647681	-5,74170904	9,37258E-09	2,33033E-06	ENSG00000276070	CCL4L2
632,951699	1,581895283	0,275465002	5,742636165	9,32139E-09	2,33033E-06	ENSG00000228168	HNRNPA1P21
5193,230461	-1,273475201	0,220890506	-5,765187585	8,15671E-09	2,08948E-06	ENSG00000140564	FURIN
366,761458	1,62937736	0,28254024	5,766886022	8,07496E-09	2,08948E-06	ENSG00000146285	SCML4
141,9920778	-2,006872416	0,346257908	-5,795889041	6,79602E-09	1,79532E-06	ENSG00000184293	ENSG00000184293
456,4243955	1,530413186	0,261201912	5,859119396	4,65328E-09	1,24878E-06	ENSG00000131697	NPHP4
84,67215757	-2,873498592	0,487548	-5,89377578	3,77469E-09	1,03525E-06	ENSG00000090382	LYZ
359,0300605	-1,586962508	0,26908741	-5,897572493	3,68888E-09	1,03525E-06	ENSG00000121064	SCPEP1
649,745211	1,988239044	0,337399729	5,892829409	3,79638E-09	1,03525E-06	ENSG00000145287	PLAC8
360,7992974	-1,64902695	0,277541885	-5,941542654	2,82352E-09	8,09107E-07	ENSG00000107281	NPDC1
722,5968356	-1,571401926	0,258653315	-6,075321043	1,2374E-09	3,60702E-07	ENSG00000171476	HOPX
37,29474033	-5,404966934	0,884834175	-6,108451827	1,00602E-09	2,984E-07	ENSG00000196782	MAML3
470,5156967	-1,760029543	0,284755385	-6,180847266	6,37585E-10	1,92494E-07	ENSG00000235831	BHLHE40-AS1
1244,903018	-2,150987244	0,347790489	-6,184721301	6,22123E-10	1,91241E-07	ENSG00000139289	PHLDA1
6487,560897	1,910533519	0,308163054	6,199748779	5,65534E-10	1,77064E-07	ENSG00000081059	TCF7
130,5257104	-3,139291322	0,504823416	-6,21859292	5,01633E-10	1,60021E-07	ENSG00000177508	IRX3
255,9683899	-2,156642431	0,343802325	-6,272914034	3,54353E-10	1,15212E-07	ENSG00000163106	HPGDS
732,1053229	1,714281145	0,273265642	6,273313892	3,53443E-10	1,15212E-07	ENSG00000152229	PSTPIP2
269,8526606	-2,414432196	0,383416878	-6,297146359	3,03175E-10	1,02516E-07	ENSG00000107968	MAP3K8
351,6690275	-1,81039005	0,286189173	-6,325850937	2,51841E-10	8,68954E-08	ENSG00000167851	CD300A
86,47044927	-2,743322638	0,429905772	-6,381218429	1,75685E-10	6,18812E-08	ENSG00000171954	CYP4F22
101,3166725	3,086701376	0,482427707	6,398267203	1,5715E-10	5,65306E-08	ENSG00000129226	CD68
206,7189641	2,206279888	0,344244472	6,409049587	1,4643E-10	5,38192E-08	ENSG00000255026	ENSG00000255026
586,8343345	1,713143243	0,266498683	6,428336631	1,29008E-10	4,84697E-08	ENSG00000232653	GOLGA8N
169,1335264	-2,208187658	0,340018603	-6,494314246	8,34125E-11	3,20512E-08	ENSG00000178860	MSC
2571,16348	-2,004096257	0,304987383	-6,571079233	4,99519E-11	1,96404E-08	ENSG00000105374	NKG7
414,8247484	-1,917472392	0,29008411	-6,610056625	3,84173E-11	1,54648E-08	ENSG00000126822	PLEKHG3
532,3102739	1,865707096	0,282207575	6,611116286	3,81433E-11	1,54648E-08	ENSG00000110841	PPFIBP1
2147,057304	1,503875777	0,222534754	6,757936679	1,39971E-11	5,91621E-09	ENSG00000137312	FLOT1
386,1921476	1,758906621	0,259545445	6,776873389	1,22804E-11	5,32373E-09	ENSG00000177374	HIC1
85,4527136	-3,444708381	0,50769199	-6,785035899	1,16058E-11	5,16365E-09	ENSG00000111536	IL26
242,9566174	-2,2385652	0,329066656	-6,802771284	1,02626E-11	4,68943E-09	ENSG00000186891	TNFRSF18
589,2763775	-2,503353658	0,365283989	-6,853171051	7,22306E-12	3,39223E-09	ENSG00000275302	CCL4
1144,588789	-1,645412351	0,238608336	-6,895871187	5,35357E-12	2,58608E-09	ENSG00000280800	ENSG00000280800
366,9846105	-2,091130323	0,296245362	-7,058778265	1,67973E-12	8,3527E-10	ENSG00000172215	CXCR6
623,0514486	1,983338834	0,27904335	7,107636981	1,18047E-12	6,04792E-10	ENSG00000077782	FGFR1
228,7688076	2,317041861	0,309576539	7,484552515	7,17913E-14	3,79305E-11	ENSG00000116574	RHOU
5350,047252	-2,11973307	0,282896767	-7,49295614	6,73394E-14	3,6726E-11	ENSG00000225783	MIAT
96,21809033	-3,925073856	0,517902845	-7,578784121	3,48808E-14	1,96577E-11	ENSG00000236481	LINC02195
582,1233193	-2,087418671	0,273901252	-7,621062891	2,51595E-14	1,4668E-11	ENSG00000163508	EOMES
7679,690715	-1,874776062	0,24469277	-7,661755046	1,83409E-14	1,10746E-11	ENSG00000274012	RN7SL2
7442,65403	-2,033249984	0,257535997	-7,895012755	2,90286E-15	1,81773E-12	ENSG00000276168	RN7SL1
230,0212331	2,66915553	0,337155622	7,916687007	2,43923E-15	1,58615E-12	ENSG00000070214	SLC44A1
607,4300004	1,948693272	0,242998954	8,019348382	1,06308E-15	7,18937E-13	ENSG00000112303	VNN2

1180,443984	-1,981927712	0,238477247	-8,310762309	9,50892E-17	6,69864E-14	ENSG00000172965	MIR4435-2HG
188,7740081	3,208259712	0,385070266	8,331621511	7,97425E-17	5,86177E-14	ENSG00000131016	AKAP12
178,3904367	2,817755807	0,334233266	8,430506765	3,44171E-17	2,64496E-14	ENSG00000005102	MEOX1
144,3503383	-3,034228512	0,358229349	-8,470072364	2,45241E-17	2,07038E-14	ENSG00000175779	LINC02694
4275,123991	-2,525203946	0,298327267	-8,464542886	2,57159E-17	2,07038E-14	ENSG00000100453	GZMB
2558,061645	-1,893053212	0,220728153	-8,576401267	9,78876E-18	8,71045E-15	ENSG00000077984	CST7
1129,228599	-2,093198507	0,243298954	-8,603401185	7,73879E-18	7,43225E-15	ENSG00000222041	CYTOR
221,9885195	2,860678999	0,332604191	8,600850741	7,91273E-18	7,43225E-15	ENSG00000133026	MYH10
1516,576117	-2,172955672	0,249789023	-8,699163988	3,34338E-18	3,53291E-15	ENSG00000282885	ENSG00000282885
470,8812287	-2,490507832	0,280466358	-8,879880802	6,6932E-19	7,54413E-16	ENSG00000113525	IL5
141,8038371	-3,905695074	0,427813497	-9,129433977	6,88584E-20	8,31563E-17	ENSG00000116667	C1orf21
918,7778703	-3,155404893	0,333068141	-9,473751784	2,69969E-21	3,51105E-18	ENSG00000169194	IL13
261,1566841	-3,125763664	0,32552172	-9,602319819	7,8165E-22	1,10128E-18	ENSG00000100450	GZMH
343,3736583	-2,775285747	0,287165157	-9,6644237	4,27016E-22	6,56324E-19	ENSG00000115956	PLEK
184,497159	-4,893457741	0,498007446	-9,826073437	8,69428E-23	1,46994E-19	ENSG00000138678	GPAT3
696,3438061	-2,448347483	0,24641709	-9,935786027	2,90873E-23	5,46421E-20	ENSG00000136161	RCBTB2
3863,496235	-2,071752222	0,208002845	-9,96021098	2,27579E-23	4,8096E-20	ENSG00000132965	ALOX5AP
438,2147369	-3,643466036	0,343659058	-10,60197877	2,91741E-26	7,04638E-23	ENSG00000205336	ADGRG1
988,985833	-2,478475811	0,225423696	-10,9947439	4,05062E-28	1,1414E-24	ENSG00000016391	CHDH
1189,552284	-2,624907399	0,225234181	-11,65412544	2,18613E-31	7,39219E-28	ENSG00000139679	LPAR6
253,8962399	-3,845317312	0,325777966	-11,80349108	3,74448E-32	1,5827E-28	ENSG00000073756	PTGS2
16787,49505	-2,790565533	0,210858634	-13,23429581	5,56155E-40	3,13431E-36	ENSG00000115523	GNLY
1126,192212	-4,464096168	0,243393996	-18,34102831	3,89362E-75	3,29147E-71	ENSG00000056736	IL17RB
7150,295401	6,600984496	0,315409651	20,92828954	2,95926E-97	5,00322E-93	ENSG00000112182	BACH2

baseMean	log2FoldChange	lfcSE	stat	pvalue	padj	ENSEMBL	SYMBOL
6,821668232	-29,99494959	3,965159363	-7,5646265	3,88981E-14	7,93132E-11	ENSG00000260966	ENSG00000260966
27,15146104	-29,10442725	3,908427719	-7,446581937	9,57896E-14	1,59092E-10	ENSG00000124208	PEDS1-UBE2V1
26,18444987	-28,55442203	3,907990036	-7,306677287	2,73829E-13	4,11591E-10	ENSG00000278996	ENSG00000278996
10,98906546	-26,75200464	3,911162846	-6,839910711	7,92426E-12	7,83397E-09	ENSG00000261499	ENSG00000261499
2,276037345	-25,68714647	2,946268326	-8,718536004	2,81822E-18	9,19416E-15	ENSG00000239945	ENSG00000239945
3,854614013	-25,16556199	2,547630456	-9,878026827	5,18437E-23	2,81891E-19	ENSG00000257921	ENSG00000257921
0,929991374	-23,74923453	3,989398625	-5,953086356	2,63132E-09	1,53293E-06	ENSG00000131864	USP29
1,048146595	-23,59588053	3,80506959	-6,20116925	5,60452E-10	3,97482E-07	ENSG00000104369	JPH1
3,976276363	-23,58220728	2,547605417	-9,256616871	2,11012E-20	7,64896E-17	ENSG00000234769	ENSG00000234769
1,083258448	-23,51081387	3,803622637	-6,181163621	6,36308E-10	4,32477E-07	ENSG00000138650	PCDH10
2,690033816	-23,3539816	2,811864615	-8,305514241	9,93881E-17	2,94767E-13	ENSG00000267827	ENSG00000267827
1,627484905	-23,05601744	3,312015674	-6,961324979	3,37087E-12	3,92755E-09	ENSG00000254959	INMT-MINDY4
1,112265938	-23,01130543	3,786084119	-6,077864282	1,21794E-09	7,49698E-07	ENSG00000230646	KLF2P2
0,802106307	-22,89282494	4,012195142	-5,705810443	1,15791E-08	5,39651E-06	ENSG00000233087	RAB6D
0,894713277	-22,6911314	3,988919458	-5,688540879	1,28129E-08	5,88746E-06	ENSG00000229111	MED4-AS1
0,917865019	-22,5872205	3,989834612	-5,661192179	1,50325E-08	6,48594E-06	ENSG00000265630	ENSG00000265630
0,833886928	-22,51116886	3,998047002	-5,630541325	1,79645E-08	7,51376E-06	ENSG00000070731	ST6GALNAC2
0,877170861	-22,47051443	3,986186432	-5,63709571	1,72942E-08	7,32735E-06	ENSG00000251660	ENSG00000251660
2,324940642	-20,68559993	2,732404609	-7,570474687	3,71863E-14	7,93132E-11	ENSG00000258830	ENSG00000258830
0,742249989	-19,35893544	3,974855595	-4,870349369	1,11401E-06	0,000306504	ENSG00000225756	DBH-AS1
0,741356787	-19,20372261	3,998388136	-4,802866044	1,56411E-06	0,000384517	ENSG00000225721	ENSG00000225721
4,636993713	-19,1323563	3,968356652	-4,821229033	1,42676E-06	0,000363647	ENSG00000267303	ENSG00000267303
0,636013357	-19,01642421	3,975514829	-4,783386563	1,72366E-06	0,000413476	ENSG00000253859	ENSG00000253859
0,636013357	-19,01642421	3,975514829	-4,783386563	1,72366E-06	0,000413476	ENSG00000264673	ENSG00000264673
0,822243517	-18,87785519	3,883843177	-4,860612112	1,17023E-06	0,000312932	ENSG00000231345	ENSG00000231345
2,392357119	-18,85245765	2,91141652	-6,475355732	9,45888E-11	7,5265E-08	ENSG00000218336	TENM3
3,214481185	-18,80321708	2,681762084	-7,011515747	2,3575E-12	3,01056E-09	ENSG00000249590	ENSG00000249590
3,239656049	-18,15169316	2,666967454	-6,806117236	1,00268E-11	9,62101E-09	ENSG00000285628	ENSG00000285628
3,232347997	-18,13469735	2,626501535	-6,904506663	5,03782E-12	5,47846E-09	ENSG00000265018	ENSG00000265018
1,62147037	-18,10673747	3,277923981	-5,523843009	3,31664E-08	1,27296E-05	ENSG00000272760	ENSG00000272760
0,624648438	-18,07645829	3,998186757	-4,521164064	6,15005E-06	0,001166507	ENSG00000182272	B4GALNT4
0,624648438	-18,07645829	3,998186757	-4,521164064	6,15005E-06	0,001166507	ENSG00000288570	LOC128092248
0,927394141	-17,91087896	4,022500732	-4,452672641	8,4808E-06	0,001487514	ENSG00000019186	CYP24A1
0,927394141	-17,91087896	4,022500732	-4,452672641	8,4808E-06	0,001487514	ENSG00000244528	ENSG00000244528
1,803608675	-17,78757126	3,191565165	-5,573306618	2,49949E-08	1,00671E-05	ENSG00000186076	ENSG00000186076
1,413059517	-17,7782069	3,46903125	-5,124833309	2,97801E-07	9,43249E-05	ENSG00000214914	RPL23AP3
1,15196441	-17,77226146	3,721668678	-4,775347566	1,79397E-06	0,000424105	ENSG00000270025	BMS1P7
1,02251036	-17,74740817	3,882213062	-4,571466813	4,84322E-06	0,000969357	ENSG00000207417	ENSG00000207417
0,833928863	-17,60753923	3,989293525	-4,413698596	1,01619E-05	0,001726683	ENSG00000124116	WFDC3
0,931558443	-17,48435548	3,989540577	-4,382548601	1,17299E-05	0,001903862	ENSG00000271554	ENSG00000271554
0,895243317	-17,46487377	3,989820971	-4,377357755	1,20127E-05	0,001921085	ENSG00000264350	ENSG00000264350
1,287979481	-17,44174851	3,585651896	-4,86431729	1,14853E-06	0,000309665	ENSG00000220392	FCF1P5
1,546887581	-17,43337512	3,361220105	-5,186621102	2,14144E-07	6,91706E-05	ENSG00000232702	ENSG00000232702
0,73088507	-17,42214782	3,994171967	-4,361892269	1,28942E-05	0,002032181	ENSG00000269082	ENSG00000269082
1,366856491	-17,30430154	3,30290887	-5,239109593	1,61353E-07	5,37142E-05	ENSG00000233179	ENSG00000233179
1,44021381	-17,27895768	3,276790615	-5,273134512	1,34113E-07	4,60559E-05	ENSG00000167711	SERPINF2
0,748122433	-17,27195464	3,996676943	-4,321578874	1,54917E-05	0,002339815	ENSG00000276601	ENSG00000276601
0,748122433	-17,27195464	3,996676943	-4,321578874	1,54917E-05	0,002339815	ENSG00000286454	ENSG00000286454
0,761002448	-17,13695168	3,994614697	-4,290013676	1,78662E-05	0,002613755	ENSG00000177151	OR2T35
0,640992599	-17,12164074	4,002324118	-4,277924584	1,88644E-05	0,002711154	ENSG00000231291	ENSG00000231291
0,943358334	-17,03731663	3,990390713	-4,269586078	1,95836E-05	0,002760261	ENSG00000200708	ENSG00000200708
0,717713496	-16,8850921	3,999908323	-4,221369777	2,42822E-05	0,003301582	ENSG00000222067	RNU4-86P
1,185679724	-16,83928956	3,486133036	-4,830363439	1,36284E-06	0,000350089	ENSG00000229806	RPS15P5
0,633735404	-16,83192674	4,002628627	-4,205218198	2,6083E-05	0,003459078	ENSG00000235782	ENSG00000235782
0,926358818	-16,70050171	4,008158087	-4,1666275	3,09139E-05	0,004006105	ENSG00000234975	FTH1P2
0,734077908	-16,6492799	3,999453998	-4,162888211	3,14247E-05	0,004052175	ENSG00000276204	ENSG00000276204
0,642896237	-16,62250076	4,013186618	-4,141970544	3,44335E-05	0,004354097	ENSG00000268391	MTCO3P42
0,642896237	-16,62250076	4,013186618	-4,141970544	3,44335E-05	0,004354097	ENSG00000273204	ENSG00000273204
0,674424544	-16,50855983	4,012027918	-4,114766938	3,87571E-05	0,004771362	ENSG00000257083	LOC102724421
0,949744011	-16,41161147	4,008758427	-4,093938751	4,24106E-05	0,005068149	ENSG00000271672	ENSG00000271672
0,631034115	-16,36509037	4,013731003	-4,07727632	4,55663E-05	0,005405658	ENSG00000250274	LOC100128059
0,595233272	-16,2426715	4,041102813	-4,019366061	5,83549E-05	0,006410006	ENSG00000267046	ENSG00000267046
0,639206196	-16,23224383	4,018012536	-4,039868886	5,34811E-05	0,006016438	ENSG00000223561	LINC03007
0,639206196	-16,23224383	4,018012536	-4,039868886	5,34811E-05	0,006016438	ENSG00000271916	ENSG00000271916
0,639206196	-16,23224383	4,018012536	-4,039868886	5,34811E-05	0,006016438	ENSG00000284640	LOC105378644
0,532969564	-16,2212163	4,021658002	-4,033464879	5,49604E-05	0,00611955	ENSG00000200999	LOC124900179
0,532969564	-16,2212163	4,021658002	-4,033464879	5,49604E-05	0,00611955	ENSG00000215223	ENSG00000215223
0,85167946	-16,19233321	4,013528765	-4,034438061	5,47331E-05	0,00611955	ENSG00000280058	ENSG00000280058
1,374690539	-15,96846169	3,507957263	-4,552068482	5,3121E-06	0,001025456	ENSG00000234965	SHISA8
56,59434959	-9,306753492	1,246021914	-7,469173203	8,07003E-14	1,46265E-10	ENSG00000256500	ENSG00000256500
12,89936403	-5,893792691	1,522983912	-3,869898195	0,000108881	0,01066705	ENSG00000133863	TEX15
5,686680258	-5,859845264	1,692878417	-3,461468471	0,000537237	0,036669438	ENSG00000168229	PTGDR
7,33841842	-5,813143929	1,564102185	-3,716601117	0,000201921	0,017566576	ENSG00000224837	ENSG00000224837
12,46123058	-5,668777459	1,301116395	-4,356856529	1,31944E-05	0,002052566	ENSG00000167768	KRT1

7,244904657	-5,495627423	1,575207266	-3,488828132	0,000485143	0,034332547	ENSG00000266770	RN7SL619P
8,452532214	-5,389797008	1,562253714	-3,450013887	0,000560558	0,037551612	ENSG00000185736	ADARB2
12,47356482	-5,369504892	1,451781657	-3,698562291	0,000216824	0,018517463	ENSG00000203761	ENSG00000203761
14,90427021	-4,590600396	1,004822323	-4,568569278	4,91065E-06	0,00097686	ENSG00000006468	ETV1
48,85807163	-4,441774283	0,633719222	-7,009057216	2,39929E-12	3,01056E-09	ENSG00000041353	RAB27B
14,40522793	-4,128196548	1,117540262	-3,694002524	0,000220752	0,018745192	ENSG00000198618	ENSG00000198618
9,748060079	-4,025537211	1,200904936	-3,352086488	0,00080205	0,048913266	ENSG00000122786	CALD1
18,37345045	-3,680552079	1,020095211	-3,608047601	0,00030851	0,023535825	ENSG00000118785	SPP1
32,98429971	-3,175638534	0,745775768	-4,258168031	2,06109E-05	0,002873547	ENSG00000082438	COBLL1
37,10139095	-2,693024874	0,60098511	-4,481017634	7,4288E-06	0,001331632	ENSG00000255036	SUGT1P4-STRA6LP-CCDC180
28,47694252	-2,676727788	0,633133835	-4,227744026	2,36046E-05	0,003235617	ENSG00000114812	VIPR1
34,48280921	-2,626377485	0,677312257	-3,877646474	0,000105472	0,010364196	ENSG00000114757	PEX5L
74,40646456	-2,553921066	0,405708749	-6,294961768	3,07476E-10	2,33281E-07	ENSG00000124212	PTGIS
43,24462263	-2,341081419	0,547871478	-4,273048538	1,92818E-05	0,002758995	ENSG00000204172	AGAP9
257,5150894	-2,27132054	0,346170192	-6,561282837	5,33468E-11	4,57997E-08	ENSG00000137959	IFI44L
73,08098601	-2,121903693	0,435544702	-4,871839064	1,10564E-06	0,000306504	ENSG00000120885	CLU
53,40107271	-2,054453934	0,613026378	-3,351330396	0,000804243	0,048913266	ENSG00000179978	ENSG00000179978
147,5375274	-2,000183837	0,319615097	-6,258101879	3,89691E-10	2,82517E-07	ENSG00000121989	ACVR2A
337,3550329	-1,968341871	0,264412675	-7,444203922	9,75307E-14	1,59092E-10	ENSG00000091409	ITGA6
58,22938032	-1,932041371	0,475239973	-4,06540165	4,79498E-05	0,005640046	ENSG00000184979	USP18
162,6582109	-1,9217493	0,284801814	-6,747672257	1,50236E-11	1,40037E-08	ENSG00000134326	CMPK2
121,5641776	-1,910513192	0,359479594	-5,314663827	1,06854E-07	3,74841E-05	ENSG00000162630	B3GALT2
255,5326719	-1,907921073	0,333830139	-5,715245126	1,09546E-08	5,17946E-06	ENSG00000245164	LINC00861
44,97825579	-1,867535089	0,519426317	-3,59538019	0,000323918	0,024459862	ENSG00000259205	ENSG00000259205
35,75267111	-1,862686158	0,555475964	-3,353315494	0,000798496	0,048874566	ENSG00000126368	NR1D1
186,472725	-1,823931423	0,316406465	-5,764520093	8,18905E-09	4,11015E-06	ENSG00000137965	IFI44
35,65773115	-1,818827137	0,509578559	-3,569277212	0,000357967	0,026602122	ENSG00000055732	MCOLN3
105,3467557	-1,797606796	0,419594477	-4,28415266	1,83437E-05	0,002654969	ENSG00000196993	NPIPB9
117,1440955	-1,794289732	0,367660358	-4,88029153	1,05929E-06	0,000297917	ENSG00000113319	RASGRF2
187,1767333	-1,73440788	0,366492563	-4,73245041	2,21826E-06	0,000499092	ENSG00000185710	SMG1P4
210,4749437	-1,664774925	0,254331341	-6,545693181	5,92201E-11	4,82999E-08	ENSG00000171476	HOPX
655,0763391	-1,655202087	0,231025381	-7,164589806	7,80198E-13	1,06055E-09	ENSG00000145649	GZMA
110,0004302	-1,616687242	0,378797159	-4,267949761	1,97278E-05	0,002762227	ENSG00000171246	NPTX1
59,51607993	-1,588010127	0,414908481	-3,82737447	0,000129517	0,012354899	ENSG00000049246	PER3
67,61376696	-1,530906061	0,443454826	-3,452225508	0,000555983	0,037476001	ENSG00000143110	C1orf162
127,0212521	-1,510703121	0,416998962	-3,622798279	0,000291433	0,022745726	ENSG00000134321	RSAD2
99,74927718	-1,485522187	0,388304358	-3,825664473	0,00013042	0,012404712	ENSG00000153094	BCL2L11
188,9234691	-1,475363301	0,271810013	-5,427921088	5,70142E-08	2,08992E-05	ENSG00000184613	NELL2
450,5082523	-1,465088	0,233171624	-6,283303137	3,31454E-10	2,45758E-07	ENSG00000137628	DDX60
288,0058433	-1,458585378	0,287198198	-5,078671767	3,80083E-07	0,000118094	ENSG00000163629	PTPN13
65,76958377	-1,445861535	0,431350909	-3,351938077	0,00080248	0,048913266	ENSG00000081377	CDC14B
91,73617378	-1,427413014	0,369977495	-3,858107682	0,000114268	0,011061986	ENSG00000111796	KLRB1
138,0658351	-1,424039168	0,324592501	-4,387159796	1,1484E-05	0,00188269	ENSG00000161048	NAPEPLD
99,06767164	-1,420451151	0,345944144	-4,106012991	4,02547E-05	0,00491861	ENSG00000251079	ENSG00000251079
412,2720656	-1,409635889	0,253326723	-5,564497389	2,62909E-08	1,04599E-05	ENSG00000196693	ZNF33B
491,7670419	-1,402961676	0,310598069	-4,516968446	6,27312E-06	0,001176175	ENSG00000172005	MAL
126,9116117	-1,369431614	0,345020496	-3,969131203	7,21352E-05	0,007591411	ENSG00000246223	LINC01550
322,767055	-1,359212526	0,232007202	-5,858492813	4,67087E-09	2,53971E-06	ENSG00000204177	ENSG00000204177
103,4663613	-1,33222045	0,388173719	-3,432021248	0,000599101	0,039725729	ENSG00000030582	GRN
110,6723091	-1,289253055	0,34640124	-3,721848849	0,000197769	0,017344164	ENSG00000204681	GABBR1
159,4463666	-1,247795358	0,307195441	-4,061894125	4,86762E-05	0,005691795	ENSG00000128872	TMOD2
245,1253346	-1,246967332	0,312377293	-3,991862919	6,55563E-05	0,007058441	ENSG00000116106	EPHA4
1863,384783	-1,193701823	0,248640119	-4,800922024	1,57937E-06	0,000384517	ENSG00000215252	GOLGA8B
392,8642302	-1,17652169	0,2641703	-4,453648614	8,44232E-06	0,001487514	ENSG00000138640	FAM13A
634,7074948	-1,167860713	0,202762491	-5,75974731	8,424E-09	4,16401E-06	ENSG00000151692	RNF144A
291,0077772	-1,165588237	0,259017099	-4,5000436	6,79395E-06	0,001224563	ENSG00000267520	ENSG00000267520
224,2598457	-1,152705183	0,26211346	-4,397733655	1,09387E-05	0,001811495	ENSG00000013375	PGM3
1109,440136	-1,12715977	0,237972644	-4,736509845	2,1743E-06	0,0004926	ENSG00000115738	ID2
169,3305011	-1,105377785	0,277090931	-3,989223976	6,62898E-05	0,007113943	ENSG00000168056	LTBP3
471,4799458	-1,09683503	0,228109861	-4,808363057	1,52171E-06	0,000378964	ENSG00000181381	DDX60L
155,7852454	-1,096396842	0,27654665	-3,964599982	7,35191E-05	0,007687462	ENSG00000181523	SGSH
260,0386579	-1,079419578	0,292596023	-3,689112271	0,000225038	0,018955476	ENSG00000223705	NSUN5P1
251,8944091	-1,076822572	0,312069225	-3,4505888	0,000559365	0,037548822	ENSG00000272419	ENSG00000272419
209,5142177	-1,062112034	0,312852837	-3,394925375	0,000686473	0,044259878	ENSG00000233369	ENSG00000233369
180,7078048	-1,060707494	0,263825775	-4,020484712	5,80785E-05	0,006401195	ENSG00000251474	ENSG00000251474
510,007267	-1,056453705	0,28283673	-3,735206893	0,000187561	0,016582622	ENSG00000272333	KMT2B
424,2139536	-1,049813632	0,286915174	-3,658968666	0,000253232	0,020449135	ENSG00000136111	TBC1D4
567,057328	-1,048468298	0,245541552	-4,270023912	1,95452E-05	0,002760261	ENSG00000166326	TRIM44
111,190591	-1,044805818	0,308036285	-3,391827099	0,000694282	0,044452808	ENSG00000089041	P2RX7
176,4577918	-1,044408299	0,289468962	-3,608014806	0,000308549	0,023535825	ENSG00000117408	IPO13
164,8307866	-1,04345209	0,267318318	-3,903406615	9,48482E-05	0,009521004	ENSG00000134539	KLRD1
244,0570075	-1,039798708	0,300393926	-3,461450505	0,000537273	0,036669438	ENSG00000136161	RCBTB2
675,1558809	-1,034255212	0,262907874	-3,933907324	8,3576E-05	0,008574164	ENSG00000080854	IGSF9B
492,558526	-1,029478421	0,216792798	-4,748674461	2,04754E-06	0,000470416	ENSG00000073910	FRY
425,970656	-1,028324501	0,215691743	-4,767565455	1,86465E-06	0,000437644	ENSG00000184384	MAML2

799,348456	-1,023035254	0,195619219	-5,22972773	1,6976E-07	5,59419E-05	ENSG00000106952	TNFSF8
222,7600848	-1,017532415	0,299706827	-3,395092547	0,000686054	0,044259878	ENSG00000090554	FLT3LG
193,0694045	-1,016794338	0,278413625	-3,652099774	0,000260105	0,020849284	ENSG00000119917	IFIT3
187,9616692	-1,000522634	0,275975287	-3,625406626	0,000288507	0,02259107	ENSG00000138642	HERC6
544,8895279	-1,000262683	0,286132994	-3,495796376	0,000472649	0,033594136	ENSG00000148400	NOTCH1
230,1073074	1,00540825	0,277995219	3,616638639	0,000298453	0,02307286	ENSG00000066827	ZFAT
255,7427818	1,005533963	0,263819084	3,811452709	0,000138152	0,012840703	ENSG00000280721	LINC01943
272,7640764	1,008238031	0,29739911	3,390185103	0,000698454	0,044591738	ENSG00000204257	HLA-DMA
326,9530545	1,008590717	0,248989166	4,050741375	5,10556E-05	0,005864924	ENSG00000172893	DHCR7
324,4653391	1,0127313	0,245118682	4,131595737	3,60254E-05	0,004489892	ENSG00000081913	PHLPP1
112,1025985	1,014160385	0,302378181	3,353946971	0,000796676	0,048854826	ENSG00000132825	PPP1R3D
158,6113131	1,02580175	0,303142184	3,383896413	0,00071465	0,045096187	ENSG00000115325	DOK1
304,5341778	1,036651809	0,248384391	4,173578719	2,99852E-05	0,003928663	ENSG00000114023	FAM162A
693,747533	1,054697092	0,29324616	3,596627126	0,00032237	0,024401395	ENSG00000179029	TMEM107
491,9344261	1,063407413	0,250870643	4,238867483	2,2465E-05	0,003118719	ENSG00000287979	ENSG00000287979
366,4634629	1,070328405	0,256045023	4,180235154	2,91208E-05	0,003830792	ENSG00000285646	ENSG00000285646
466,1263308	1,071278911	0,228490493	4,688505408	2,75208E-06	0,000594617	ENSG00000124610	H1-1
150,167729	1,075206859	0,28118803	3,823800252	0,00013141	0,012462595	ENSG00000143653	SCCPDH
128,8289446	1,090674374	0,320201678	3,406210673	0,000658713	0,042893936	ENSG00000134508	CABLES1
423,9274163	1,107359407	0,221957506	4,98906041	6,06737E-07	0,000178326	ENSG00000128578	STRIP2
468,8932589	1,129394196	0,273417189	4,130662739	3,61719E-05	0,004489892	ENSG00000162433	AK4
192,825432	1,129928389	0,3334814	3,388280095	0,000703324	0,04464055	ENSG00000011590	ZBTB32
232,0112192	1,132066427	0,251523852	4,500831304	6,76882E-06	0,001224563	ENSG00000125726	CD70
220,8552741	1,140288171	0,320275998	3,560329774	0,000370389	0,027400413	ENSG00000067208	EVI5
537,7861349	1,142393028	0,249608498	4,576739329	4,72279E-06	0,000951089	ENSG00000060558	GNA15
311,404889	1,145540402	0,249004486	4,600480974	4,21517E-06	0,000864878	ENSG00000279602	ENSG00000279602
556,9783651	1,181820084	0,316513146	3,733873608	0,000188557	0,016625644	ENSG00000207166	SNORA68
44225,88388	1,190218661	0,235597232	5,051921235	4,37388E-07	0,000134616	ENSG00000265185	ENSG00000265185
186,9887959	1,192044151	0,268946382	4,432274356	9,32443E-06	0,001622877	ENSG00000141698	NT5C3B
177,6354658	1,201341619	0,278725869	4,310118832	1,63167E-05	0,002430664	ENSG00000161956	SENP3
641,7681458	1,204601681	0,231804199	5,196634439	2,02929E-07	6,62035E-05	ENSG00000198814	GK
92,0503494	1,21970929	0,351510033	3,469913158	0,000520627	0,035833174	ENSG00000120280	TASL
392,5587359	1,219956303	0,284346499	4,290386234	1,78363E-05	0,002613755	ENSG00000121039	RDH10
1495,808756	1,222540028	0,212946946	5,741054537	9,40888E-09	4,58142E-06	ENSG00000177606	JUN
94,35949304	1,223993979	0,340994143	3,589486811	0,00033133	0,024829874	ENSG00000243244	STON1
263,5043048	1,23341168	0,272754173	4,52206347	6,12397E-06	0,001166507	ENSG00000104341	LAPTM4B
528,1192227	1,238202913	0,243658128	5,081722184	3,74028E-07	0,00011733	ENSG00000141655	TNFRSF11A
276,2400438	1,285110621	0,375253481	3,42464677	0,000615599	0,040654472	ENSG00000141526	SLC16A3
1060,415925	1,288577937	0,196826368	6,54677496	5,87929E-11	4,82999E-08	ENSG00000179583	CIITA
751,7182729	1,294351711	0,228628401	5,661377599	1,50163E-08	6,48594E-06	ENSG00000198502	HLA-DRB5
192,7059183	1,300138973	0,276513742	4,701896418	2,57756E-06	0,000568122	ENSG00000118777	ABCG2
221,6657163	1,311158694	0,241686546	5,425037983	5,79422E-08	2,10034E-05	ENSG00000242574	HLA-DMB
179,4070228	1,312468179	0,320452158	4,095675894	4,20938E-05	0,005067416	ENSG00000114013	CD86
160,0464324	1,314858835	0,369683906	3,556711057	0,000375527	0,027717616	ENSG00000196576	PLXNB2
177,2779069	1,325949304	0,275623844	4,8107206	1,50387E-06	0,000377402	ENSG00000183780	SLC35F3
117,1885835	1,32667435	0,326959446	4,057611317	4,95772E-05	0,005755894	ENSG00000204475	NCR3
425,3526258	1,336797749	0,218055547	6,130537686	8,75826E-10	5,83121E-07	ENSG00000151914	DST
109,4072141	1,338909619	0,360617057	3,712829417	0,000204955	0,01778312	ENSG00000184792	OSBP2
98,02173172	1,340251168	0,346683702	3,865919165	0,000110672	0,01081003	ENSG00000240350	ENSG00000240350
246,4115531	1,348320714	0,308173142	4,375205133	1,21318E-05	0,001930678	ENSG00000207205	RNVU1-15
129692,7217	1,355266096	0,218709398	6,1966523	5,76767E-10	4,0035E-07	ENSG00000264940	ENSG00000264940
109,9932	1,355588685	0,326220703	4,155434252	3,2467E-05	0,004137517	ENSG00000227630	LINC01132
92,22433865	1,377023819	0,353308577	3,897510306	9,71867E-05	0,009696081	ENSG00000135750	KCNK1
186,767949	1,37762308	0,37863757	3,638368695	0,00027437	0,021725878	ENSG00000222724	RNU2-63P
122,4392336	1,39049847	0,313766332	4,431636951	9,35204E-06	0,001622877	ENSG00000129116	PALLD
1951,936095	1,414072388	0,170767157	8,280704611	1,22449E-16	3,32897E-13	ENSG00000204287	HLA-DRA
930,9010894	1,430077876	0,252649842	5,660315735	1,51095E-08	6,48594E-06	ENSG00000262074	ENSG00000262074
184,8366998	1,438483987	0,325521138	4,419018666	9,91501E-06	0,001693546	ENSG00000214338	MTCL3
930,0545913	1,455112807	0,209358551	6,950338563	3,64411E-12	4,09949E-09	ENSG00000223865	HLA-DPB1
119,5562196	1,463880981	0,335318288	4,365646114	1,26748E-05	0,002007287	ENSG00000147852	VLDLR
62,08323664	1,480292791	0,402521401	3,67755053	0,000235484	0,01949859	ENSG00000148225	WDR31
133,6418289	1,483215753	0,311398009	4,763086822	1,90654E-06	0,000444278	ENSG00000050438	SLC4A8
229,3468894	1,483519559	0,336681571	4,406298673	1,05152E-05	0,001768287	ENSG00000210194	ENSG00000210194
92,7046388	1,522979662	0,430723858	3,535860929	0,000406449	0,02946662	ENSG00000200972	RNU5A-8P
152,0855745	1,531283701	0,339352871	4,512364071	6,4109E-06	0,001188348	ENSG00000177494	ZBED2
175,9733793	1,535148558	0,36366605	4,221313913	2,42882E-05	0,003301582	ENSG00000273338	ENSG00000273338
68,40629946	1,551924095	0,40888397	3,795512197	0,000147339	0,013426776	ENSG00000146416	AIG1
220,7307193	1,561010705	0,312679021	4,992374285	5,96415E-07	0,000176886	ENSG00000212195	LOC124904154
53,45459676	1,56301097	0,465970037	3,354316473	0,000795613	0,048854826	ENSG00000135643	KCNMB4
234,909838	1,578082934	0,300208914	5,256615837	1,4673E-07	4,98639E-05	ENSG00000277925	TERC
137,4657702	1,579914734	0,360862643	4,378160952	1,19685E-05	0,001921085	ENSG00000286172	RNVU1-8
52,7909636	1,580879854	0,463294824	3,412254514	0,000644279	0,042122179	ENSG00000103202	NME4
62,97784639	1,5892044	0,468287936	3,393647961	0,000689683	0,044291761	ENSG00000260035	ENSG00000260035
1556,430452	1,597210765	0,220862147	7,231708955	4,76954E-13	6,76527E-10	ENSG00000196126	HLA-DRB1
145,8348048	1,598295138	0,318986096	5,010547979	5,42753E-07	0,000163952	ENSG00000229391	ENSG00000229391

1406,353855	1,606249374	0,219887781	7,304859623	2,77556E-13	4,11591E-10	ENSG00000179344	HLA-DQB1
172,8644777	1,609502198	0,270583788	5,948258051	2,71011E-09	1,55113E-06	ENSG00000005102	MEOX1
1009,016618	1,627533595	0,289422469	5,623383705	1,87253E-08	7,72024E-06	ENSG00000072110	ACTN1
47,5448985	1,654027995	0,480705219	3,440836355	0,000579919	0,038532143	ENSG00000154319	FAM167A
61,07691035	1,655462338	0,47720298	3,469094719	0,000522215	0,035866847	ENSG00000135919	SERPINE2
262,9659011	1,669698846	0,28520626	5,854355528	4,78863E-09	2,56105E-06	ENSG00000270022	ENSG00000270022
56,11343782	1,679430176	0,465450747	3,608180215	0,000308352	0,023535825	ENSG00000171757	LRRC34
176,2727742	1,696246896	0,358607044	4,730099216	2,2441E-06	0,000501449	ENSG00000020577	SAMD4A
69,58648378	1,698238052	0,410400562	4,138001289	3,50344E-05	0,004412987	ENSG00000214193	SH3D21
138,8417586	1,702275747	0,395583166	4,303205725	1,68344E-05	0,002496394	ENSG00000171169	NAIF1
96,2689003	1,737689829	0,398623998	4,359220317	1,30527E-05	0,00204726	ENSG00000278774	LOC124904146
51,91875581	1,739429134	0,499605707	3,481603812	0,000498421	0,03504412	ENSG00000275291	RNVU1-26
137,4151843	1,744590954	0,288105623	6,055386689	1,40081E-09	8,46295E-07	ENSG00000165474	GJB2
36,16063462	1,760578125	0,494309925	3,561688806	0,000368477	0,027320893	ENSG00000177181	RIMKLA
51,3767284	1,79472854	0,461605805	3,888011202	0,000101069	0,009991739	ENSG00000010278	CD9
65,44668777	1,826270007	0,491200324	3,71797395	0,000200827	0,01754249	ENSG00000155980	KIF5A
196,8321352	1,83885754	0,333217221	5,518494908	3,41915E-08	1,29705E-05	ENSG00000188761	BCL2L15
51,83931417	1,853958269	0,458256025	4,045682251	5,2171E-05	0,005930402	ENSG00000150938	CRIM1
48,573972	1,858086199	0,458672693	4,051006801	5,09977E-05	0,005864924	ENSG00000272768	ENSG00000272768
2397,690729	1,880084528	0,182136174	10,32241147	5,58037E-25	3,64108E-21	ENSG00000231389	HLA-DPA1
41,4173843	1,881953196	0,531653523	3,539811392	0,000400413	0,029093711	ENSG00000198910	L1CAM
119,4982723	1,886769619	0,419227824	4,500583001	6,77673E-06	0,001224563	ENSG00000152207	CYSLTR2
91,7117972	1,895815818	0,358574374	5,287092323	1,24276E-07	4,31317E-05	ENSG00000105383	CD33
170,6589022	1,912409548	0,314196114	6,086674732	1,1528E-09	7,23248E-07	ENSG00000204252	HLA-DOA
68,49039728	1,917099336	0,420625076	4,557739053	5,17072E-06	0,001016203	ENSG00000133454	MYO18B
30,47387737	1,936912355	0,556799616	3,478652465	0,000503942	0,035204696	ENSG00000140465	CYP1A1
95,30937685	1,938232116	0,341946138	5,668238067	1,44273E-08	6,44764E-06	ENSG00000119411	BSPRY
50,71050431	1,94336468	0,467317488	4,158553297	3,2027E-05	0,00409744	ENSG00000071909	MYO3B
71,5275645	2,012076774	0,480875335	4,184196252	2,86177E-05	0,003779851	ENSG00000099250	NRP1
55,48968186	2,018410464	0,458647268	4,400790329	1,07857E-05	0,001795274	ENSG00000179588	ZFPM1
42,90154225	2,034298365	0,51389374	3,958597286	7,53912E-05	0,007808329	ENSG00000224557	HLA-DPB2
27,96615045	2,061697662	0,607440288	3,394074617	0,000688609	0,044291761	ENSG00000128683	GAD1
160,3157824	2,077929155	0,297877985	6,975772833	3,04194E-12	3,67556E-09	ENSG00000133812	SBF2
98,77351014	2,079644821	0,443563175	4,688497464	2,75218E-06	0,000594617	ENSG00000144645	OSBPL10
874,1752952	2,142201761	0,185017462	11,57837609	5,30413E-31	8,6521E-27	ENSG00000196735	HLA-DQA1
31,7897073	2,153410229	0,605832809	3,554462875	0,000378752	0,027892561	ENSG00000108176	DNAJC12
55,66479014	2,157084311	0,454199111	4,749204172	2,04219E-06	0,000470416	ENSG00000073282	TP63
100,1615509	2,187821017	0,369230417	5,925354235	3,11625E-09	1,75284E-06	ENSG00000107242	PIP5K1B
174,8779079	2,253630349	0,42998513	5,241182054	1,59551E-07	5,36618E-05	ENSG00000189221	MAOA
22,30602863	2,26900309	0,656446682	3,456492588	0,000547254	0,037117719	ENSG00000100867	DHRS2
62,34688647	2,321903188	0,654641863	3,546829678	0,000389897	0,028456344	ENSG00000258311	ENSG00000258311
22,7524074	2,396506705	0,715155986	3,351026564	0,000805126	0,048913266	ENSG00000111249	CUX2
29,94066439	2,441128083	0,679856483	3,590652061	0,000329852	0,024795121	ENSG00000165025	SYK
36,70107491	2,44355998	0,531970701	4,593410835	4,36059E-06	0,000889125	ENSG00000122861	PLAU
23,23340603	2,475657634	0,660431838	3,748543744	0,000177864	0,015941334	ENSG00000215284	ENSG00000215284
37,10846454	2,508250678	0,656401266	3,821215479	0,000132796	0,012490965	ENSG00000176014	TUBB6
57,62576523	2,509699962	0,556729745	4,507932229	6,54625E-06	0,001206581	ENSG00000129226	CD68
149,8820457	2,521828898	0,327573317	7,698517452	1,37654E-14	3,20773E-11	ENSG00000132170	PPARG
54,04042451	2,548568403	0,5164563	4,934722267	8,02649E-07	0,000230847	ENSG00000141574	SECTM1
142,9473423	2,576556789	0,42134218	6,115117146	9,64861E-10	6,17208E-07	ENSG00000166025	AMOTL1
27,18550658	2,591472065	0,637457679	4,065324099	4,79658E-05	0,005640046	ENSG00000162373	BEND5
38,98177744	2,662886218	0,736118965	3,617467211	0,0002975	0,023053777	ENSG00000255526	NEDD8-MDP1
21,81744014	2,732000349	0,71524291	3,819681835	0,000133624	0,012490965	ENSG00000143079	CTTNBP2NL
40,81327389	2,818678045	0,519111333	5,429814124	5,64128E-08	2,08992E-05	ENSG00000154734	ADAMTS1
24,28376913	2,88550598	0,772463851	3,735457621	0,000187374	0,016582622	ENSG00000120658	ENOX1
38,25071097	2,885992492	0,774994115	3,723889556	0,000196177	0,017250865	ENSG00000172116	CD8B
25,10266262	2,908097991	0,827093525	3,516044925	0,000438027	0,031407007	ENSG00000136237	RAPGEF5
29,09502494	2,915538499	0,820971997	3,55132515	0,000383297	0,028038251	ENSG00000120549	KIAA1217
16,89453334	2,933325548	0,76120932	3,853507135	0,000116438	0,011238666	ENSG00000106066	CPVL
190,6644432	2,934941056	0,313881455	9,350476156	8,7254E-21	3,55822E-17	ENSG00000140479	PCSK6
20,53844781	2,957363382	0,808069485	3,659788468	0,000252424	0,020434406	ENSG00000087303	NID2
54,2359054	2,963083168	0,554718483	5,341598051	9,21308E-08	3,26704E-05	ENSG00000144642	RBMS3
74,49968827	2,98179309	0,555691585	5,365913705	8,05404E-08	2,88742E-05	ENSG00000109265	CRACD
32,5062841	3,112026731	0,548961976	5,668929479	1,43692E-08	6,44764E-06	ENSG00000170365	SMAD1
82,81672478	3,121684697	0,416428328	7,496331267	6,56288E-14	1,25946E-10	ENSG00000115414	FN1
22,44370436	3,131578713	0,703302645	4,452675865	8,48067E-06	0,001487514	ENSG00000128322	IGLL1
19,77976905	3,18568103	0,794542721	4,009452163	6,08598E-05	0,006618298	ENSG00000187079	TEAD1
20,46169093	3,203633369	0,824962812	3,883367008	0,00010302	0,01015384	ENSG00000141404	GNAL
16,65385637	3,305182461	0,908830007	3,636744426	0,000276106	0,02181034	ENSG00000153165	RGPD3
22,18372144	3,40896467	0,849620251	4,012339237	6,012E-05	0,006559718	ENSG00000147642	SYBU
12,74192775	3,441426344	0,999295169	3,443853679	0,000573486	0,038260545	ENSG00000279970	ENSG00000279970
48,74170765	3,494455609	0,547988853	6,376873532	1,8074E-10	1,40392E-07	ENSG00000115008	IL1A
19,35023167	3,733162197	0,844669473	4,419672212	9,88507E-06	0,001693546	ENSG00000152463	OLAH
91,23578866	4,082042856	0,499506393	8,172153372	3,02933E-16	7,60222E-13	ENSG00000136099	PCDH8
19,25959287	4,090468573	0,856406335	4,776317507	1,78534E-06	0,000424105	ENSG00000196951	SCOC-AS1

49,36767519	4,26726077	0,619653036	6,886532498	5,71687E-12	6,01636E-09	ENSG00000158125	XDH
14,8230985	4,315803939	1,053972851	4,094796117	4,2254E-05	0,005067995	ENSG00000042832	TG
12,6952079	4,3473591	1,196288005	3,634040533	0,000279017	0,021987079	ENSG00000286132	ENSG00000286132
153,2402767	4,353579943	1,106917174	3,933067484	8,38687E-05	0,008577216	ENSG00000113721	PDGFRB
13,02248381	4,422029072	1,060536723	4,169614286	3,05116E-05	0,003981636	ENSG00000154096	THY1
19,48333292	4,456567422	1,059299504	4,207089125	2,58681E-05	0,003444577	ENSG00000187045	TMPRSS6
165,6595613	4,535385244	0,42384555	10,70056119	1,01164E-26	8,25091E-23	ENSG00000164303	ENPP6
331,5243535	4,564052757	0,664699629	6,866338654	6,58706E-12	6,71551E-09	ENSG00000169429	CXCL8
19015,80497	5,009496209	0,17719867	28,27050682	7,9673E-176	2,5992E-171	ENSG00000173757	STAT5B
29,68023426	5,034582359	0,82297253	6,11755821	9,50201E-10	6,17208E-07	ENSG00000157110	RBPMS
118,1019313	5,07589854	0,46139898	11,00110482	3,77478E-28	4,10495E-24	ENSG00000151474	FRMD4A
10,04937143	5,163037264	1,356485728	3,806186205	0,000141126	0,013005931	ENSG00000074047	GLI2
7,247876169	5,169172304	1,489034826	3,471491877	0,000517575	0,035698449	ENSG00000116990	MYCL
6,963782903	5,430435229	1,482184852	3,663804297	0,000248497	0,020369235	ENSG00000144407	PTH2R
30,69738857	5,500652341	0,94757728	5,804964362	6,43797E-09	3,38762E-06	ENSG00000254126	CD8B2
6,389667762	5,555703943	1,642505963	3,382455874	0,000718408	0,045245847	ENSG00000091428	RAPGEF4
9,672714539	5,607165731	1,599084535	3,506484871	0,000454067	0,032415371	ENSG00000283201	ZNF724
20,91651503	5,665711662	1,022450022	5,54130915	3,00219E-08	1,18004E-05	ENSG00000277481	PKD1L3
7,861544642	5,92370315	1,571478957	3,769508414	0,000163569	0,014781961	ENSG00000109625	CPZ
88,12071373	5,935848584	0,623066375	9,526831848	1,62156E-21	7,55741E-18	ENSG00000152377	SPOCK1
39,88575751	8,544168361	1,291091353	6,61778761	3,64614E-11	3,21491E-08	ENSG00000274049	INO80B-WBP1
0,630221196	14,07076406	4,05003825	3,47422992	0,000512322	0,035440393	ENSG00000171759	PAH
0,630221196	14,07076406	4,05003825	3,47422992	0,000512322	0,035440393	ENSG00000233741	RPL27P7
0,676189846	14,40680667	4,014755342	3,588464412	0,000332631	0,024832417	ENSG00000142871	CCN1
0,613172142	14,45598968	4,016313042	3,599318461	0,000319052	0,024206422	ENSG00000115598	IL1RL2
0,613172142	14,45598968	4,016313042	3,599318461	0,000319052	0,024206422	ENSG00000250306	ENSG00000250306
0,812656632	14,63889284	4,012710756	3,648130584	0,000264155	0,021070428	ENSG00000182489	XKRX
0,808159552	14,76563044	4,008139268	3,683911525	0,000229682	0,019213193	ENSG00000259992	ENSG00000259992
0,808159552	14,76563044	4,008139268	3,683911525	0,000229682	0,019213193	ENSG00000271286	CYTH1P1
1,14671293	14,76887014	3,44125596	4,29170928	1,77303E-05	0,002613755	ENSG00000270038	ENSG00000270038
0,725577138	14,77913104	4,001421453	3,693470236	0,000221214	0,018745192	ENSG00000242255	ENSG00000242255
0,725577138	14,77913104	4,001421453	3,693470236	0,000221214	0,018745192	ENSG00000257335	MGAM
1,148568997	14,83581483	3,729451055	3,978015696	6,94928E-05	0,007354531	ENSG00000205704	SMIM45
0,925061628	14,84287422	3,998772495	3,711857636	0,000205744	0,017804198	ENSG00000269524	ENSG00000269524
0,914396184	14,84537455	4,006676595	3,705159176	0,000211258	0,018207834	ENSG00000288704	ENSG00000288704
1,31652518	14,95374978	3,54998439	4,212342404	2,52736E-05	0,003379205	ENSG00000243721	ENSG00000243721
6,528662471	14,98985627	3,980701974	3,765631381	0,000166129	0,014971779	ENSG00000281039	ENSG00000281039
0,837982134	15,08365384	3,993683073	3,776878025	0,000158806	0,014431481	ENSG00000201498	ENSG00000201498
0,967436185	15,18177458	3,974332761	3,819955572	0,000133476	0,012490965	ENSG00000261239	ENSG00000261239
0,919097516	15,18896193	3,795438819	4,001898767	6,28362E-05	0,006810524	ENSG00000239699	HNRNPA3P8
0,82093308	15,20306778	3,992179816	3,808212175	0,000139975	0,0129364	ENSG00000284250	MIR6516
0,87266268	15,20769663	3,990897328	3,810595807	0,000138632	0,012848691	ENSG00000243136	RN7SL22P
1,596886359	15,28181281	3,312451444	4,613445077	3,96049E-06	0,000822975	ENSG00000261771	DNAAF4-CCPG1
0,813959254	15,28836404	3,9931321	3,828664731	0,00012884	0,012326359	ENSG00000225563	ENSG00000225563
0,946818191	15,29439887	3,990045827	3,833138648	0,000126519	0,012139831	ENSG00000166763	ENSG00000166763
0,794792592	15,32583053	4,009152923	3,82271039	0,000131993	0,012481543	ENSG00000148926	ADM
0,674372688	15,4647101	4,001198854	3,865019125	0,00011108	0,010817582	ENSG00000269481	ENSG00000269481
0,838399001	15,54648115	3,99632188	3,89019744	0,000100163	0,009945867	ENSG00000283886	ENSG00000283886
0,75442091	15,55661205	3,999268069	3,88986479	0,0001003	0,009945867	ENSG00000231362	ENSG00000231362
1,140604926	15,55788647	3,531926579	4,404929187	1,05818E-05	0,00177037	ENSG00000224814	ENSG00000224814
1,094320433	15,59096082	3,786596329	4,117407685	3,83158E-05	0,004734903	ENSG00000180834	MAP6D1
1,510082472	15,59544874	3,399369662	4,587747227	4,48055E-06	0,000907909	ENSG00000204196	ENSG00000204196
1,038769246	15,59987597	3,85025898	4,051643293	5,08592E-05	0,005864924	ENSG00000187726	DNAJB13
1,091824877	15,60298457	3,77254741	4,135928028	3,53523E-05	0,004435898	ENSG00000230953	ENSG00000230953
0,933280788	15,62961893	3,995520615	3,911785331	9,16163E-05	0,009224973	ENSG00000285068	ENSG00000285068
0,922377093	15,64219414	3,994443881	3,91598796	9,00347E-05	0,009122029	ENSG00000241002	RPL32P13
1,206725429	15,76096853	3,650325818	4,317688151	1,57672E-05	0,002370457	ENSG00000262117	BCAR4
0,921686137	15,91323265	3,994191011	3,984094052	6,7738E-05	0,007221849	ENSG00000200741	ENSG00000200741
0,921686137	15,91323265	3,994191011	3,984094052	6,7738E-05	0,007221849	ENSG00000285629	ENSG00000285629
1,34755733	15,93509041	3,499660939	4,553324074	5,28048E-06	0,00102542	ENSG00000288000	ENSG00000288000
1,34755733	15,93509041	3,499660939	4,553324074	5,28048E-06	0,00102542	ENSG00000288635	ENSG00000288635
1,593510868	15,93565074	3,309416512	4,815244826	1,4702E-06	0,000371811	ENSG00000170788	DYDC1
2,348622734	16,14637285	2,916061722	5,5370477	3,07613E-08	1,19471E-05	ENSG00000254633	ENSG00000254633
1,449832882	16,15795177	3,261917551	4,953513236	7,28854E-07	0,000212305	ENSG00000272482	ENSG00000272482
0,966203218	16,17620401	3,954219657	4,090871376	4,29755E-05	0,005116913	ENSG00000254641	ENSG00000254641
1,823987246	16,43778427	3,02221121	5,438992555	5,35827E-08	2,00929E-05	ENSG00000130751	NPAS1
2,467699422	16,64841393	2,880541993	5,779611605	7,48733E-09	3,87725E-06	ENSG00000277599	ENSG00000277599
2,299247083	16,6746696	2,914251565	5,721767401	1,05422E-08	5,05775E-06	ENSG00000272104	ENSG00000272104
1,25621659	16,78036425	3,851747487	4,356558759	1,32123E-05	0,002052566	ENSG00000282780	TRBJ1-6
3,620947309	17,22455994	2,576948003	6,684092936	2,32359E-11	2,10569E-08	ENSG00000260371	ENSG00000260371
2,62746998	17,23950351	2,849226165	6,050591463	1,44315E-09	8,56024E-07	ENSG00000273167	ENSG00000273167
5,390229318	17,92378205	3,965995741	4,519364926	6,20254E-06	0,001169663	ENSG00000288534	TMX2-CTNND1
1,739020882	18,09538365	3,218964143	5,621492768	1,89314E-08	7,72024E-06	ENSG00000285547	ENSG00000285547
0,898163443	18,23420304	3,761376617	4,847747222	1,24871E-06	0,000328533	ENSG00000254012	RPL10P8
6,125240702	18,43304913	3,965558421	4,648285859	3,34705E-06	0,000705391	ENSG00000228589	ENSG00000228589

4,093012685	19,0591378	3,969282659	4,801657992	1,57357E-06	0,000384517	ENSG00000284976	LOC122513141
6,352708034	23,02758232	3,920661032	5,873392811	4,26965E-09	2,3609E-06	ENSG00000144785	ENSG00000144785

ensembl_gene_id	baseMean	log2FoldChange	lfcSE	stat	pvalue	padj	hgnc_symbol	gene_biotype
ENSG00000003400	38,17543049	2,202415806	0,379333404	5,806015984	6,39769E-09	1,60274E-07	CASP10	protein_coding
ENSG00000005884	600,0823205	2,256944337	0,103228629	21,86355044	5,7771E-106	4,0726E-102	ITGA3	protein_coding
ENSG00000006283	99,62152007	2,136434505	0,267058625	7,999870843	1,2455E-15	8,85107E-14	CACNA1G	protein_coding
ENSG00000006611	112,5025588	3,955490463	0,34007289	11,63130194	2,85706E-31	9,32461E-29	USH1C	protein_coding
ENSG00000007516	219,3636063	2,291245152	0,179551037	12,76096862	2,70812E-37	1,59824E-34	BAIAP3	protein_coding
ENSG00000009709	16,26917718	6,010632709	1,068387076	5,625894251	1,84549E-08	4,12196E-07	PAX7	protein_coding
ENSG00000010310	139,6725342	2,06053162	0,202525395	10,17418889	2,58545E-24	4,57949E-22	GIPR	protein_coding
ENSG00000013016	46,74533462	2,763343201	0,493459387	5,599940484	2,14425E-08	4,70322E-07	EHD3	protein_coding
ENSG00000015520	21,63789286	4,003009538	0,591879133	6,763221266	1,34957E-11	5,69014E-10	NPC1L1	protein_coding
ENSG00000019505	29,27880986	4,946770973	0,878543817	5,630647987	1,79534E-08	4,02302E-07	SYT13	protein_coding
ENSG00000021300	207,9633419	2,171349857	0,189154581	11,47923483	1,67759E-30	5,14187E-28	PLEKHB1	protein_coding
ENSG00000023445	41,60082792	2,301370939	0,372203082	6,183105549	6,28527E-10	1,92982E-08	BIRC3	protein_coding
ENSG00000027644	80,42153086	2,785695267	0,292407775	9,52674827	1,62287E-21	2,15049E-19	INSRR	protein_coding
ENSG00000029534	158,9072004	2,320318483	0,245555836	9,449250016	3,41267E-21	4,35833E-19	ANK1	protein_coding
ENSG00000036530	55,10944722	2,87528239	0,324345948	8,864862992	7,65982E-19	7,7739E-17	CYP46A1	protein_coding
ENSG00000054179	78,94974214	2,704872889	0,269383671	10,04096825	1,00681E-23	1,68991E-21	ENTPD2	protein_coding
ENSG00000062038	123,7972813	2,335281723	0,213675182	10,92912007	8,36557E-28	2,16816E-25	CDH3	protein_coding
ENSG00000063127	104,0879335	2,087275013	0,264915092	7,87903398	3,29922E-15	2,23207E-13	SLC6A16	protein_coding
ENSG00000064300	429,5951477	3,382387612	0,17634418	19,18060242	5,3754E-82	1,89472E-78	NGFR	protein_coding
ENSG00000065361	671,8656728	2,368899701	0,110530187	21,43215134	6,7011E-102	3,93669E-98	ERBB3	protein_coding
ENSG00000066923	196,013419	2,325605086	0,187229659	12,42113618	2,00691E-35	9,68732E-33	STAG3	protein_coding
ENSG00000067842	72,01482231	3,168467221	0,246159201	12,87161809	6,50255E-38	3,95175E-35	ATP2B3	protein_coding
ENSG00000072163	170,1484635	2,361081118	0,18023359	13,10011699	3,28751E-39	2,14589E-36	LIMS2	protein_coding
ENSG00000072818	63,80343939	2,732443982	0,276022348	9,899357803	4,18957E-23	6,77403E-21	ACAP1	protein_coding
ENSG00000073861	48,81304673	3,449996688	0,406771102	8,481420313	2,22461E-17	1,97018E-15	TBX21	protein_coding
ENSG00000074660	70,96936509	2,066043906	0,251207343	8,22445667	1,96078E-16	1,55661E-14	SCARF1	protein_coding
ENSG00000075673	44,15268178	3,329279145	0,375791891	8,859369315	8,04684E-19	8,12708E-17	ATP12A	protein_coding
ENSG00000079308	651,7935611	3,539756081	0,130029878	27,22263624	3,5051E-163	1,2355E-158	TNS1	protein_coding
ENSG00000080031	21,58864789	3,492209398	0,602725552	5,794029118	6,87175E-09	1,70695E-07	PTPRH	protein_coding
ENSG00000081479	86,53195937	2,479594674	0,276635991	8,963384196	3,14863E-19	3,36312E-17	LRP2	protein_coding
ENSG00000084636	35,074958	4,453977769	0,593376805	7,506154156	6,08897E-14	3,61929E-12	COL16A1	protein_coding
ENSG00000085117	117,9585234	2,100700562	0,220487174	9,527540877	1,61053E-21	2,14219E-19	CD82	protein_coding
ENSG00000087245	30,5706812	3,080989982	0,530530381	5,80737709	6,34591E-09	1,59585E-07	MMP2	protein_coding
ENSG00000088340	342,2642788	2,478367376	0,186977109	13,25492406	4,22544E-40	3,10288E-37	FER1L4	transcribed_unitary_pseudogene
ENSG00000088827	14,58811672	6,051051442	1,074193288	5,633112316	1,76986E-08	3,97604E-07	SIGLEC1	protein_coding
ENSG00000090339	181,7975527	3,549463772	0,199145895	17,82343426	4,64926E-71	1,09251E-67	ICAM1	protein_coding
ENSG00000091536	86,52467451	4,141895044	0,344943791	12,00744917	3,24707E-33	1,2717E-30	MYO15A	protein_coding
ENSG00000092068	84,38062302	2,87903615	0,275368455	10,45521408	1,38685E-25	2,90975E-23	SLC7A8	protein_coding
ENSG00000095303	109,9574369	3,34181956	0,267628065	12,4868054	8,81215E-36	4,5016E-33	PTGS1	protein_coding
ENSG00000099994	228,0496234	2,459542349	0,182594138	13,46999623	2,34878E-41	1,83977E-38	SUSD2	protein_coding
ENSG00000100346	58,1955877	2,852836886	0,301269965	9,469370379	2,81536E-21	3,65318E-19	CACNA1I	protein_coding
ENSG00000101198	13,8500675	6,87444816	1,223708596	5,617716656	1,93497E-08	4,27611E-07	NKAIN4	protein_coding
ENSG00000101203	419,0435605	7,36258565	0,377341824	19,51171372	8,73057E-85	3,84669E-81	COL20A1	protein_coding
ENSG00000101204	61,10926766	4,061232006	0,394428597	10,29649481	7,30756E-25	1,37742E-22	CHRNA4	protein_coding
ENSG00000101850	65,044158	2,124677125	0,229899154	9,241778776	2,42433E-20	2,84843E-18	GPR143	protein_coding
ENSG00000102445	28,03047282	4,50837597	0,605322046	7,447896536	9,48401E-14	5,4623E-12	RUBCNL	protein_coding
ENSG00000103196	47,2828225	3,302203143	0,403098995	8,192040134	2,56835E-16	1,98965E-14	CRISPLD2	protein_coding
ENSG00000103489	21,05397307	4,335201265	0,659043865	6,578016268	4,76766E-11	1,84064E-09	XYLT1	protein_coding
ENSG00000104848	63,94631845	2,655014283	0,298528905	8,893659012	5,9129E-19	6,11196E-17	KCNA7	protein_coding
ENSG00000104856	141,0278826	2,293915202	0,219216497	10,46415407	1,26199E-25	2,67967E-23	RELB	protein_coding
ENSG00000104870	33,0219089	2,613720695	0,449391476	5,816133228	6,02245E-09	1,52646E-07	FCGRT	protein_coding
ENSG00000104888	160,2314889	4,175629337	0,288741219	14,46149374	2,12143E-47	2,49254E-44	SLC17A7	protein_coding
ENSG00000104967	260,3824365	2,039122793	0,178127978	11,4475155	2,41982E-30	7,35291E-28	NOVA2	protein_coding
ENSG00000105122	56,06307414	2,71742321	0,378283137	7,18356951	6,79145E-13	3,52037E-11	RASAL3	protein_coding
ENSG00000105357	53,33016608	7,824863674	1,034519691	7,563764849	3,91567E-14	2,3674E-12	MYH14	protein_coding
ENSG00000105559	170,0018099	2,1230631	0,199831475	10,62426776	2,29809E-26	5,25993E-24	PLEKHA4	protein_coding
ENSG00000105639	32,80124725	3,851014428	0,574956659	6,697921253	2,11405E-11	8,5947E-10	JAK3	protein_coding
ENSG00000107147	29,21054287	2,560727146	0,410155088	6,243314349	4,28395E-10	1,36405E-08	KCNT1	protein_coding
ENSG00000107738	81,07975052	3,411428862	0,315871602	10,80004925	3,44019E-27	8,42082E-25	VSIR	protein_coding
ENSG00000107796	38,61148429	2,160539682	0,383224569	5,637790097	1,72246E-08	3,8869E-07	ACTA2	protein_coding
ENSG00000107859	20,53411021	3,323089213	0,540931762	6,143268794	8,08403E-10	2,43752E-08	PITX3	protein_coding
ENSG00000108176	59,84432933	2,414334286	0,334224508	7,223690143	5,05955E-13	2,66575E-11	DNAJC12	protein_coding
ENSG00000108370	44,34048549	5,137635414	0,632216361	8,12638794	4,42272E-16	3,30981E-14	RGS9	protein_coding
ENSG00000108387	149,3357064	2,286052287	0,206485901	11,071227	1,73015E-28	4,69111E-26	SEPTIN4	protein_coding
ENSG00000108846	62,03154257	2,78091928	0,293737345	9,467367125	2,86986E-21	3,70538E-19	ABCC3	protein_coding
ENSG00000109339	32,47301188	2,942550949	0,421489991	6,981306818	2,92447E-12	1,38365E-10	MAPK10	protein_coding
ENSG00000110169	75,48935649	2,224711865	0,253266698	8,784067867	1,57666E-18	1,56108E-16	HPX	protein_coding
ENSG00000110436	51,50091762	3,29795318	0,385936066	8,545335541	1,28163E-17	1,16131E-15	SLC1A2	protein_coding
ENSG00000110786	19,75259327	4,598245908	0,791793114	5,807383051	6,34568E-09	1,59585E-07	PTPN5	protein_coding
ENSG00000112139	320,0026817	2,160074045	0,180603514	11,9603102	5,73473E-33	2,19715E-30	MDGA1	protein_coding
ENSG00000112182	5821,12613	5,452496993	0,235814357	23,12198911	2,7827E-118	4,9042E-114	BACH2	protein_coding
ENSG00000113140	75,49491918	2,869546255	0,411171226	6,978956874	2,9738E-12	1,4051E-10	SPARC	protein_coding
ENSG00000115380	19,86409129	4,481210768	0,584332593	7,668938578	1,73425E-14	1,07621E-12	EFEMP1	protein_coding
ENSG00000115423	52,50804759	2,633369363	0,317148321	8,303273859	1,01281E-16	8,2257E-15	DNAH6	protein_coding
ENSG00000115596	42,17552152	4,170244239	0,463490356	8,997477915	2,30962E-19	2,48959E-17	WNT6	protein_coding
ENSG00000115828	65,09475549	4,104362053	0,430531675	9,533240631	1,52449E-21	2,03542E-19	QPCT	protein_coding
ENSG00000116299	49,06309292	2,819049515	0,29801575	9,459397761	3,09722E-21	3,98433E-19	ELAPOR1	protein_coding
ENSG00000116981	66,65257918	2,186445564	0,270281098	8,089524492	5,98981E-16	4,4077E-14	NT5C1A	protein_coding
ENSG00000116983	186,6042251	2,819039646	0,221373415	12,73431882	3,81158E-37	2,20247E-34	HPCAL4	protein_coding
ENSG00000117115	241,0472994	2,426481542	0,204868169	11,844112	2,30851E-32	8,21921E-30	PADI2	protein_coding
ENSG00000117971	62,58754707	2,140271363	0,287084125	7,455206243	8,97274E-14	5,18477E-12	CHRNB4	protein_coding
ENSG00000118160	44,13915242	4,234385389	0,484709584	8,735922561	2,41676E-18	2,33386E-16	SLC8A2	protein_coding
ENSG00000119922	62,44794168	2,57072432	0,321089664	8,006250626	1,18259E-15	8,47231E-14	IFIT2	protein_coding
ENSG00000121101	59,48247272	2,04453132	0,288116865	7,096187575	1,28245E-12	6,38474E-11	TEX14	protein_coding
ENSG00000122176	14,38398387	3,864665466	0,694587473	5,563972311	2,63702E-08	5,67112E-07	FMOD	protein_coding
ENSG00000122733	60,94914948	3,242032923	0,318527024	10,17820365	2,48099E-24	4,41666E-22	PHF24	protein_coding
ENSG00000122735	81,85834923	2,006889823	0,219633124	9,137464262	6,39338E-20	7,22288E-18	DNAI1	protein_coding
ENSG00000122986	85,49434103	2,558348301	0,21873288	11,69622188	1,33255E-31	4,47332E-29	HVCN1	protein_coding

ENSG00000123095	22,18488543	2,337644463	0,428474154	5,455742054	4,87687E-08	9,82848E-07	BHLHE41	protein_coding
ENSG00000123453	57,41692086	4,801004613	0,480447389	9,992779073	1,6392E-23	2,72541E-21	SARDH	protein_coding
ENSG00000124466	18,20793617	3,411682165	0,58659353	5,816092389	6,02392E-09	1,52646E-07	LYPD3	protein_coding
ENSG00000124772	128,4547558	3,411899468	0,252788317	13,49706155	1,62738E-41	1,30368E-38	CPNE5	protein_coding
ENSG00000125531	24,09089	3,000233406	0,449970157	6,667627532	2,59972E-11	1,04487E-09	FNDC11	protein_coding
ENSG00000125637	149,1336634	2,202100609	0,196809143	11,18901578	4,61528E-29	1,3226E-26	PSD4	protein_coding
ENSG00000126856	11,21499846	3,443046483	0,607090515	5,67138902	1,41644E-08	3,26319E-07	PRDM7	protein_coding
ENSG00000128340	38,41452422	2,423618161	0,359640857	6,738995618	1,59485E-11	6,62055E-10	RAC2	protein_coding
ENSG00000129009	21,70462098	2,076069469	0,378533207	5,48451082	4,14615E-08	8,48192E-07	ISLR	protein_coding
ENSG00000129244	195,248251	2,777970826	0,187847728	14,78841853	1,73994E-49	2,2594E-46	ATP1B2	protein_coding
ENSG00000129910	32,90903158	4,371036325	0,603617095	7,241405792	4,44058E-13	2,36795E-11	CDH15	protein_coding
ENSG00000129993	41,92189405	2,572761723	0,318981593	8,065549161	7,29074E-16	5,28774E-14	CBFA2T3	protein_coding
ENSG00000130635	16,67456414	5,282305956	0,659995872	8,003543928	1,20889E-15	8,60824E-14	COL5A1	protein_coding
ENSG00000130720	19,73590948	4,092769823	0,726609883	5,632692204	1,77418E-08	3,97833E-07	FIBCD1	protein_coding
ENSG00000131044	38,10114478	2,077194772	0,305304643	6,803678941	1,01981E-11	4,3677E-10	TTLL9	protein_coding
ENSG00000131095	25,39295638	2,631123852	0,466416161	5,641150703	1,68917E-08	3,81912E-07	GFAP	protein_coding
ENSG00000131398	84,5904858	2,012327611	0,219293593	9,176408585	4,45709E-20	5,10076E-18	KCNC3	protein_coding
ENSG00000131409	93,08016815	2,631330833	0,211842142	12,42118687	2,00564E-35	9,68732E-33	LRRC4B	protein_coding
ENSG00000131620	14,81946826	4,530828167	0,805392206	5,625617098	1,84846E-08	4,1222E-07	ANO1	protein_coding
ENSG00000131831	30,39697986	2,430437012	0,396808593	6,124960636	9,0706E-10	2,7072E-08	RAI2	protein_coding
ENSG00000132031	24,177042	2,409677244	0,435489933	5,533255908	3,1434E-08	6,62275E-07	MATN3	protein_coding
ENSG00000132470	325,6261233	2,345054367	0,151808677	15,44743297	7,85084E-54	1,25785E-50	ITGB4	protein_coding
ENSG00000132669	91,33079032	2,164025587	0,273809731	7,9033918	2,71415E-15	1,85764E-13	RIN2	protein_coding
ENSG00000132821	134,3298519	3,129205862	0,211628317	14,78632872	1,7948E-49	2,2594E-46	VSTM2L	protein_coding
ENSG00000134202	133,3099966	2,402494154	0,220375095	10,9018406	1,12949E-27	2,90601E-25	GSTM3	protein_coding
ENSG00000134668	29,04793169	2,194553771	0,394346039	5,565045806	2,62084E-08	5,6432E-07	SPOCD1	protein_coding
ENSG00000135253	52,53387293	2,709942009	0,384833786	7,041850557	1,89703E-12	9,18497E-11	KCP	protein_coding
ENSG00000135472	151,1634624	2,860748949	0,222008377	12,88577029	5,41323E-38	3,40724E-35	FAIM2	protein_coding
ENSG00000135636	111,6216189	2,971010278	0,246311222	12,06201751	1,67626E-33	6,79134E-31	DYSF	protein_coding
ENSG00000135929	30,65981492	2,070995547	0,37027617	5,593110529	2,23037E-08	4,86787E-07	CYP27A1	protein_coding
ENSG00000136931	70,29593293	2,996504268	0,322551675	9,289997544	1,54292E-20	1,84355E-18	NR5A1	protein_coding
ENSG00000137474	33,00823762	5,769736127	0,878854659	6,56506291	5,20109E-11	1,98837E-09	MYO7A	protein_coding
ENSG00000139567	43,82437808	5,901797735	0,86906817	6,790949133	1,11398E-11	4,73651E-10	ACVRL1	protein_coding
ENSG00000139973	32,67936719	3,053674618	0,521551739	5,85497927	4,7707E-09	1,23736E-07	SYT16	protein_coding
ENSG00000140279	23,80177759	2,425229181	0,430966238	5,627422677	1,82922E-08	4,09114E-07	DUOX2	protein_coding
ENSG00000141314	83,81559369	3,149352249	0,25741412	12,23457457	2,03167E-34	8,95152E-32	RHBDL3	protein_coding
ENSG00000141485	62,41351389	3,528032386	0,39680897	8,891009655	6,05561E-19	6,24118E-17	SLC13A5	protein_coding
ENSG00000141505	65,37748526	2,393533849	0,279673948	8,558301083	1,14543E-17	1,04326E-15	ASGR1	protein_coding
ENSG00000141837	71,50100902	6,080258481	0,585243373	10,38928206	2,77434E-25	5,55624E-23	CACNA1A	protein_coding
ENSG00000142233	39,12327797	4,753762628	0,519450066	9,15152954	5,61331E-20	6,36199E-18	NTN5	protein_coding
ENSG00000142606	24,77043762	2,995854299	0,481097636	6,227123298	4,75077E-10	1,49781E-08	MMEL1	protein_coding
ENSG00000142609	171,8405596	3,602534436	0,196920377	18,29437104	9,1754E-75	2,3101E-71	CFAP74	protein_coding
ENSG00000142621	18,54460559	4,737013954	0,799157796	5,927507657	3,07567E-09	8,30101E-08	FHAD1	protein_coding
ENSG00000142677	24,77013325	3,104270328	0,565475273	5,489665911	4,02695E-08	8,26204E-07	IL22RA1	protein_coding
ENSG00000143195	87,79582677	3,720088151	0,36384425	10,22439725	1,54184E-24	2,78702E-22	ILDR2	protein_coding
ENSG00000143382	753,1059867	2,374698799	0,12369987	19,19726195	3,90122E-82	1,52789E-78	ADAMTSL4	protein_coding
ENSG00000143416	476,5570321	10,78321163	1,026142257	10,50849582	7,89426E-26	1,7071E-23	SELENBP1	protein_coding
ENSG00000143502	120,4820064	2,313474435	0,194945725	11,86727451	1,75079E-32	6,36204E-30	SUSD4	protein_coding
ENSG00000143603	306,6005563	2,489798093	0,176803097	14,08232176	4,87804E-45	4,91261E-42	KCNN3	protein_coding
ENSG00000143850	242,2820346	4,161801037	0,236454755	17,60083461	2,42721E-69	5,34714E-66	PLEKHA6	protein_coding
ENSG00000143851	51,14985771	2,796359557	0,326850353	8,555473569	1,17386E-17	1,0664E-15	PTPN7	protein_coding
ENSG00000144891	30,93196936	3,771750098	0,538276182	7,007090831	2,43324E-12	1,16531E-10	AGTR1	protein_coding
ENSG00000145526	34,16233112	3,573614759	0,562159657	6,356939204	2,05813E-10	6,94212E-09	CDH18	protein_coding
ENSG00000145794	42,54085402	3,174030809	0,392310179	8,09061549	5,9364E-16	4,37753E-14	MEGF10	protein_coding
ENSG00000146005	53,5039062	2,136830151	0,299669073	7,130632902	9,99085E-13	5,03082E-11	PSD2	protein_coding
ENSG00000148357	138,5209186	4,849398242	0,341523686	14,19930285	9,25283E-46	9,59246E-43	HMCN2	protein_coding
ENSG00000148848	47,87005454	2,76191806	0,288724645	9,565924185	1,11203E-21	1,49607E-19	ADAM12	protein_coding
ENSG00000149260	516,7088159	2,744313424	0,14039464	19,54713822	4,3632E-85	2,19706E-81	CAPN5	protein_coding
ENSG00000149654	35,15108547	3,088246025	0,455428484	6,780968109	1,19373E-11	5,06338E-10	CDH22	protein_coding
ENSG00000154783	27,56954341	2,666753044	0,42821097	6,227661666	4,73448E-10	1,49401E-08	FGD5	protein_coding
ENSG00000155980	35,76339107	2,852147539	0,494656665	5,765913497	8,12167E-09	1,988E-07	KIF5A	protein_coding
ENSG00000156042	198,0241579	2,031687069	0,171841387	11,82303692	2,96759E-32	1,04602E-29	CFAP70	protein_coding
ENSG00000156804	117,5358332	2,056392013	0,197953505	10,38825768	2,80429E-25	5,5845E-23	FBXO32	protein_coding
ENSG00000156966	22,45594777	2,668768562	0,42402053	6,293960725	3,09466E-10	1,00814E-08	B3GNT7	protein_coding
ENSG00000157087	55,70403505	3,58587652	0,347008794	10,33367622	4,96216E-25	9,82619E-23	ATP2B2	protein_coding
ENSG00000157103	37,79162273	3,825217268	0,422739609	9,048637003	1,44764E-19	1,59957E-17	SLC6A1	protein_coding
ENSG00000157423	33,53042305	2,458923103	0,432528421	5,684997754	1,30814E-08	3,03352E-07	HYDIN	protein_coding
ENSG00000158445	34,6049428	2,523877315	0,354837737	7,112764667	1,13741E-12	5,70291E-11	KCNB1	protein_coding
ENSG00000158825	29,59030788	2,512611895	0,434597786	5,781465018	7,40529E-09	1,83045E-07	CDA	protein_coding
ENSG00000159708	30,24040437	2,542361031	0,421263871	6,035079689	1,58884E-09	4,52371E-08	LRRC36	protein_coding
ENSG00000160111	47,09603347	2,787886487	0,42616192	6,541847956	6,07633E-11	2,29805E-09	CPAMD8	protein_coding
ENSG00000160179	18,30693674	5,839399871	1,060334219	5,507131401	3,64728E-08	7,57568E-07	ABCG1	protein_coding
ENSG00000160460	164,1581496	2,647382151	0,181731204	14,56757064	4,51669E-48	5,48981E-45	SPTBN4	protein_coding
ENSG00000160539	69,62110148	3,497247072	0,352059602	9,933678999	2,97087E-23	4,84803E-21	PLPP7	protein_coding
ENSG00000160712	338,4667966	2,125738946	0,181830115	11,69079692	1,42047E-31	4,72348E-29	IL6R	protein_coding
ENSG00000160808	19,47482869	4,114420318	0,607901337	6,768236998	1,30361E-11	5,50296E-10	MYL3	protein_coding
ENSG00000161082	65,2355563	2,194723399	0,267515912	8,204085442	2,32353E-16	1,81194E-14	CELF5	protein_coding
ENSG00000161270	21,53136647	2,893310331	0,526813653	5,492094437	3,97195E-08	8,16346E-07	NPHS1	protein_coding
ENSG00000161405	36,42927008	2,054285137	0,344112344	5,969809483	2,37531E-09	6,50038E-08	IKZF3	protein_coding
ENSG00000161642	254,3086608	2,846307918	0,19922249	14,28708134	2,6342E-46	2,81365E-43	ZNF385A	protein_coding
ENSG00000161681	243,6822011	2,223060011	0,164295383	13,53087331	1,02795E-41	8,42633E-39	SHANK1	protein_coding
ENSG00000162426	33,17659478	2,001647087	0,326005392	6,139920194	8,25629E-10	2,48309E-08	SLC45A1	protein_coding
ENSG00000162745	129,9929893	2,022525653	0,219396273	9,218596213	3,01015E-20	3,49019E-18	OLFML2B	protein_coding
ENSG00000163075	48,0094885	2,672755525	0,338910475	7,886317252	3,11235E-15	2,10969E-13	CFAP221	protein_coding
ENSG00000163235	64,59063083	3,436996966	0,33293067	10,32346153	5,51965E-25	1,0749E-22	TGFA	protein_coding
ENSG00000163239	40,12742841	2,901404644	0,367246883	7,90042007	2,77965E-15	1,89878E-13	TDRD10	protein_coding
ENSG00000163462	62,29061386	2,003002317	0,268600886	7,45716943	8,8401E-14	5,11652E-12	TRIM46	protein_coding
ENSG00000163520	107,4344374	3,575883754	0,300855962	11,88570015	1,40453E-32	5,21126E-30	FBLN2	protein_coding
ENSG00000163637	26,71897658	2,723782185	0,462182837	5,89330015	3,78558E-09	1,00607E-07	PRICKLE2	protein_coding

ENSG00000166428	43,44224647	2,358073317	0,315694963	7,469467666	8,05199E-14	4,70674E-12	PLD4	protein_coding
ENSG00000166501	77,64726001	2,037440344	0,264559702	7,701249763	1,34742E-14	8,46592E-13	PRKCB	protein_coding
ENSG00000166546	78,01003981	2,330563403	0,263757894	8,835994882	9,92113E-19	9,93466E-17	BEAN1	protein_coding
ENSG00000166897	143,5448493	2,026360409	0,193018853	10,49825124	8,79952E-26	1,89125E-23	ELFN2	protein_coding
ENSG00000167080	36,11620095	3,367076363	0,436946269	7,705927709	1,29896E-14	8,19066E-13	B4GALNT2	protein_coding
ENSG00000168016	95,5553875	2,660591828	0,233781091	11,38069728	5,21808E-30	1,54561E-27	TRANK1	protein_coding
ENSG00000168070	21,82112092	2,84036902	0,463295514	6,130793265	8,7442E-10	2,62397E-08	MAJIN	protein_coding
ENSG00000168071	207,1734025	3,12440749	0,200884831	15,55322758	1,51286E-54	2,66626E-51	CCDC88B	protein_coding
ENSG00000168477	126,1223942	2,979552736	0,236481822	12,59950009	2,12487E-36	1,10143E-33	TNXB	protein_coding
ENSG00000168546	17,14167412	4,378271266	0,783467605	5,588324566	2,29271E-08	4,98233E-07	GFRA2	protein_coding
ENSG00000169064	23,59455979	2,775817646	0,454208034	6,111335418	9,88009E-10	2,9265E-08	ZBBX	protein_coding
ENSG00000169181	61,97330386	8,032831351	1,035675427	7,75612817	8,75615E-15	5,6218E-13	GSG1L	protein_coding
ENSG00000169220	95,60908272	2,141163065	0,236672167	9,046957634	1,47007E-19	1,61928E-17	RGS14	protein_coding
ENSG00000169291	128,2900061	2,273287914	0,201389202	11,28803278	1,50364E-29	4,3443E-27	SHE	protein_coding
ENSG00000169994	24,28834931	3,469514574	0,519942655	6,67287929	2,50833E-11	1,01044E-09	MYO7B	protein_coding
ENSG00000170421	222,2503444	2,091213236	0,164285819	12,72911596	4,07426E-37	2,31628E-34	KRT8	protein_coding
ENSG00000170442	13,23024352	4,135022446	0,74379836	5,559332564	2,70808E-08	5,80977E-07	KRT86	protein_coding
ENSG00000170703	37,55692612	2,450991776	0,366680262	6,68427519	2,3207E-11	9,41314E-10	TTLL6	protein_coding
ENSG00000171992	168,4756059	3,061249373	0,217501498	14,07461285	5,4402E-45	5,32656E-42	SYNPO	protein_coding
ENSG00000172005	30,09470292	3,832327389	0,556265259	6,889388346	5,60328E-12	2,52241E-10	MAL	protein_coding
ENSG00000172350	158,7075642	2,325155126	0,192877267	12,05510201	1,82307E-33	7,30221E-31	ABCG4	protein_coding
ENSG00000172794	24,48212037	3,162237377	0,554980562	5,697924565	1,21275E-08	2,82905E-07	RAB37	protein_coding
ENSG00000172818	25,52282361	4,292720897	0,615617974	6,973027233	3,10193E-12	1,45782E-10	OVOL1	protein_coding
ENSG00000173210	50,74359587	2,391762013	0,332832725	7,186078273	6,66788E-13	3,46141E-11	ABLIM3	protein_coding
ENSG00000173546	642,8241115	2,650119553	0,144106667	18,3899857	1,58022E-75	4,64162E-72	CSPG4	protein_coding
ENSG00000173641	19,88210274	3,042468476	0,506108368	6,011496092	1,83819E-09	5,15864E-08	HSPB7	protein_coding
ENSG00000173698	181,8311626	2,140466654	0,178817024	11,97015033	5,09363E-33	1,97297E-30	ADGRG2	protein_coding
ENSG00000173838	14,62799024	3,637006617	0,588119527	6,184128313	6,24466E-10	1,91902E-08	MARCHF10	protein_coding
ENSG00000174292	197,7667965	2,061497789	0,168758262	12,21568509	2,56333E-34	1,10186E-31	TNK1	protein_coding
ENSG00000174348	31,01562375	3,351055361	0,488434033	6,86081464	6,8469E-12	3,03677E-10	PODN	protein_coding
ENSG00000174358	13,61722985	4,924339147	0,795556715	6,189802759	6,02395E-10	1,86093E-08	SLC6A19	protein_coding
ENSG00000174403	52,80696112	2,318961429	0,321166461	7,22043461	5,18217E-13	2,72628E-11	MIR1-1HG-AS1	lncRNA
ENSG00000174460	258,9302181	2,993599606	0,286918205	10,43363422	1,74099E-25	3,6098E-23	ZCCHC12	protein_coding
ENSG00000174844	32,17621975	2,915135856	0,411850772	7,078136187	1,46106E-12	7,19266E-11	DNAH12	protein_coding
ENSG00000175147	23,23196597	2,359904644	0,406168003	5,810168759	6,24099E-09	1,57581E-07	TMEM51-AS1	lncRNA
ENSG00000175170	90,79893133	6,299254891	0,534672306	11,78152454	4,86059E-32	1,67967E-29	FAM182B	lncRNA
ENSG00000175264	31,28823814	3,907579572	0,550317817	7,100587067	1,24228E-12	6,20225E-11	CHST1	protein_coding
ENSG00000175591	169,0977583	3,068620641	0,17862118	17,17948926	3,78226E-66	7,84219E-63	P2RY2	protein_coding
ENSG00000175985	32,51268074	2,543085268	0,390629882	6,510216916	7,50424E-11	2,76683E-09	PLEKHD1	protein_coding
ENSG00000177098	244,1807548	3,63987941	0,19586921	18,58321384	4,39386E-77	1,40795E-73	SCN4B	protein_coding
ENSG00000177103	29,69341852	6,994040771	1,0479583	6,673968584	2,48977E-11	1,00411E-09	DSCAML1	protein_coding
ENSG00000177108	49,09942928	2,436205782	0,363490185	6,702260158	2,0522E-11	8,37266E-10	ZDHHC22	protein_coding
ENSG00000177301	72,10759455	3,551932312	0,356095188	9,97467091	1,96753E-23	3,22564E-21	KCNA2	protein_coding
ENSG00000177692	29,7365375	2,440023199	0,412199211	5,919524188	3,22874E-09	8,66769E-08	DNAJC28	protein_coding
ENSG00000179057	36,24220304	2,491883578	0,320385416	7,777768442	7,3815E-15	4,81975E-13	IGSF22	protein_coding
ENSG00000179388	105,1878836	2,638353058	0,472650694	5,582035717	2,3772E-08	5,15322E-07	EGR3	protein_coding
ENSG00000179630	24,25067493	2,918884113	0,47066245	6,2016507	5,5874E-10	1,73826E-08	LACC1	protein_coding
ENSG00000179930	20,99924027	4,653652501	0,773757974	6,01435159	1,80608E-09	5,08067E-08	ZNF648	protein_coding
ENSG00000180638	160,3207462	3,08426784	0,168086664	18,34927149	3,34568E-75	9,07143E-72	SLC47A2	protein_coding
ENSG00000180712	25,56282506	2,989756353	0,416524329	7,177867282	7,08072E-13	3,64352E-11	LINC02363	lncRNA
ENSG00000182040	63,42443178	2,993999423	0,337594888	8,868615982	7,40606E-19	7,56663E-17	USH1G	protein_coding
ENSG00000182256	45,64411068	2,012563127	0,260150848	7,736139023	1,02481E-14	6,50858E-13	GABRG3	protein_coding
ENSG00000183508	71,91176717	3,003318322	0,320002813	9,385287254	6,27452E-21	7,73301E-19	TENT5C	protein_coding
ENSG00000183682	37,10800662	2,393408291	0,356915335	6,705815239	2,00285E-11	8,18984E-10	BMP8A	protein_coding
ENSG00000183914	93,0181299	3,959403709	0,317280862	12,47917599	9,69851E-36	4,88362E-33	DNAH2	protein_coding
ENSG00000184185	27,59986107	2,528171434	0,419883017	6,021132863	1,73201E-09	4,8918E-08	KCNJ12	protein_coding
ENSG00000184454	33,60086497	4,016985311	0,493216492	8,144466731	3,80957E-16	2,89396E-14	NCMAP	protein_coding
ENSG00000184497	46,24431071	2,205949215	0,311077271	7,091322375	1,32836E-12	6,59468E-11	TMEM255B	protein_coding
ENSG00000184574	16,78578139	3,268315488	0,546879055	5,976304012	2,28257E-09	6,27093E-08	LPAR5	protein_coding
ENSG00000184584	98,85294963	2,110536288	0,243234584	8,676958064	4,06495E-18	3,84132E-16	STING1	protein_coding
ENSG00000184828	31,81840414	2,015821644	0,365602111	5,513703507	3,5136E-08	7,31527E-07	ZBTB7C	protein_coding
ENSG00000184985	37,11313988	2,295689725	0,317999869	7,219153043	5,23124E-13	2,74391E-11	SORCS2	protein_coding
ENSG00000185101	130,0980583	2,622916342	0,238404716	11,00194822	3,73964E-28	9,83692E-26	ANO9	protein_coding
ENSG00000185513	80,55294616	2,333153177	0,298964514	7,804114083	5,9921E-15	3,9701E-13	L3MBTL1	protein_coding
ENSG00000185567	1345,784646	3,199699886	0,141480443	22,61584586	3,0267E-113	3,5562E-109	AHNAK2	protein_coding
ENSG00000185974	21,45522505	4,176730859	0,554469902	7,532836036	4,96499E-14	2,96621E-12	GRK1	protein_coding
ENSG00000186409	75,52118562	2,130411217	0,245652257	8,67246752	4,22855E-18	3,98524E-16	CCDC30	protein_coding
ENSG00000186510	37,96503529	3,859651121	0,468521162	8,23794405	1,75195E-16	1,40347E-14	CLCNKA	protein_coding
ENSG00000186642	110,1477549	2,967938996	0,254912815	11,6429572	2,49222E-31	8,20988E-29	PDE2A	protein_coding
ENSG00000187122	491,2863301	2,116482603	0,132510331	15,97220822	1,99593E-57	3,90847E-54	SLIT1	protein_coding
ENSG00000187416	8,367705779	7,107808684	1,256044286	5,658883817	1,52361E-08	3,48448E-07	LHFPL3	protein_coding
ENSG00000187554	32,48806765	2,012393583	0,350402445	5,743092304	9,2963E-09	2,23455E-07	TLR5	protein_coding
ENSG00000187678	25,59153587	2,556005721	0,46704808	5,472682212	4,43275E-08	9,0211E-07	SPRY4	protein_coding
ENSG00000187800	90,42599617	3,146125146	0,28915623	10,88036438	1,4299E-27	3,65226E-25	PEAR1	protein_coding
ENSG00000187902	24,21213633	2,811543247	0,477654959	5,886138511	3,95323E-09	1,04691E-07	SHISA7	protein_coding
ENSG00000187997	30,01185762	2,289284462	0,37407856	6,119795969	9,36952E-10	2,78698E-08	C17orf99	protein_coding
ENSG00000188162	80,73670584	6,733100716	0,621025859	10,84190073	2,17896E-27	5,4471E-25	OTOG	protein_coding
ENSG00000188263	17,9402011	3,53330525	0,61707504	5,72589235	1,02891E-08	2,44059E-07	IL17REL	protein_coding
ENSG00000188389	42,5837011	3,469377005	0,381331048	9,098071135	9,19503E-20	1,02891E-17	PDCD1	protein_coding
ENSG00000188596	88,37659924	3,10021843	0,287091944	10,79869531	3,49129E-27	8,48697E-25	CFAP54	protein_coding
ENSG00000188672	32,22698711	2,64334468	0,374219867	7,063613973	1,62227E-12	7,90896E-11	RHCE	protein_coding
ENSG00000188783	19,84793134	3,1527466	0,528105196	5,969921569	2,37368E-09	6,50038E-08	PRELP	protein_coding
ENSG00000189120	32,01089699	4,502199812	0,567013057	7,940204828	2,01848E-15	1,40054E-13	SP6	protein_coding
ENSG00000196092	60,21736558	3,49793104	0,379108869	9,226719082	2,79048E-20	3,25692E-18	PAX5	protein_coding
ENSG00000196169	81,5569693	3,328853495	0,3125313	10,65126434	1,72013E-26	3,98888E-24	KIF19	protein_coding
ENSG00000196337	29,55256542	2,104197249	0,377075743	5,580303927	2,40099E-08	5,2016E-07	CGB7	protein_coding
ENSG00000196668	25,42454525	2,957507746	0,45408702	6,513085852	7,36224E-11	2,72588E-09	LINC00173	lncRNA
ENSG00000197177	46,26780958	3,935316276	0,383788543	10,25386595	1,13703E-24	2,08739E-22	ADGRA1	protein_coding
ENSG00000197467	75,4480125	2,061762126	0,252657772	8,160295689	3,34205E-16	2,5498E-14	COL13A1	protein_coding

ENSG00000197558	349,4068344	2,779064944	0,193122942	14,39013365	5,96805E-47	6,5738E-44	SSPOP	transcribed_unitary_pseudogene
ENSG00000197748	67,56042686	2,894608221	0,280980206	10,30182255	6,91388E-25	1,3173E-22	CFAP43	protein_coding
ENSG00000198771	22,9188964	5,277256158	0,871750716	6,053629853	1,41618E-09	4,07157E-08	RCSD1	protein_coding
ENSG00000198959	107,8962046	3,386032897	0,267625653	12,65212382	1,08891E-36	5,81543E-34	TGM2	protein_coding
ENSG00000203867	22,8819153	3,271347379	0,476663082	6,86301814	6,74207E-12	3,00056E-10	RBM20	protein_coding
ENSG00000204176	35,07021404	2,51050708	0,364786893	6,882119756	5,89684E-12	2,64107E-10	SYT15	protein_coding
ENSG00000204653	26,26243523	3,633971514	0,617525101	5,884734904	3,98693E-09	1,05425E-07	ASPDH	protein_coding
ENSG00000205108	32,88877491	2,382852051	0,36119825	6,597075286	4,19348E-11	1,63677E-09	FAM205A	protein_coding
ENSG00000205181	18,15922783	3,133030233	0,55316168	5,663859853	1,48005E-08	3,3942E-07	LINC00654	lncRNA
ENSG00000205336	39,367077	7,923189195	1,198386172	6,611549247	3,80319E-11	1,50117E-09	ADGRG1	protein_coding
ENSG00000205517	49,01626637	2,216051061	0,268296644	8,25970473	1,46033E-16	1,1752E-14	RGL3	protein_coding
ENSG00000205832	33,43511939	2,231853655	0,367653914	6,070528756	1,2749E-09	3,70161E-08	C16orf96	protein_coding
ENSG00000205838	77,77825065	2,065922821	0,24521406	8,424977033	3,60821E-17	3,06463E-15	TTC23L	protein_coding
ENSG00000206549	19,44217915	2,45744865	0,420152563	5,848934614	4,94705E-09	1,27466E-07	ENSG00000206549	protein_coding
ENSG00000213937	26,47689174	2,28291849	0,379884673	6,009504074	1,86092E-09	5,20171E-08	CLDN9	protein_coding
ENSG00000215644	20,4788848	4,025102957	0,670828734	6,000194618	1,97081E-09	5,47417E-08	GCGR	protein_coding
ENSG00000218730	46,08102436	6,499117837	0,674089326	9,641330294	5,34902E-22	7,51165E-20	ENSG00000218730	processed_pseudogene
ENSG00000222078	80,39645476	5,690516843	0,618220766	9,204667907	3,42729E-20	3,96083E-18	RN7SKP110	misc_RNA
ENSG00000224514	33,00729954	2,294087065	0,399328395	5,744863366	9,19952E-09	2,21644E-07	LINC00620	lncRNA
ENSG00000224940	146,5391305	2,917684712	0,208619918	13,98564787	1,90732E-44	1,81701E-41	PRRT4	protein_coding
ENSG00000225950	31,75173152	2,573480861	0,413446096	6,22446526	4,83201E-10	1,51799E-08	NTF4	protein_coding
ENSG00000226455	57,5752517	4,256836848	0,452845282	9,40020139	5,44587E-21	6,7829E-19	ENSG00000226455	lncRNA
ENSG00000226869	8,572941445	6,953322716	1,251334228	5,556727019	2,7488E-08	5,88638E-07	LHFPL3-AS1	lncRNA
ENSG00000228013	13,71514211	4,472452832	0,802785732	5,57116632	2,5304E-08	5,46181E-07	IL6R-AS1	lncRNA
ENSG00000228140	21,66856158	3,322861695	0,485046648	6,850602329	7,35397E-12	3,25235E-10	EFHD2-AS1	lncRNA
ENSG00000229089	23,66742858	2,382681054	0,414982069	5,741648205	9,37594E-09	2,25125E-07	ANKRD20A8P	transcribed_unprocessed_pseudogene
ENSG00000229162	30,30952364	2,870365382	0,389612968	7,367222384	1,7422E-13	9,6707E-12	RUNX3-AS1	lncRNA
ENSG00000229474	91,7226923	2,123204946	0,245213096	8,658611539	4,77545E-18	4,46486E-16	PATL2	protein_coding
ENSG00000231301	21,90509235	3,170080547	0,513944794	6,168134366	6,91004E-10	2,10871E-08	RPL13AP	processed_pseudogene
ENSG00000232022	28,74561382	2,984153235	0,460376677	6,481981782	9,05256E-11	3,29258E-09	FAAHP1	transcribed_unprocessed_pseudogene
ENSG00000232871	24,69194789	3,739012067	0,51540012	7,254581289	4,02906E-13	2,1583E-11	SEC1P	transcribed_unitary_pseudogene
ENSG00000233217	18,93548325	6,609360087	1,064253864	6,210322847	5,28759E-10	1,65228E-08	MROH3P	transcribed_unitary_pseudogene
ENSG00000235151	21,6640103	2,613909429	0,402453537	6,494934669	8,30695E-11	3,05003E-09	ENSG00000235151	lncRNA
ENSG00000242866	31,34185633	2,094557801	0,338765961	6,182905142	6,29325E-10	1,93059E-08	STRC	protein_coding
ENSG00000243323	86,20875275	4,488438975	0,386709503	11,60674598	3,80834E-31	1,22033E-28	PTPRVP	unitary_pseudogene
ENSG00000244509	58,21111876	4,502990313	0,572599401	7,864119845	3,71702E-15	2,50991E-13	APOBEC3C	protein_coding
ENSG00000246323	22,47311337	4,253814335	0,57150999	7,443114578	9,83387E-14	5,64535E-12	FAM13B-AS1	lncRNA
ENSG00000248485	112,6022887	2,637713267	0,259105451	10,18007632	2,4337E-24	4,35448E-22	PCP4L1	protein_coding
ENSG00000248596	17,33702581	3,82849952	0,61668159	6,208227362	5,35856E-10	1,67297E-08	ENSG00000248596	lncRNA
ENSG00000255346	25,71613081	3,744713585	0,584234548	6,409606548	1,45896E-10	5,06653E-09	NOX5	protein_coding
ENSG00000255367	29,46971315	2,043506493	0,361131197	5,658626304	1,52589E-08	3,48448E-07	ENSG00000255367	lncRNA
ENSG00000255974	34,91623082	2,19715149	0,369745242	5,94233878	2,80984E-09	7,61856E-08	CYP2A6	protein_coding
ENSG00000256235	642,0764804	3,340880664	0,148953	22,42909287	2,0477E-111	1,8044E-107	SMIM3	protein_coding
ENSG00000257335	35,76907282	5,877396836	1,063382641	5,527076153	3,25612E-08	6,81946E-07	MGAM	protein_coding
ENSG00000257743	25,63764666	8,042341122	1,222685177	6,577605806	4,78084E-11	1,84371E-09	MGAM2	protein_coding
ENSG00000258811	34,24427425	2,148021059	0,372984803	5,759004232	8,46116E-09	2,05824E-07	ENSG00000258811	lncRNA
ENSG00000259417	88,11571761	3,728702463	0,257957352	14,45472455	2,34064E-47	2,66139E-44	CTXND1	protein_coding
ENSG00000260220	20,42165955	7,147777275	1,221161199	5,853262681	4,82022E-09	1,24654E-07	CCDC187	protein_coding
ENSG00000260401	117,6464787	3,741282948	0,241335248	15,50243068	3,34013E-54	5,60633E-51	ENSG00000260401	lncRNA
ENSG00000262155	39,3924242	2,163409509	0,2993129	7,227919365	4,9045E-13	2,5957E-11	LINC02175	lncRNA
ENSG00000262943	40,98324488	2,95029401	0,38648345	7,633687827	2,28132E-14	1,40335E-12	ALOX12P2	transcribed_unprocessed_pseudogene
ENSG00000264589	20,93237171	3,514637024	0,521549081	6,738842335	1,59654E-11	6,62055E-10	MAPT-AS1	lncRNA
ENSG00000267226	50,87650999	4,330072888	0,393387133	11,00715433	3,52977E-28	9,35469E-26	MALT1-AS1	lncRNA
ENSG00000267750	38,643259	2,373381114	0,350652785	6,768465032	1,30156E-11	5,50088E-10	RUNDC3A-AS1	lncRNA
ENSG00000271447	23,20734697	3,635510158	0,550677972	6,601880491	4,05975E-11	1,59175E-09	MMP28	protein_coding
ENSG00000275395	73,76799311	2,520035206	0,281746599	8,944332304	3,74205E-19	3,97289E-17	FCGBP	protein_coding
ENSG00000275620	16,31415814	5,542590334	0,887147132	6,247656261	4,16657E-10	1,33391E-08	ENSG00000275620	lncRNA
ENSG00000277152	62,17946397	3,19192774	0,327886788	9,734847087	2,1414E-22	3,145E-20	ENSG00000277152	lncRNA
ENSG00000278195	15,68168754	7,642219623	1,23526411	6,186709031	6,14332E-10	1,89449E-08	SSTR3	protein_coding
ENSG00000279108	39,06601539	2,037281754	0,347754297	5,858394193	4,67364E-09	1,21398E-07	ENSG00000279108	TEC

ensembl_gene_id	baseMean	log2FoldChange	lfcSE	stat	pvalue	padj	hgnc_symbol	gene_biotype
ENSG00000167105	16,5699056	3,05142164	0,561817749	5,431337202	5,59333E-08	9,52143E-07	TMEM92	protein_coding
ENSG00000140519	19,8612697	2,308395501	0,42477098	5,434447296	5,49665E-08	9,37081E-07	RHCG	protein_coding
ENSG00000229162	30,30952364	2,145013456	0,39464533	5,435294152	5,47061E-08	9,33105E-07	RUNX3-AS1	lncRNA
ENSG00000260220	20,42165955	6,648535718	1,222960126	5,436428853	5,4359E-08	9,28108E-07	CCDC187	protein_coding
ENSG00000072952	17,32678809	2,954767301	0,543261304	5,438943068	5,35976E-08	9,15563E-07	IRAG1	protein_coding
ENSG00000145708	29,59814114	-2,175234574	0,399017526	-5,451476271	4,99534E-08	8,58442E-07	CRHBP	protein_coding
ENSG00000249006	20,55744169	2,425928515	0,442906549	5,477292043	4,31884E-08	7,50459E-07	ENSG00000249006	processed_pseudogene
ENSG00000153237	26,44299374	2,144403645	0,391100672	5,482996574	4,18181E-08	7,28493E-07	CCDC148	protein_coding
ENSG00000112539	14,60348547	3,317000643	0,604259403	5,489365368	4,0338E-08	7,05936E-07	C6orf118	protein_coding
ENSG00000152760	11,83620777	6,846355588	1,241061881	5,516530393	3,45758E-08	6,12595E-07	DYNLT5	protein_coding
ENSG00000111181	21,6110929	2,415913161	0,437687742	5,519718573	3,39543E-08	6,02206E-07	SLC6A12	protein_coding
ENSG00000074966	16,67694599	3,204910722	0,579950181	5,52618281	3,27273E-08	5,82553E-07	TXK	protein_coding
ENSG00000215644	20,4788848	3,722484835	0,671166272	5,546293055	2,9179E-08	5,24839E-07	GCGR	protein_coding
ENSG00000080031	21,58864789	3,335405106	0,601288983	5,547091662	2,9046E-08	5,22746E-07	PTPRH	protein_coding
ENSG00000257335	35,76907282	5,90371626	1,061865502	5,55975898	2,70147E-08	4,8873E-07	MGAM	protein_coding
ENSG00000188282	12,41113603	4,486919045	0,804979687	5,573953128	2,49023E-08	4,53623E-07	RUFY4	protein_coding
ENSG00000123243	15,74736214	3,240223437	0,579326464	5,593087209	2,23067E-08	4,09825E-07	ITIH5	protein_coding
ENSG00000204176	35,07021404	2,063911268	0,366806944	5,626696288	1,83694E-08	3,42439E-07	SYT15	protein_coding
ENSG00000240219	24,12641831	2,273074943	0,400217772	5,679595219	1,35014E-08	2,58288E-07	ENSG00000240219	lncRNA
ENSG00000162692	31,61464606	-2,186781968	0,384784854	-5,683129007	1,32252E-08	2,53147E-07	VCAM1	protein_coding
ENSG00000137699	10,51172075	3,852859881	0,677335939	5,688255503	1,28344E-08	2,46215E-07	TRIM29	protein_coding
ENSG00000141506	18,25342339	2,876826089	0,505579704	5,690153431	1,26925E-08	2,43766E-07	PIK3R5	protein_coding
ENSG00000175985	32,51268074	2,240974467	0,391029319	5,730962757	9,98622E-09	1,94845E-07	PLEKHD1	protein_coding
ENSG00000271447	23,20734697	3,17909176	0,55272673	5,751651922	8,83756E-09	1,74417E-07	MMP28	protein_coding
ENSG00000131831	30,39697986	2,278353168	0,395138281	5,76596417	8,11923E-09	1,61917E-07	RAI2	protein_coding
ENSG00000260776	11,88361601	7,155727604	1,240053796	5,770497723	7,90377E-09	1,57804E-07	GOLGA6FP	transcribed_unprocessed_pseudogene
ENSG00000104870	33,0219089	2,574801528	0,445905977	5,774314908	7,72668E-09	1,54719E-07	FCGRT	protein_coding
ENSG00000155980	35,76339107	2,840586109	0,490272132	5,793896746	6,87717E-09	1,38896E-07	KIF5A	protein_coding
ENSG00000154783	27,56954341	2,475854858	0,426969882	5,798663945	6,68453E-09	1,35512E-07	FGD5	protein_coding
ENSG00000267121	25,14055091	2,223573857	0,383080439	5,804456798	6,4575E-09	1,31297E-07	FMNL1-DT	lncRNA
ENSG00000102445	28,03047282	3,554024171	0,61168754	5,810195462	6,23999E-09	1,27101E-07	RUBCNL	protein_coding
ENSG00000187554	32,48806765	2,016792845	0,346689306	5,817291763	5,98087E-09	1,22186E-07	TLR5	protein_coding
ENSG00000003400	38,17543049	2,186048532	0,375690583	5,818747213	5,92903E-09	1,21199E-07	CASP10	protein_coding
ENSG00000200818	19,18190101	-2,447862845	0,420251146	-5,824761849	5,71941E-09	1,17263E-07	RNU6-1204P	snRNA
ENSG00000142606	24,77043762	2,800158511	0,480271172	5,830369745	5,53047E-09	1,13525E-07	MMEL1	protein_coding
ENSG00000205832	33,43511939	2,136078662	0,365475783	5,844651717	5,07629E-09	1,04956E-07	C16orf96	protein_coding
ENSG00000198846	62,51102885	-2,131027594	0,36310069	-5,868971481	4,38507E-09	9,1436E-08	TOX	protein_coding
ENSG00000159208	27,57291636	2,678047587	0,456020258	5,87265048	4,28882E-09	8,97017E-08	CIART	protein_coding
ENSG00000164342	24,19455123	2,448036172	0,41648268	5,877882298	4,15548E-09	8,71787E-08	TLR3	protein_coding
ENSG00000198771	22,9188964	5,133466346	0,870894063	5,894478518	3,75866E-09	7,92903E-08	RCSD1	protein_coding
ENSG00000257743	25,63764666	7,235305628	1,225256094	5,905137434	3,52353E-09	7,46975E-08	MGAM2	protein_coding
ENSG00000187688	35,83748957	2,090544382	0,353616802	5,91189211	3,382E-09	7,18748E-08	TRPV2	protein_coding
ENSG00000107859	20,53411021	3,195993551	0,53912272	5,928137387	3,0639E-09	6,57254E-08	PITX3	protein_coding
ENSG00000248596	17,33702581	3,650101571	0,615624398	5,929104795	3,04591E-09	6,53803E-08	ENSG00000248596	lncRNA
ENSG00000160808	19,47482869	3,620305935	0,610181725	5,933160216	2,97159E-09	6,3945E-08	MYL3	protein_coding
ENSG00000122176	14,38398387	4,094687547	0,689641463	5,93741497	2,89551E-09	6,23863E-08	FMOD	protein_coding
ENSG00000158825	29,59030788	2,569064319	0,429921985	5,97565235	2,29171E-09	5,03575E-08	CDA	protein_coding
ENSG00000142910	17,9936797	3,234368991	0,540854041	5,980114311	2,22981E-09	4,91231E-08	TINAGL1	protein_coding
ENSG00000118292	19,7771347	3,662715016	0,611423038	5,990475972	2,09228E-09	4,63613E-08	C1orf54	protein_coding
ENSG00000183876	18,97958051	3,181173314	0,526726621	6,03951497	1,54578E-09	3,5021E-08	ARSI	protein_coding
ENSG00000225950	31,75173152	2,486946931	0,411469186	6,044066032	1,50278E-09	3,40918E-08	NTF4	protein_coding
ENSG00000128040	30,1991571	2,226160489	0,368114199	6,047472488	1,47136E-09	3,34454E-08	SPINK2	protein_coding
ENSG00000179930	20,99924027	4,671474148	0,771378328	6,056009069	1,3954E-09	3,18454E-08	ZNF648	protein_coding
ENSG00000153253	30,5396611	2,447278575	0,403251735	6,068860627	1,28821E-09	2,95367E-08	SCN3A	protein_coding
ENSG00000224514	33,00729954	2,403601531	0,395526937	6,076960397	1,22482E-09	2,81208E-08	LINC00620	lncRNA
ENSG00000105357	53,33016608	6,320839759	1,037444198	6,092703365	1,1102E-09	2,56956E-08	MYH14	protein_coding
ENSG00000226435	27,62106964	2,37403373	0,389187697	6,099971162	1,06088E-09	2,46039E-08	ANKRD18DP	transcribed_unprocessed_pseudogene
ENSG00000205181	18,15922783	3,345558989	0,547176579	6,114221836	9,70293E-10	2,26254E-08	LINC00654	lncRNA
ENSG00000174844	32,17621975	2,533471207	0,413137599	6,132269771	8,6634E-10	2,03956E-08	DNAH12	protein_coding
ENSG00000172005	30,09470292	3,423594163	0,557682858	6,138962514	8,30622E-10	1,96491E-08	MAL	protein_coding
ENSG00000250423	62,20598494	3,535176284	0,575606692	6,141652513	8,16674E-10	1,93325E-08	KIAA1210	protein_coding
ENSG00000179630	24,25067493	2,875149307	0,468061933	6,142668537	8,11465E-10	1,92224E-08	LACC1	protein_coding
ENSG00000177108	49,09942928	2,22888631	0,362644047	6,146209563	7,93564E-10	1,88374E-08	ZDHHC22	protein_coding
ENSG00000157423	33,53042305	2,629069551	0,426194857	6,168703134	6,88524E-10	1,65617E-08	HYDIN	protein_coding
ENSG00000183287	37,4782	2,25986459	0,366058944	6,173499172	6,67949E-10	1,61082E-08	CCBE1	protein_coding
ENSG00000174348	31,01562375	3,03586635	0,488937114	6,209114142	5,32841E-10	1,30657E-08	PODN	protein_coding
ENSG00000196337	29,55256542	2,308557256	0,370924758	6,223788541	4,85291E-10	1,19412E-08	CGB7	protein_coding
ENSG00000163637	26,71897658	2,848918894	0,456782315	6,236929055	4,46244E-10	1,1026E-08	PRICKLE2	protein_coding
ENSG00000089163	29,83367976	2,256434927	0,361137145	6,248138581	4,15373E-10	1,03244E-08	SIRT4	protein_coding
ENSG00000163239	40,12742841	2,318318971	0,370345744	6,259877442	3,8528E-10	9,61826E-09	TDRD10	protein_coding
ENSG00000130635	16,67456414	4,174641189	0,665164044	6,276107714	3,47154E-10	8,74282E-09	COL5A1	protein_coding
ENSG00000232871	24,69194789	3,25068531	0,517590818	6,280415327	3,3767E-10	8,53531E-09	SEC1P	transcribed_unitary_pseudogene
ENSG00000142408	46,81587608	2,078603726	0,330494686	6,289371101	3,18755E-10	8,09297E-09	CACNG8	protein_coding
ENSG00000229089	23,66742858	2,576836226	0,409033399	6,299818631	2,97994E-10	7,59398E-09	ANKRD20A8P	transcribed_unprocessed_pseudogene
ENSG00000188672	32,22698711	2,358844349	0,374366831	6,300890332	2,95941E-10	7,55287E-09	RHCE	protein_coding
ENSG00000169064	23,59455979	2,836400718	0,449979143	6,303404858	2,91177E-10	7,44792E-09	ZBBX	protein_coding
ENSG00000205108	32,88877491	2,265385342	0,359149372	6,307641113	2,8332E-10	7,2632E-09	FAM205A	protein_coding
ENSG00000177692	29,7365375	2,572899243	0,406838963	6,324122022	2,54676E-10	6,59295E-09	DNAJC28	protein_coding
ENSG00000204653	26,26243523	3,876316782	0,612519535	6,328478623	2,4759E-10	6,42405E-09	ASPDH	protein_coding
ENSG00000154262	18,28891883	3,275074675	0,516690676	6,338559665	2,31923E-10	6,03117E-09	ABCA6	protein_coding
ENSG00000258711	38,85073234	-2,517145869	0,393113474	-6,403102498	1,52251E-10	4,08316E-09	ENSG00000258711	lncRNA
ENSG00000042832	19,46992073	3,587572472	0,55983762	6,408237569	1,47211E-10	3,95729E-09	TG	protein_coding
ENSG00000013016	46,74533462	3,101474137	0,483739558	6,411454435	1,44138E-10	3,8838E-09	EHD3	protein_coding
ENSG00000206549	19,44217915	2,659672145	0,414497876	6,416612241	1,3934E-10	3,76434E-09	ENSG00000206549	protein_coding
ENSG00000143851	51,14985771	2,122691485	0,330610953	6,420511683	1,35817E-10	3,67402E-09	PTPN7	protein_coding
ENSG00000215910	36,05932083	2,498271087	0,387446998	6,448033157	1,13311E-10	3,08957E-09	C1orf167	protein_coding
ENSG00000019505	29,27880986	5,627970038	0,872441931	6,450824789	1,11243E-10	3,04044E-09	SYT13	protein_coding
ENSG00000178031	156,5166925	-2,30641963	0,356432444	-6,470846489	9,74554E-11	2,69582E-09	ADAMTSL1	protein_coding

ENSG00000137474	33,00823762	5,682035206	0,878008357	6,471504699	9,70317E-11	2,68844E-09	MYO7A	protein_coding
ENSG00000132031	24,177042	2,776869864	0,427939423	6,488932112	8,64469E-11	2,40683E-09	MATN3	protein_coding
ENSG00000142677	24,77013325	3,649354879	0,555325753	6,571557067	4,97918E-11	1,44493E-09	IL22RA1	protein_coding
ENSG00000188783	19,84793134	3,431846448	0,521756146	6,577491179	4,78452E-11	1,3908E-09	PRELP	protein_coding
ENSG00000145794	42,54085402	2,608152973	0,394501135	6,611268623	3,8104E-11	1,13455E-09	MEGF10	protein_coding
ENSG00000173210	50,74359587	2,195734189	0,33202277	6,613203635	3,76091E-11	1,12079E-09	ABLIM3	protein_coding
ENSG00000174403	52,80696112	2,120065638	0,320548618	6,613866093	3,7441E-11	1,11675E-09	MIR1-1HG-AS1	lncRNA
ENSG00000105639	32,80124725	3,792212082	0,572849163	6,619913802	3,59408E-11	1,07294E-09	JAK3	protein_coding
ENSG00000075673	44,15268178	2,530963501	0,380916712	6,644401315	3,04452E-11	9,20623E-10	ATP12A	protein_coding
ENSG00000229739	45,05494248	2,158228046	0,319918885	6,746172697	1,51796E-11	4,77684E-10	PDC-AS1	lncRNA
ENSG00000232022	28,74561382	3,089777989	0,456065476	6,774856142	1,2453E-11	3,95876E-10	FAAHP1	transcribed_unprocessed_pseudogene
ENSG00000134668	29,04793169	2,633077506	0,386128064	6,819181895	9,15604E-12	2,94893E-10	SPOCD1	protein_coding
ENSG00000128052	19,90832369	3,72599596	0,545385178	6,831861427	8,38198E-12	2,72189E-10	KDR	protein_coding
ENSG00000214456	41,97462221	2,276393454	0,332734201	6,84147721	7,83807E-12	2,55564E-10	PLIN5	protein_coding
ENSG00000267750	38,643259	2,377843084	0,347270488	6,847236289	7,52903E-12	2,45956E-10	RUNDC3A-AS1	lncRNA
ENSG00000118640	22,29578137	3,348281509	0,488915114	6,848390269	7,46856E-12	2,44213E-10	VAMP8	protein_coding
ENSG00000229015	35,85310318	-2,457735915	0,356718377	-6,889849455	5,58515E-12	1,85277E-10	ENSG00000229015	transcribed_unprocessed_pseudogene
ENSG00000159708	30,24040437	2,861135037	0,413866219	6,913188144	4,73881E-12	1,58427E-10	LRRC36	protein_coding
ENSG00000087245	30,5706812	3,609154426	0,520719629	6,931089644	4,17611E-12	1,4085E-10	MMP2	protein_coding
ENSG00000158683	62,092147	2,074747056	0,299173823	6,934921766	4,06446E-12	1,37355E-10	PKD1L1	protein_coding
ENSG00000277152	62,17946397	2,328145934	0,331878581	7,015053299	2,29861E-12	7,98789E-11	ENSG00000277152	lncRNA
ENSG00000184497	46,24431071	2,174648442	0,308477826	7,049610251	1,7942E-12	6,31856E-11	TMEM255B	protein_coding
ENSG00000139567	43,82437808	6,136665628	0,867134407	7,076948597	1,47363E-12	5,27631E-11	ACVRL1	protein_coding
ENSG00000139973	32,67936719	3,630305908	0,511917692	7,091581247	1,32588E-12	4,80751E-11	SYT16	protein_coding
ENSG00000084636	35,074958	4,216819058	0,59310313	7,109756883	1,16248E-12	4,24644E-11	COL16A1	protein_coding
ENSG00000189120	32,01089699	4,041323034	0,568401858	7,10997506	1,16064E-12	4,24425E-11	SP6	protein_coding
ENSG00000061337	59,85536919	2,012587946	0,282797878	7,116701019	1,10541E-12	4,06831E-11	LZTS1	protein_coding
ENSG00000141505	65,37748526	2,003898209	0,280775017	7,13702463	9,53728E-13	3,54429E-11	ASGR1	protein_coding
ENSG00000178662	120,6374984	2,274315823	0,316595645	7,183661106	6,7869E-13	2,56951E-11	CSRNP3	protein_coding
ENSG00000141485	62,41351389	2,885228186	0,399894408	7,214975072	5,39439E-13	2,07902E-11	SLC13A5	protein_coding
ENSG00000205336	39,367077	8,643357402	1,195233956	7,231519286	4,7762E-13	1,85536E-11	ADGRG1	protein_coding
ENSG00000157103	37,79162273	3,085147316	0,426342255	7,236316079	4,61036E-13	1,79681E-11	SLC6A1	protein_coding
ENSG00000197177	46,26780958	2,832246733	0,390472211	7,253388734	4,06471E-13	1,5916E-11	ADGRA1	protein_coding
ENSG00000118514	50,0537567	2,332366495	0,319588064	7,298040053	2,9199E-13	1,1619E-11	ALDH8A1	protein_coding
ENSG00000036530	55,10944722	2,399103953	0,326364736	7,350990124	1,96744E-13	8,01486E-12	CYP46A1	protein_coding
ENSG00000023445	41,60082792	2,675423053	0,363755797	7,354997699	1,9093E-13	7,79655E-12	BIRC3	protein_coding
ENSG00000255346	25,71613081	4,246980377	0,57741436	7,35516931	1,90685E-13	7,79582E-12	NOX5	protein_coding
ENSG00000090530	30,38554495	2,707073656	0,366867784	7,378880829	1,59626E-13	6,60473E-12	P3H2	protein_coding
ENSG00000121101	59,48247272	2,12178204	0,284865959	7,448352356	9,45131E-14	4,01225E-12	TEX14	protein_coding
ENSG00000160111	47,09603347	3,184110344	0,419372427	7,592560078	3,13646E-14	1,39719E-12	CPAMD8	protein_coding
ENSG00000166546	78,01003981	2,014608618	0,264120507	7,627611502	2,39143E-14	1,07649E-12	BEAN1	protein_coding
ENSG00000163075	48,0094885	2,574812736	0,337350599	7,632453438	2,30328E-14	1,03954E-12	CFAP221	protein_coding
ENSG00000142233	39,12327797	4,006043343	0,522300358	7,669999231	1,71997E-14	7,87675E-13	NTN5	protein_coding
ENSG00000263528	56,46283572	2,492935332	0,324464016	7,683241323	1,55113E-14	7,12253E-13	IKBKE	protein_coding
ENSG00000244509	58,21111876	4,393357226	0,571694319	7,684801261	1,53235E-14	7,05517E-13	APOBEC3C	protein_coding
ENSG00000115596	42,17552152	3,591400354	0,465872164	7,708982485	1,26825E-14	5,87867E-13	WNT6	protein_coding
ENSG00000006283	99,62152007	2,052991387	0,265801707	7,723770507	1,12938E-14	5,27778E-13	CACNA1G	protein_coding
ENSG00000154263	43,91788027	2,176112449	0,281102497	7,741348696	9,83677E-15	4,62207E-13	ABCA10	protein_coding
ENSG00000136449	87,9712079	2,069204587	0,265157945	7,803668064	6,01332E-15	2,88884E-13	MYCBPAP	protein_coding
ENSG00000172782	67,74159269	2,066561662	0,264472388	7,813903293	5,54436E-15	2,67855E-13	FADS6	protein_coding
ENSG00000262943	40,98324488	3,000035329	0,383640936	7,819904091	5,28636E-15	2,5867E-13	ALOX12P2	transcribed_unprocessed_pseudogene
ENSG00000073861	48,81304673	3,186451202	0,406683726	7,835207659	4,68071E-15	2,31678E-13	TBX21	protein_coding
ENSG00000174460	258,9302181	2,274716395	0,28823912	7,89176845	2,97934E-15	1,50507E-13	ZCCHC12	protein_coding
ENSG00000184454	33,60086497	3,8885764	0,492123414	7,901628509	2,75283E-15	1,39889E-13	NCMAP	protein_coding
ENSG00000146005	53,5039062	2,333193181	0,295018561	7,908631819	2,60233E-15	1,32437E-13	PSD2	protein_coding
ENSG00000163898	58,26773528	2,141459533	0,270717716	7,910304361	2,5676E-15	1,30864E-13	LIPH	protein_coding
ENSG00000170703	37,55692612	2,847294868	0,359933514	7,910613375	2,56124E-15	1,30734E-13	TTLL6	protein_coding
ENSG00000105122	56,06307414	2,954920951	0,372939427	7,92332679	2,31239E-15	1,18561E-13	RASAL3	protein_coding
ENSG00000115828	65,09475549	3,448507315	0,433158271	7,961310096	1,70227E-15	8,83859E-14	QPCT	protein_coding
ENSG00000161082	65,2355563	2,118344933	0,265904405	7,966565766	1,63145E-15	8,54355E-14	CELF5	protein_coding
ENSG00000136931	70,29593293	2,59263914	0,323430767	8,016055993	1,09195E-15	5,83409E-14	NR5A1	protein_coding
ENSG00000175264	31,28823814	4,376628736	0,544840302	8,032864598	9,52227E-16	5,11148E-14	CHST1	protein_coding
ENSG00000175793	42,4702083	2,348392001	0,292054197	8,040945911	8,91476E-16	4,82311E-14	SFN	protein_coding
ENSG00000118160	44,13915242	3,908683984	0,485094291	8,057575726	7,78224E-16	4,24386E-14	SLC8A2	protein_coding
ENSG00000119922	62,44794168	2,575103514	0,31858024	8,083061008	6,3161E-16	3,46638E-14	IFIT2	protein_coding
ENSG00000175170	90,79893133	4,378174196	0,541085588	8,091463338	5,89521E-16	3,25099E-14	FAM182B	lncRNA
ENSG00000117971	62,58754707	2,298642488	0,28336857	8,111847001	4,98561E-16	2,77166E-14	CHRNB4	protein_coding
ENSG00000205413	53,57885864	2,426725106	0,296809104	8,176046724	2,93308E-16	1,66569E-14	SAMD9	protein_coding
ENSG00000042493	56,3573933	2,493501655	0,303811505	8,207397073	2,26035E-16	1,2987E-14	CAPG	protein_coding
ENSG00000116299	49,06309292	2,464356645	0,298745904	8,249005644	1,59718E-16	9,2624E-15	ELAPOR1	protein_coding
ENSG00000027644	80,42153086	2,427474037	0,293058553	8,283239009	1,1987E-16	6,99259E-15	INSRR	protein_coding
ENSG00000188162	80,73670584	5,211160142	0,624576291	8,343512578	7,21158E-17	4,30199E-15	OTOG	protein_coding
ENSG00000175463	132,4470049	2,029254217	0,241673832	8,396665062	4,59338E-17	2,76902E-15	TBC1D10C	protein_coding
ENSG00000129910	32,90903158	5,05385563	0,597932831	8,452212973	2,85833E-17	1,74455E-15	CDH15	protein_coding
ENSG00000128340	38,41452422	2,971875477	0,350361145	8,482320382	2,20747E-17	1,35696E-15	RAC2	protein_coding
ENSG00000108370	44,34048549	5,373654525	0,629951826	8,530262644	1,46016E-17	9,13958E-16	RGS9	protein_coding
ENSG00000108176	59,84432933	2,823835656	0,327617327	8,619311085	6,73581E-18	4,36758E-16	DNAJC12	protein_coding
ENSG00000104848	63,94631845	2,569859534	0,297204573	8,646769813	5,29777E-18	3,47455E-16	KCNA7	protein_coding
ENSG00000186510	37,96503529	4,026114825	0,465497822	8,649051903	5,1929E-18	3,41249E-16	CLCNKA	protein_coding
ENSG00000109339	32,47301188	3,582111902	0,412714612	8,679391993	3,9789E-18	2,66043E-16	MAPK10	protein_coding
ENSG00000157087	55,70403505	3,040025041	0,349050416	8,709415318	3,05446E-18	2,08292E-16	ATP2B2	protein_coding
ENSG00000158859	106,1566088	2,216733428	0,253842512	8,732711506	2,48639E-18	1,70571E-16	ADAMTS4	protein_coding
ENSG00000141837	71,50100902	5,150190721	0,587370409	8,768216177	1,81519E-18	1,2553E-16	CACNA1A	protein_coding
ENSG00000238230	92,84575109	-2,038251617	0,229206673	-8,892636452	5,96758E-19	4,2823E-17	LINC00391	lncRNA
ENSG00000205795	71,71236047	2,03222982	0,228521424	8,892950974	5,95071E-19	4,27914E-17	CYS1	protein_coding
ENSG00000110169	75,48935649	2,243991299	0,250835067	8,946082871	3,68321E-19	2,68803E-17	HPX	protein_coding
ENSG00000229474	91,7226923	2,191501898	0,242887139	9,022716927	1,83481E-19	1,38321E-17	PATL2	protein_coding
ENSG00000122733	60,94914948	2,896918763	0,319097649	9,078471042	1,10111E-19	8,4684E-18	PHF24	protein_coding
ENSG00000103196	47,2828225	3,634050896	0,399446526	9,097715621	9,22517E-20	7,14295E-18	CRISPLD2	protein_coding

ENSG00000141314	83,81559369	2,381454263	0,260909195	9,127521408	7,00855E-20	5,51376E-18	RHBDL3	protein_coding
ENSG00000100346	58,1955877	2,746432243	0,300036384	9,153663978	5,50346E-20	4,37991E-18	CACNA1I	protein_coding
ENSG00000184908	67,12193238	2,455078391	0,268060027	9,158688904	5,25316E-20	4,21988E-18	CLCNKB	protein_coding
ENSG00000177301	72,10759455	3,301459697	0,355989768	9,274029746	1,79243E-20	1,52183E-18	KCNA2	protein_coding
ENSG00000127084	69,7869279	2,303190383	0,247352174	9,311381184	1,26181E-20	1,07933E-18	FGD3	protein_coding
ENSG00000101204	61,10926766	3,680833138	0,394994193	9,318701899	1,17772E-20	1,015E-18	CHRNA4	protein_coding
ENSG00000163235	64,59063083	3,1174664	0,333268121	9,35422924	8,42115E-21	7,34997E-19	TGFA	protein_coding
ENSG00000123453	57,41692086	4,510769368	0,480543688	9,386803912	6,18485E-21	5,43966E-19	SARDH	protein_coding
ENSG00000260401	117,6464787	2,344138508	0,247588632	9,467876154	2,85591E-21	2,58471E-19	ENSG00000260401	lncRNA
ENSG00000160539	69,62110148	3,343389441	0,351386951	9,514836653	1,81999E-21	1,68268E-19	PLPP7	protein_coding
ENSG00000183508	71,91176717	3,056928579	0,318052399	9,611399232	7,15697E-22	6,78153E-20	TENT5C	protein_coding
ENSG00000169129	58,87727685	2,307683002	0,239093304	9,651809391	4,82959E-22	4,64033E-20	AFAP1L2	protein_coding
ENSG00000076641	119,3526687	2,159381515	0,223676631	9,654032719	4,72598E-22	4,55354E-20	PAG1	protein_coding
ENSG00000184584	98,85294963	2,315996469	0,239517367	9,669430243	4,06636E-22	3,94011E-20	STING1	protein_coding
ENSG00000205517	49,01626637	2,549030129	0,263342878	9,679510418	3,68476E-22	3,61117E-20	RGL3	protein_coding
ENSG00000135253	52,53387293	3,628811857	0,374293643	9,695093468	3,16348E-22	3,15437E-20	KCP	protein_coding
ENSG00000182040	63,42443178	3,230019295	0,333048161	9,698354999	3,06399E-22	3,06408E-20	USH1G	protein_coding
ENSG00000063127	104,0879335	2,54890574	0,260132968	9,798472517	1,14301E-22	1,17034E-20	SLC6A16	protein_coding
ENSG00000168477	126,1223942	2,347965363	0,238473553	9,84581028	7,14611E-23	7,47313E-21	TNXB	protein_coding
ENSG00000062038	123,7972813	2,103502487	0,213404981	9,856857488	6,40218E-23	6,75696E-21	CDH3	protein_coding
ENSG00000115423	52,50804759	3,102894842	0,310754538	9,985034687	1,77239E-23	1,94233E-21	DNAH6	protein_coding
ENSG00000075651	61,36425676	2,682893992	0,266343924	10,07304374	7,26929E-24	8,25643E-22	PLD1	protein_coding
ENSG00000010310	139,6725342	2,047699798	0,200814744	10,19695943	2,04577E-24	2,45356E-22	GIPR	protein_coding
ENSG00000148344	95,33202855	2,274173045	0,221916395	10,24788208	1,20965E-24	1,47135E-22	PTGES	protein_coding
ENSG00000146216	135,7642231	2,130170321	0,207806167	10,25075603	1,17421E-24	1,43334E-22	TTBK1	protein_coding
ENSG00000110324	94,82902807	2,108118091	0,205046013	10,28119521	8,56587E-25	1,0569E-22	IL10RA	protein_coding
ENSG00000149599	145,4656593	2,049862656	0,19901013	10,30029304	7,0247E-25	8,76197E-23	DUSP15	protein_coding
ENSG00000258776	162,1454258	-2,112017907	0,20475864	-10,3146705	6,04884E-25	7,62799E-23	ENSG00000258776	lncRNA
ENSG00000108846	62,03154257	2,997976157	0,290089836	10,33464734	4,91216E-25	6,24044E-23	ABCC3	protein_coding
ENSG00000185513	80,55294616	3,022677991	0,292193965	10,34476531	4,41998E-25	5,67827E-23	L3MBTL1	protein_coding
ENSG00000131409	93,08016815	2,211547836	0,212870168	10,38918634	2,77712E-25	3,62198E-23	LRRC4B	protein_coding
ENSG00000188596	88,37659924	2,989369638	0,286157144	10,44660145	1,5187E-25	2,03488E-23	CFAP54	protein_coding
ENSG00000028137	104,3543289	2,171457006	0,206955663	10,4923778	9,36417E-26	1,27968E-23	TNFRSF1B	protein_coding
ENSG00000162745	129,9929893	2,274184197	0,216496065	10,5045059	8,23529E-26	1,13027E-23	OLFML2B	protein_coding
ENSG00000159307	139,8992222	2,064467058	0,193792288	10,6529887	1,68855E-26	2,49651E-24	SCUBE1	protein_coding
ENSG00000243323	86,20875275	4,126911333	0,387117265	10,66062329	1,55551E-26	2,31981E-24	PTPRVP	unitary_pseudogene
ENSG00000054179	78,94974214	2,868752134	0,266408306	10,76825334	4,86136E-27	7,64906E-25	ENTPD2	protein_coding
ENSG00000134202	133,3099966	2,361082266	0,218872765	10,78746486	3,94523E-27	6,26506E-25	GSTM3	protein_coding
ENSG00000197748	67,56042686	3,008532314	0,278376884	10,80740712	3,17518E-27	5,08934E-25	CFAP43	protein_coding
ENSG00000184988	129,3448816	2,053583328	0,189414581	10,84173834	2,18283E-27	3,5654E-25	TMEM106A	protein_coding
ENSG00000116981	66,65257918	2,917527792	0,261657391	11,150183	7,14593E-29	1,27001E-26	NT5C1A	protein_coding
ENSG00000224511	155,5899012	-2,124868248	0,190522683	-11,15283604	6,93598E-29	1,23912E-26	LINC00365	lncRNA
ENSG00000187800	90,42599617	3,215107194	0,287288139	11,19122845	4,50153E-29	8,08414E-27	PEAR1	protein_coding
ENSG00000143195	87,79582677	4,058208833	0,360497252	11,25725316	2,13304E-29	3,99811E-27	ILDR2	protein_coding
ENSG00000275395	73,76799311	3,117960185	0,276320043	11,28387268	1,5765E-29	3,03794E-27	FCGBP	protein_coding
ENSG00000196169	81,5569693	3,515211555	0,310141353	11,33422397	8,88158E-30	1,76097E-27	KIF19	protein_coding
ENSG00000088340	342,2642788	2,144194724	0,187139294	11,45774724	2,15035E-30	4,49752E-28	FER1L4	transcribed_unitary_pseudogene
ENSG00000108387	149,3357064	2,346521464	0,204663963	11,46524005	1,97212E-30	4,17566E-28	SEPTIN4	protein_coding
ENSG00000167207	87,68723697	2,520513097	0,219146861	11,50147937	1,29674E-30	2,81515E-28	NOD2	protein_coding
ENSG00000169291	128,2900061	2,301029214	0,199808718	11,51616022	1,09377E-30	2,42049E-28	SHE	protein_coding
ENSG00000104967	260,3824365	2,040583623	0,17702903	11,52683053	9,66362E-31	2,16648E-28	NOVA2	protein_coding
ENSG00000092068	84,38062302	3,143740537	0,271623796	11,57387748	5,58981E-31	1,26978E-28	SLC7A8	protein_coding
ENSG00000222078	80,39645476	7,2013185	0,614424975	11,72041957	1,00175E-31	2,41978E-29	RN7SKP110	misc_RNA
ENSG00000007516	219,3636063	2,11187215	0,179152029	11,78815645	4,49268E-32	1,12484E-29	BAIAP3	protein_coding
ENSG00000091536	86,52467451	4,059151241	0,344165709	11,79417687	4,1827E-32	1,05493E-29	MYO15A	protein_coding
ENSG00000183914	93,0181299	3,768417214	0,316930359	11,89036363	1,32827E-32	3,53186E-30	DNAH2	protein_coding
ENSG00000226455	57,5752517	5,343384667	0,447301657	11,94581909	6,82744E-33	1,82959E-30	ENSG00000226455	lncRNA
ENSG00000224940	146,5391305	2,512517422	0,209387553	11,9993638	3,58038E-33	9,74687E-31	PRRT4	protein_coding
ENSG00000148357	138,5209186	4,125761119	0,343057913	12,0264275	2,58089E-33	7,13927E-31	HMCN2	protein_coding
ENSG00000132692	133,439112	2,29347351	0,189976463	12,07240873	1,47745E-33	4,15395E-31	BCAN	protein_coding
ENSG00000072163	170,1484635	2,181652915	0,179714967	12,13951711	6,52097E-34	1,91176E-31	LIMS2	protein_coding
ENSG00000124772	128,4547558	3,097003512	0,252996243	12,24130238	1,87008E-34	5,57786E-32	CPNE5	protein_coding
ENSG00000173175	162,3054381	2,021347012	0,165113207	12,24218856	1,84977E-34	5,56568E-32	ADCY5	protein_coding
ENSG00000107738	81,07975052	3,823598001	0,312292897	12,24362781	1,81725E-34	5,51623E-32	VSIR	protein_coding
ENSG00000132561	193,3244932	2,136843112	0,174030233	12,27857412	1,18061E-34	3,64829E-32	MATN2	protein_coding
ENSG00000166750	165,4849974	2,155172181	0,175508998	12,27955378	1,1664E-34	3,63715E-32	SLFN5	protein_coding
ENSG00000161681	243,6822011	2,020059557	0,164054526	12,31334241	7,67764E-35	2,48444E-32	SHANK1	protein_coding
ENSG00000135636	111,6216189	3,042833254	0,24471757	12,43406127	1,70735E-35	5,74154E-33	DYSF	protein_coding
ENSG00000152527	187,3898911	2,478505771	0,197000348	12,58122536	2,67843E-36	9,28007E-34	PLEKHH2	protein_coding
ENSG00000095303	109,9574369	3,371878681	0,26629336	12,66227095	9,56906E-37	3,49179E-34	PTGS1	protein_coding
ENSG00000116016	132,9139965	2,458495127	0,194127513	12,66433123	9,32113E-37	3,45753E-34	EPAS1	protein_coding
ENSG00000186642	110,1477549	3,205346315	0,252309297	12,70403572	5,61574E-37	2,11676E-34	PDE2A	protein_coding
ENSG00000165272	226,5041508	2,005955201	0,156919261	12,78335872	2,03105E-37	7,82777E-35	AQP3	protein_coding
ENSG00000168016	95,5553875	2,95922304	0,230309133	12,84891746	8,72223E-38	3,47885E-35	TRANK1	protein_coding
ENSG00000156042	198,0241579	2,189970516	0,170020114	12,88065552	5,78422E-38	2,39042E-35	CFAP70	protein_coding
ENSG00000248485	112,6022887	3,278538902	0,253965086	12,90940797	3,98352E-38	1,66633E-35	PCP4L1	protein_coding
ENSG00000185101	130,0980583	3,048258803	0,235213233	12,95955489	2,07448E-38	9,00717E-36	ANO9	protein_coding
ENSG00000117115	241,0472994	2,640119836	0,203642983	12,96445275	1,94614E-38	8,55829E-36	PADI2	protein_coding
ENSG00000105559	170,0018099	2,543318296	0,195699781	12,99602017	1,28878E-38	5,89418E-36	PLEKHA4	protein_coding
ENSG00000154188	230,9354495	-2,223247022	0,170204496	-13,06221087	5,41325E-39	2,57889E-36	ANGPT1	protein_coding
ENSG00000006611	112,5025588	4,477258488	0,337052996	13,28354454	2,88412E-40	1,57029E-37	USH1C	protein_coding
ENSG00000142609	171,8405596	2,669427171	0,199698659	13,36727638	9,39172E-41	5,28107E-38	CFAP74	protein_coding
ENSG00000197558	349,4068344	2,588262449	0,192966383	13,41302259	5,07284E-41	2,94921E-38	SSPOP	transcribed_unitary_pseudogene
ENSG00000125637	149,1336634	2,617862982	0,194118702	13,48588754	1,89373E-41	1,15994E-38	PSD4	protein_coding
ENSG00000103485	233,7215818	2,314452459	0,171448505	13,49940299	1,57648E-41	9,83182E-39	QPRT	protein_coding
ENSG00000163520	107,4344374	4,032282863	0,297710991	13,5442862	8,56423E-42	5,54268E-39	FBLN2	protein_coding
ENSG00000178038	272,5129616	2,029702527	0,14940886	13,58488735	4,92289E-42	3,24731E-39	ALS2CL	protein_coding
ENSG00000111424	145,6025348	2,423576183	0,178388718	13,58592744	4,85345E-42	3,24731E-39	VDR	protein_coding
ENSG00000172350	158,7075642	2,583557558	0,189645388	13,62309722	2,9192E-42	2,0435E-39	ABCG4	protein_coding

ENSG00000132470	325,6261233	2,093802101	0,151748719	13,79782391	2,62662E-43	2,09525E-40	ITGB4	protein_coding
ENSG00000135472	151,1634624	3,084655129	0,21944599	14,05655728	7,02201E-45	6,17594E-42	FAIM2	protein_coding
ENSG00000198959	107,8962046	3,779685009	0,264530156	14,28829537	2,58869E-46	2,6116E-43	TGM2	protein_coding
ENSG00000143603	306,6005563	2,546642217	0,176011297	14,46862939	1,91245E-47	1,98785E-44	KCNN3	protein_coding
ENSG00000143850	242,2820346	3,460383699	0,237623719	14,56245071	4,86808E-48	5,566E-45	PLEKHA6	protein_coding
ENSG00000171992	168,4756059	3,246330062	0,215949596	15,03281374	4,47583E-51	6,67502E-48	SYNPO	protein_coding
ENSG00000116983	186,6042251	3,378122749	0,218315325	15,47359418	5,23034E-54	9,44242E-51	HPCAL4	protein_coding
ENSG00000185567	1345,784646	2,199629236	0,14206726	15,48301303	4,51807E-54	8,60969E-51	AHNAK2	protein_coding
ENSG00000104888	160,2314889	4,518038669	0,286691555	15,75923182	5,935E-56	1,19751E-52	SLC17A7	protein_coding
ENSG00000129244	195,248251	2,969516861	0,185951966	15,96926843	2,09225E-57	5,12617E-54	ATP1B2	protein_coding
ENSG00000021300	207,9633419	3,012957121	0,185106114	16,27691842	1,43937E-59	3,79783E-56	PLEKHB1	protein_coding
ENSG00000161642	254,3086608	3,213360662	0,197355457	16,2820968	1,32258E-59	3,7805E-56	ZNF385A	protein_coding
ENSG00000168071	207,1734025	3,258224501	0,199433799	16,33737366	5,35086E-60	1,66854E-56	CCDC88B	protein_coding
ENSG00000177098	244,1807548	3,416420959	0,195737513	17,45409407	3,20421E-68	1,22119E-64	SCN4B	protein_coding
ENSG00000079308	651,7935611	2,377366245	0,131680861	18,05399985	7,3379E-73	3,59568E-69	TNS1	protein_coding
ENSG00000064300	429,5951477	3,204947199	0,176178998	18,19142598	6,03494E-74	3,45008E-70	NGFR	protein_coding
ENSG00000065361	671,8656728	2,042016772	0,110659112	18,45321849	4,913E-76	4,21302E-72	ERBB3	protein_coding
ENSG00000149260	516,7088159	2,615986706	0,140111298	18,67077626	8,55995E-78	9,78717E-74	CAPN5	protein_coding
ENSG00000173546	642,8241115	3,048026416	0,142992049	21,31605524	8,0571E-101	1,38183E-96	CSPG4	protein_coding
ENSG00000112182	5821,12613	6,543670056	0,235710853	27,76142881	1,2683E-169	4,3504E-165	BACH2	protein_coding

ensembl_gen	baseMean	log2FoldChan	lfcSE	stat	pvalue	padj	hgnc_symbol	gene_biotype
ENSG0000006	72,0148223	2,13657439	0,19188031	11,1349327	8,4809E-29	1,1869E-25	ATP2B3	protein_coding
ENSG0000009	181,797553	2,49487047	0,16123438	15,4735642	5,2328E-54	1,904E-50	ICAM1	protein_coding
ENSG0000010	419,04356	5,81775947	0,21974201	26,4754089	1,861E-154	1,693E-150	COL20A1	protein_coding
ENSG0000011	51,5009176	2,22081995	0,32985015	6,73281474	1,6641E-11	5,9363E-09	SLC1A2	protein_coding
ENSG0000014	476,557032	6,42189139	0,22993142	27,9295953	1,167E-171	2,123E-167	SELENBP1	protein_coding
ENSG0000016	61,9733039	6,34050348	0,54016097	11,7381741	8,1221E-32	1,6418E-28	GSG1L	protein_coding
ENSG0000017	25,5228236	2,61215503	0,39589651	6,5980755	4,1653E-11	1,3778E-08	OVOL1	protein_coding
ENSG0000018	160,320746	3,01578344	0,15774664	19,1178935	1,792E-81	8,1505E-78	SLC47A2	protein_coding
ENSG0000019	60,2173656	2,38944825	0,28584732	8,35917661	6,3158E-17	4,1037E-14	PAX5	protein_coding
ENSG0000021	46,0810244	7,14429191	0,74936147	9,53383934	1,5157E-21	1,3131E-18	ENSG0000021	processed_pseudogene
ENSG0000025	642,07648	2,84227997	0,14100493	20,1573088	2,3217E-90	1,408E-86	SMIM3	protein_coding
ENSG0000025	88,1157176	2,76706422	0,20435044	13,5407796	8,983E-42	2,3347E-38	CTXND1	protein_coding
ENSG0000026	50,87651	3,56778688	0,30660649	11,6363712	2,6923E-31	4,4693E-28	MALT1-AS1	lncRNA
