# Supplementary material for: Selective persistence of HIV-1-infected T cell clones can occur through immune reprogramming driven by defective, transcriptionally active proviruses
Source: PLoS Pathog. 2026 Oct 20;22(10):e1014618. doi: 10.1371/journal.ppat.1014618 (PMC13682663; doi:10.1371/journal.ppat.1014618)
Supplement: Supplementary file 2 [file ppat.1014618.s009.pdf]

name	5'-3' sequence	description
oligo 1	gcggacaataggatccctctctccttctagcctc	Fwd HIV reporter 5' LTR-leader Gibson
oligo 2	taacttcgtatagcatacattatacgaagttattggaagggctaatttgg	Rev HIV reporter 5' LTR-leader Gibson
oligo 3	atgtatgctatacgaagttatcggaccgcccgggctagcatgcggccttgacggccttc	Fwd pMK backbone_step1 Gibson
oligo 4	ggatcctattgtccgcggtcgacctgggcctcatg	Rev pMK backbone_step1 Gibson
oligo 5	gctcgctcatctctctccttctagcctccg	Fwd pMK backbone_step2 Gibson
oligo 6	tctctagcaggatcctattgtccgcgg	Rev pMK backbone_step2 Gibson
oligo 7	aaggagagagatgagcgagctgattaagg	Fwd HIV reporter bfp-2A-tat Gibson
oligo 8	ccttccactattccttcgggcctgtc	Rev HIV reporter bfp-2A-tat Gibson
oligo 9	aataggatcctgctagagattttccacac	Fwd HIV reporter 3' LTR Gibson
oligo 10	cccgaaggaatagtggaagggctaatttggtc	Rev HIV reporter 3' LTR Gibson
oligo 11	gatccataacttcgtatagcatacattatacgaagttatccgc	Fwd HIV reporter 3' loxP Gibson
oligo 12	ggataacttcgtataatgtatgctatacgaagttatg	Rev HIV reporter 3' loxP Gibson
oligo 13	tccccgcggcacaatctcctgcctcagcc	Fwd 3' BACH2 homology arm
oligo 14	tccccgcggggagacagggtatcactttg	Rev 3' BACH2 homology arm
oligo 15	tcccccgggcttcctcagctcaaaaggag	Fwd 5' BACH2 homology arm
oligo 16	gtacggaccgcagcaggaagaggttgcagt	Rev 5' BACH2 homology arm
oligo 17	gtccgnnnnnnnncgtatcgatatatctcg	Fwd HIV reporter 8bp random barcode
oligo 18	gaccgagatatatcgatacgnnnnnnnncg	Rev HIV reporter 8bp random barcode
oligo 19	agcgagctgattaaggagaac	Fwd BACH2 clone validation HIV reporter
oligo 20	ctctagtctaggatctactggc	Rev BACH2 clone validation HIV reporter
oligo 21	tggtaacaggattagcagagc	Fwd BACH2 clone validation pMK backbone
oligo 22	tcgttttctggtgctgtatgg	Rev BACH2 clone validation pMK backbone
oligo 23	agcgagctgattaaggagaac	Fwd Southern bfp-specific probe
oligo 24	cagccgagtgttttcttctgc	Rev Southern bfp-specific probe
oligo 25	tcctactttctttttggctttc	Fwd Southern genomic BACH2-specific probe
oligo 26	aagttacgctatatttatac	Rev Southern genomic BACH2-specific probe
oligo 27	cagtccactgcaccaacatc	Fwd qPCR BACH2 exon6
oligo 28	tctccacgatcaaagtcacg	Rev qPCR BACH2 exon6
oligo 29	tgaccagcggaaaaaggata	Probe qPCR BACH2 exon6 (FAM/BHQ1)
oligo 30	tgagggctccaggaatgag	Fwd qPCR BACH2 exon4/5
oligo 31	ttaggatgcagggaactggg	Rev qPCR BACH2 exon4/5
oligo 32	tccatgtctgaatggattgccagagcc	Probe qPCR BACH2 exon4/5 (FAM/BHQ1)
oligo 33	ACCCAGCGCAGGCAA	Fwd qPCR STAT5B exon1
oligo 34	GGTTTACAATCTCGCTCG	Rev qPCR STAT5B exon2
oligo 35	AAGCCAGACAGCTGGGCC	Probe qPCR STAT5B exon1/2 (FAM/BHQ1)
oligo 36	GCGTCATGGAGTACCAC	Fwd qPCR STAT5B exon10
oligo 37	CACTGAACTGGGATTCAAAC	Rev qPCR STAT5B exon11
oligo 38	CTTCTGTCACCGACTCTGC	Probe qPCR STAT5B exon10/11 (HEX/BHQ1)
oligo 39	CTGAGCTTGTATCCACACAG	Rev STAT5B qPCR exon 2
oligo 40	TCTCTAGCAGTGGCGCCC	Fwd qPCR HIV LTR
oligo 41	TCAGCAAGCCGAGTCCTGCGTCGAGA	Probe qPCR LTR/STAT5B chimeric transcript
oligo 42	ACAACAGGGGGATCATCA	Fwd ChIP-qPCR BACH2 binding site 1
oligo 43	GCCGATACGAATGACTCA	Rev ChIP-qPCR BACH2 binding site 1
oligo 44	CAGTAGCTAACCAGCCCA	Fwd ChIP-qPCR BACH2 binding site 2
oligo 45	CCCTGTTTTTGTTTTTGCC	Rev ChIP-qPCR BACH2 binding site 2
oligo 46	CATACACTTGATCATTTCCC	Fwd ChIP-qPCR BACH2 binding site 3&4
oligo 47	AACCCCCAATTACCTCAG	Rev ChIP-qPCR BACH2 binding site 3&4
oligo 48	GAACAAAGAAAGACACGGA	Fwd ChIP-qPCR BACH2 intron 1 region (neg. ctrl)
oligo 49	GCTATGTTGAGTTGATGG	Rev ChIP-qPCR BACH2 intron 1 region (neg. ctrl)
oligo 50	GAGCCCGAGATTCACTCAAC	Fwd ChIP-qPCR HOXC13 (neg. ctrl)
oligo 51	TTATGCCCAGTTTTGGGGTA	Rev ChIP-qPCR HOXC13 (neg. ctrl)
oligo 52	AATACTCGAGAGGTCTTGCACTGTGTTATTATTAGAAGTCACAATTCTATTGATACTGATGAGTCATTCGTATCGGCGGAAACCAAGAACTTCCATCAACATCATGGGTACCAATA	Fwd cloning BACH2 binding site 1 in Gluc reporter
oligo 53	TATTGGTACCCATGATGTTGATGGAAGTTCTTGGTTTCCGCCGATACGAATGACTCATCAGTATCAATAGAATTGTGACTTCTAATAATAACACAGTGCAAGACCTCTCGAGTATT	Rev cloning BACH2 binding site 1 in Gluc reporter
oligo 54	AATACTCGAGCCTAAAAATCTGCAGGAACACTGGTTGTCTGGAACTGAGAATACATTTTCCCACAGAAATAGGGCCAGGCCAATGAGCTAGGATTCCTTGATAGTCCGTGGGTACCAATA	Fwd cloning BACH2 neg. control site in Gluc reporter
oligo 55	TATTGGTACCCACGGACTATCAAGGAATCCTAGCTCATTGGCCTGGCCCTATTTCTGTGGGAAAATGTATTCTCAGTTCCAGACAACCAGTGTTCCTGCAGATTTTTAGGCTCGAGTATT	Rev cloning BACH2 neg. control site in Gluc reporter
